# A new antibiotic traps lipopolysaccharide in its intermembrane transporter

**DOI:** 10.1038/s41586-023-06799-7

**Published:** 2024-01-03

**Authors:** Karanbir S. Pahil, Morgan S. A. Gilman, Vadim Baidin, Thomas Clairfeuille, Patrizio Mattei, Christoph Bieniossek, Fabian Dey, Dieter Muri, Remo Baettig, Michael Lobritz, Kenneth Bradley, Andrew C. Kruse, Daniel Kahne

**Affiliations:** 1 Department of Chemistry and Chemical Biology, Harvard University, Cambridge, MA USA; 2 Department of Biological Chemistry and Molecular Pharmacology, Harvard Medical School, Boston, MA USA; 3 Departments of Immunology, Infectious Disease and Ophthalmology (I2O), Medicinal Chemistry and Lead Discovery, Roche Pharma Research and Early Development, Roche Innovation Center Basel, Basel, Switzerland

**Keywords:** Cryoelectron microscopy, Mechanism of action, Transporters, Target validation, Antibiotics

## Abstract

Gram-negative bacteria are extraordinarily difficult to kill because their cytoplasmic membrane is surrounded by an outer membrane that blocks the entry of most antibiotics. The impenetrable nature of the outer membrane is due to the presence of a large, amphipathic glycolipid called lipopolysaccharide (LPS) in its outer leaflet^1^. Assembly of the outer membrane requires transport of LPS across a protein bridge that spans from the cytoplasmic membrane to the cell surface. Maintaining outer membrane integrity is essential for bacterial cell viability, and its disruption can increase susceptibility to other antibiotics^2–6^. Thus, inhibitors of the seven lipopolysaccharide transport (Lpt) proteins that form this transenvelope transporter have long been sought^7–9^. A new class of antibiotics that targets the LPS transport machine in *Acinetobacter* was recently identified. Here, using structural, biochemical and genetic approaches, we show that these antibiotics trap a substrate-bound conformation of the LPS transporter that stalls this machine. The inhibitors accomplish this by recognizing a composite binding site made up of both the Lpt transporter and its LPS substrate. Collectively, our findings identify an unusual mechanism of lipid transport inhibition, reveal a druggable conformation of the Lpt transporter and provide the foundation for extending this class of antibiotics to other Gram-negative pathogens.

## Abstract (web summary)

A mechanism of lipid transport inhibition has been identified for a class of peptide antibiotics effective against resistant *Acinetobacter* strains, which may have applications in the inhibition of other Gram-negative pathogens.

## Main

**Fig. 1:**
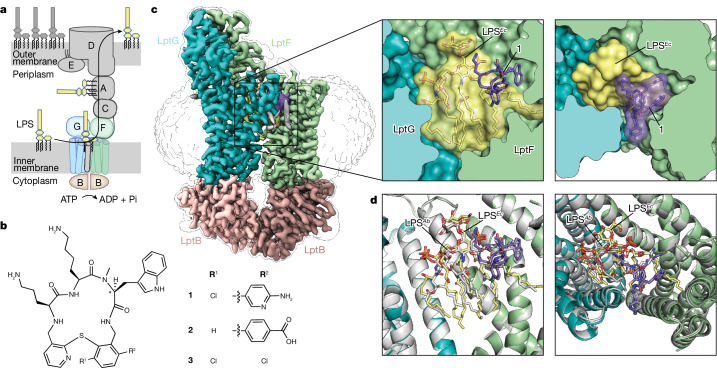
Macrocyclic peptides block LPS transport by binding to the inner membrane complex. **a**, Schematic of the seven protein LPS transport machine. **b**, Structures of macrocyclic peptides that prevent growth of *Acinetobacter* strains. The compounds were selected from those prepared during the drug discovery–development process^32^: compound **1** (RO7196472) was a potent hit found early in the discovery process; compound **2** (Zosurabalpin) is a clinical candidate; and compound **3** (RO7075573) was an important preclinical lead. Compound 2a, the epimer of compound 2 at the starred position, is an inactive compound that was used as a negative control. **c**, Cryo-EM structure of the inner membrane *A. baylyi* LptB_2_FG complex bound to LPS and **1**. The drug has 500 Å^2^ contact with LptFG and 230 Å^2^ contact with LPS. Postprocessing of the map was carried out using DeepEMhancer. The unsharpened map is shown as an outline to show the positioning of the detergent micelle. Inset shows a close-up view of LPS and **1**. LptB, LptF, LptG, LPS and **1** are coloured tan, green, blue, yellow and purple, respectively. **d**, Cryo-EM structure of *Acinetobacter* LptB_2_FG with *Acinetobacter* LPS and **1** bound in the lumen of the transporter in white superimposed with the structure of *Acinetobacter* LptB_2_FG bound to *E. coli* LPS and **1** (LptF, LptG, LPS and **1** are coloured green, blue, yellow and purple respectively). The overall r.m.s.d. is 0.44 Å over 7,999 atoms.

The outer membrane of Gram-negative bacteria is an asymmetric bilayer containing phospholipids in its inner leaflet and lipopolysaccharide (LPS) in its outer leaflet^1,10–13^. The biosynthesis of LPS is completed inside the cell at the inner membrane. LPS must be extracted from the inner membrane, moved across the periplasmic compartment and delivered through the outer membrane to the cell surface^1,12,14–16^ (Fig. 1a). To accomplish outer membrane biogenesis, the inner membrane components of the lipopolysaccharide transporter, LptB_2_FGC, form a subcomplex that couples ATP hydrolysis to extraction of LPS from the bilayer^17–22^, passing it to the protein bridge formed by the connected β-jellyroll domains of LptF, LptC, the soluble periplasmic protein LptA and the periplasmic portion of the integral membrane protein LptD (refs. ^6,17,23–29^). LptD, together with its associated lipoprotein LptE, form the outer membrane translocon that serves as a conduit for LPS to pass directly from the bridge into the outer leaflet of the outer membrane^5,23,25,30,31^.

**Extended Data Fig. 1:**
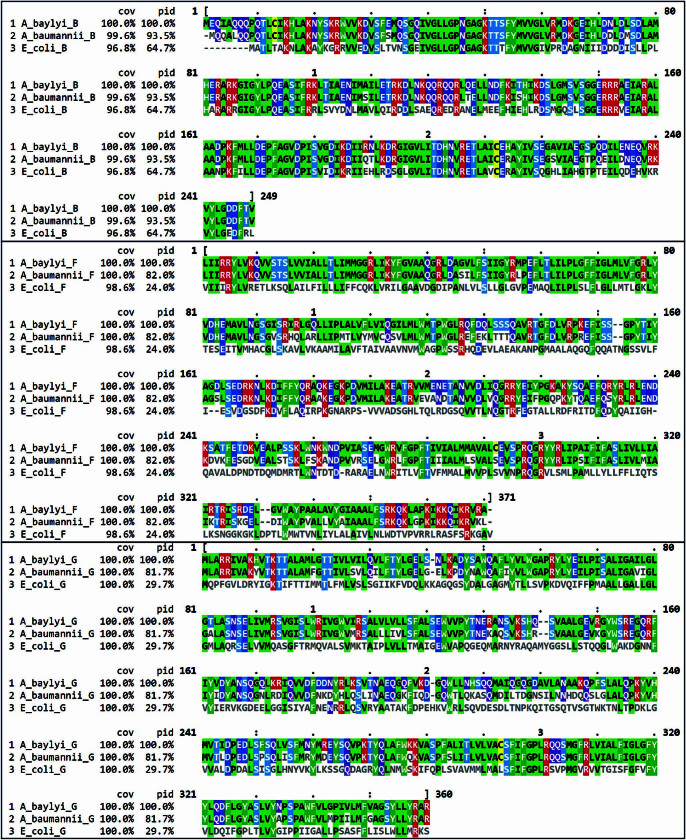
Sequences of Lpt proteins from *Acinetobacter baylyi ADP1, Acinetobacter baumannii 19606* and *E. coli* K12. Percent coverage and identities when compared to the *Acinetobacter baylyi ADP1* sequence are shown. Alignments were made using ClustalO and then coloured in MView (https://www.ebi.ac.uk/Tools/msa/clustalo/).

A family of macrocyclic peptides (Fig. 1b, **1**–**3**) proposed to target the lipopolysaccharide transport machinery was recently identified (**1**, RO7196472; **2**, Zosurabalpin; **3**, RO7075573)^32^. These macrocyclic peptides all have potent and selective activity against *Acinetobacter* strains, including carbapenem-resistant *A. baumannii*. One macrocyclic peptide, Zosurabalpin (compound **2**), is now undergoing clinical trials. Resistance mutations to compound **2** map to *lptFG* and biochemical experiments have shown that **2** blocks LPS extraction from liposomes containing *Acinetobacter* LptB_2_FGC (ref. ^32^). To determine the molecular mechanism by which these antibiotics inhibit transport of LPS, we sought to solve a structure of LptB_2_FG bound to a macrocyclic peptide. *A. baumannii* proteins expressed poorly and tended to aggregate, so we instead solved structures of *A. baylyi* LptB_2_FG with compounds **1**–**3** to high resolution using cryo-electron microscopy (cryo-EM). *A. baylyi* LptB_2_FG is about 85% identical to *A. baumannii* (Extended Data Fig. 1), is similarly susceptible to macrocyclic peptides **1**–**3** and mutations that provide resistance in *A. baumannii* also confer resistance to *A. baylyi* (see below; ref. ^32^).

## Drug binds LPS within the transporter

**Extended Data Fig. 2:**
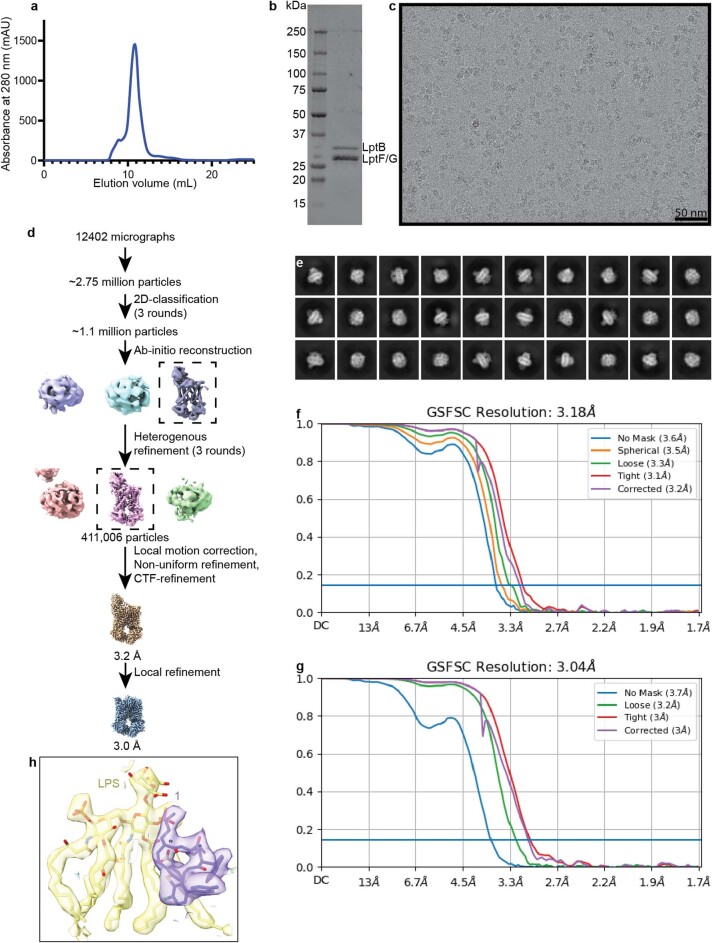
Expression, purification and cryo-EM data processing for LptB_2_FG in complex with LPS and 1. **a**. Representative size-exclusion chromatogram of *Acinetobacter baylyi* LptB_2_FG. **b**. SDS–PAGE gel showing peak fractions from the size-exclusion chromatography of the complex in **a** and used in subsequent structural experiments described in this figure. Four independent purifications of this construct gave similar size-exclusion and SDS–PAGE results. **c**. Representative (n = 12,402) micrographs of LptB_2_FG in complex with **1** and LPS embedded in vitreous ice. **d**. Scheme of three-dimensional classification and refinement of cryo-EM particle images. The initial models used for 3D classification were generated by ab initio reconstruction in cryoSPARC. **e**. Representative selected two-dimensional class averages of cryo-EM particle images. (**f**-**g**) Gold-standard Fourier shell correlation (FSC) curves calculated with different masks in cryoSPARC from the overall (**f**) and locally refined (**g**) structures of **1**-bound LptB_2_FG. The resolution was determined at FSC = 0.143 (horizontal blue line). The final corrected mask gave an overall resolution of 3.0 Å. **h**. LPS and **1** fit in the final locally refined, sharpened map. Post processing of the map was done using DeepEMhancer.

**Extended Data Fig. 3:**
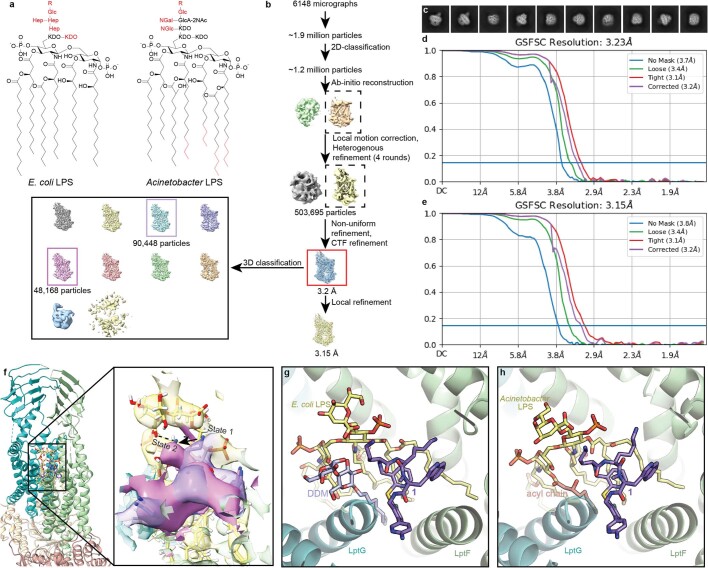
Cryo-EM data processing and analysis for *Acinetobacter* LptB_2_FG with *Acinetobacter* LPS in the presence and absence of 1. **a**. Structures of *E. coli* and *Acinetobacter* LPS. The parts that were not modelled in the structures presented in this work are coloured in red. **b**. Scheme of three-dimensional classification and refinement of cryo-EM particle images for *Acinetobacter* LptB_2_FG in the presence of *Acinetobacter* LPS and **1**. The initial models used for 3D classification were generated by ab initio reconstruction in cryoSPARC. **c**. Representative selected two-dimensional class averages of *Acinetobacter* LptB_2_FG in the presence of *Acinetobacter* LPS and **1**. (**d**-**e**) Gold-standard Fourier shell correlation (FSC) curves calculated with different masks in cryoSPARC from the overall (**d**) and locally refined (**e**) structures of *Acinetobacter* LptB_2_FG in the presence of *Acinetobacter* LPS and **1**. The resolution was determined at FSC = 0.143 (horizontal blue line). The final corrected mask gave an overall resolution of 3.15 Å. **f**. 3D classification without alignments of the dataset detailed in (b–e) revealed conformational flexibility in the lysine of **1**. There is a major population (purple box in **b**, transparent surface in **f**, state 1) in which said lysine is in contact with the 2’-phosphate of LPS and a minor population (pink box in **b**, solid surface in **f**, state 2) where the lysine is oriented towards the carboxylate of the branched KDO from LPS. This KDO is conserved in *E. coli* and *Acinetobacter* LPS, as shown in panel a. (**g**-**h**). There is an ordered DDM molecule in the *Acinetobacter* LptB_2_FG + **1** structure obtained with *E. coli* LPS, but not in the structure obtained using *Acinetobacter* LPS. LptF, LptG, LPS and **1** are coloured green, blue, yellow and purple. The structure obtained using *E. coli* LPS is shown in **g** with the DDM coloured violet. The *Acinetobacter* LPS acyl chain that overlaps with the location of the DDM highlighted in salmon (**h**).

We first solved a structure of LptB_2_FG in the presence of LPS and compound **1** to 3.0 Å resolution. Unexpectedly, compound **1** was found to form extensive contacts with both LptB_2_FG and a bound LPS molecule, which is a unique mode of inhibition (Fig. 1c and Extended Data Fig. 2). Because LptB_2_FG was heterologously expressed in *Escherichia coli*, the structure we obtained contained copurified *E. coli* LPS. To rule out the possibility that structural differences between *E. coli* and *Acinetobacter* LPS affect how LPS binds or how compound **1** interacts with the LptB_2_FG–LPS complex, we also purified LptB_2_FG from *A. baylyi* to trap the native *Acinetobacter* LPS (Extended Data Fig. 3). We obtained a structure of *A. baylyi* LptB_2_FG with *Acinetobacter* LPS and **1** and found only minor differences compared to the complex with *E. coli* LPS (Fig. 1d). The sugars attached to the *Acinetobacter* LPS are better resolved and an ordered detergent molecule observed in the structure solved using *E*. *coli* LPS is displaced to accommodate the extra lipid chain that is present on *Acinetobacter* LPS (Extended Data Fig. 3g,h). Notably, the drug contact interface is nearly identical regardless of the LPS chemotype (see below) and the protein conformation is also unaltered.

**Fig. 2:**
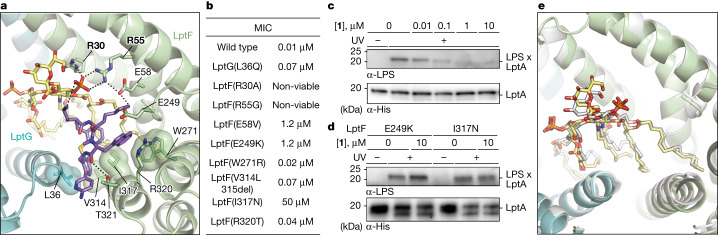
Compound 1 binds an intermediate transport state with LPS bound in the transporter. **a**, View of the ternary complex highlighting key contacts from LptF (bolded) to both LPS and **1**. LptF, LptG, LPS and **1** are coloured green, blue, yellow and purple, respectively. **b**, Table showing the MICs of **1** against *A. baylyi* containing various LptF variants. MIC values were consistent across three cultures started from individual colonies. **c**,**d**, **1** inhibits LPS transport to LptA by wild-type LptB_2_FGC (**c**) but not by LptB_2_F^E249K^GC or LptB_2_F^I317N^GC (**d**). Lipopolysaccharide transport from LptB_2_FGC to LptA modified with a photocrosslinkable amino acid (I36*p*BPA) was monitored in the presence of the indicated dose of **1** by exposing the samples to UV light after 60 min of transport, quenching by addition of SDS-loading buffer, PAGE to separate LPS-LptA adducts from LPS and western blotting against LPS. Data shown are representative of experiments conducted in biological triplicate. **e**, Cryo-EM structure of LptB_2_FG with LPS bound in the lumen of the transporter in white superimposed with the LptB_2_FG-LPS-**1** structure, which is coloured as in **a**. The two structures have an r.m.s.d. of 0.31 Å over 8,010 atoms.

The compound binding pocket is lined by side chains of several amino acids in the transmembrane (TM) helices of LptF (Glu58, Glu249, Trp271, Val314, Ile317, Arg320 and Thr321) and LptG (Leu36) (Fig. 2). Previous morbidostat experiments carried out with compound **2** identified mutations that altered the corresponding residues in *A. baumannii* LptFG (ref. ^32^). To verify that alterations in these residues reduced susceptibility to the macrocyclic peptide antibiotics, we constructed *A. baylyi* strains encoding each LptFG variant identified from the morbidistat experiments and measured minimum inhibitory concentrations (MICs) for compounds **1**–**3** against *A. baylyi*. All the mutations decreased *A. baylyi* susceptibility to compounds **1**, **2** and **3** (Fig. 2b and Supplementary Table 1), some by two to three orders of magnitude. We also purified two *A. baylyi* LptB_2_FGC complexes with individual LptF substitutions (E249K or I317N) that conferred high-level resistance to the macrocyclic peptides (Fig. 2b) and tested inhibition of LPS release in the presence of **1** (Fig. 2c,d). Because *E. coli* LPS is readily available and there are well-validated commercial antibodies to it, the biochemical experiments were conducted using *E. coli* LPS. Compound **1** blocked LPS release from the wild-type complex to LptA but did not block LPS release from either of these mutant complexes. These results confirm the importance of the contacts observed between the macrocyclic peptides and LptFG for inhibiting growth of *Acinetobacter* strains.

## Drug traps LPS during transport

**Extended Data Fig. 4:**
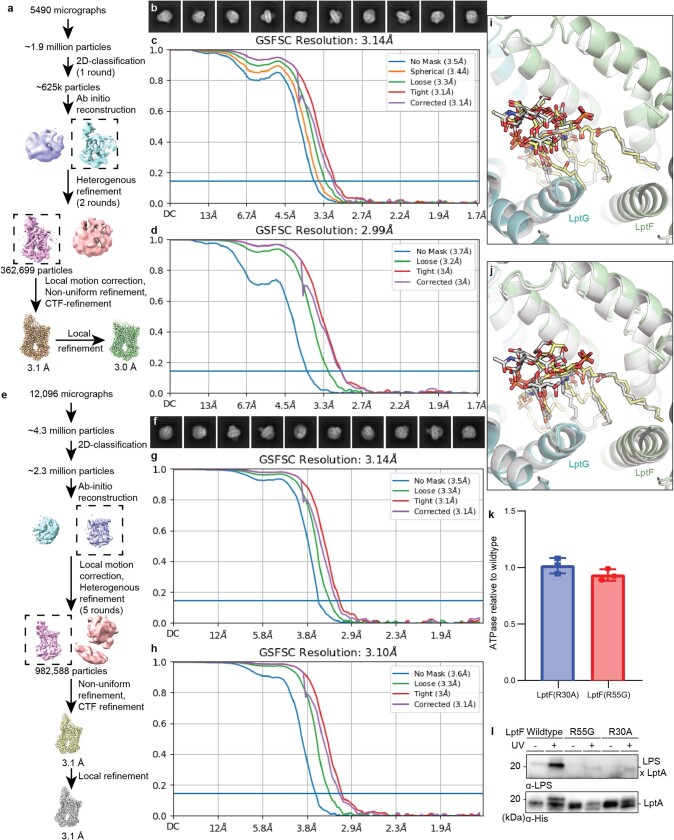
Cryo-EM data processing and analysis for drug-free *Acinetobacter baylyi* LptB_2_FG. (**a**-**d**). Processing for the structure of *Acinetobacter baylyi* LptB_2_FG in the presence of *E. coli* LPS. **a** Scheme of three-dimensional classification and refinement of cryo-EM particle images. The initial models used for 3D classification were generated by ab initio reconstruction in cryoSPARC. **b**. Representative selected two-dimensional class averages of cryo-EM particle images. (**c**-**d**) Gold-standard Fourier shell correlation (FSC) curves calculated with different masks in cryoSPARC from the overall (**c**) and locally refined (**d**) structures of drug-free LptB_2_FG. The resolution was determined at FSC = 0.143 (horizontal blue line). The final corrected mask gave an overall resolution of 3.0 Å. (**e**-**h**) Processing for the structure of *Acinetobacter baylyi* LptB_2_FG in the presence of *Acinetobacter baylyi* LPS. **e** Scheme of three-dimensional classification and refinement of cryo-EM particle images. The initial models used for 3D classification were generated by ab initio reconstruction in cryoSPARC. **f**. Representative selected two-dimensional class averages of cryo-EM particle images. (**g**-**h**) Gold-standard Fourier shell correlation (FSC) curves calculated with different masks in cryoSPARC from the overall (**g**) and locally refined (**h**) structures of drug-free LptB_2_FG. The resolution was determined at FSC = 0.143 (horizontal blue line). The final corrected mask gave an overall resolution of 3.1 Å. **i**. Cryo-EM structure of *Acinetobacter* LptB_2_FG with *Acinetobacter* LPS bound in the lumen of the transporter in white superimposed with the structure of *Acinetobacter* LptB_2_FG bound to **1** and *Acinetobacter* LPS (LptF, LptG and LPS are coloured green, blue and yellow, respectively). The overall r.m.s.d. is 0.37 over 7714 atoms. **j**. Cryo-EM structure of *Acinetobacter* LptB_2_FG with *Acinetobacter* LPS bound in the lumen of the transporter in white superimposed with the structure of *Acinetobacter* LptB_2_FG bound to *E. coli* LPS (LptF, LptG and LPS are coloured green, blue and yellow, respectively). The overall r.m.s.d. is 0.51 over 8138 atoms. (**k**-**l**). LptB_2_F(R55G)G and LptB_2_F(R30A)G have comparable ATPase hydrolysis rates to wild-type LptB_2_FG **(k)** but are unable to transport LPS to LptA **(l)**. ATPase rates were measured using liposomes containing LptB_2_FG complexes and LPS and the presented data represent averages and standard deviations of results determined using three different proteoliposomes preparations for each LptF variant. Inorganic phosphate release was measured using a molybdate assay. LPS transport from LptB_2_FG to LptA^I36*p*BPA^ in the presence of LptC-ΔTM was measured by detecting UV-dependent LptA-LPS crosslinks by LPS immunoblotting. Data shown is representative of experiments conducted in biological triplicate.

The LptB_2_FG–LPS–**1** structure suggested that **1** traps an intermediate state of the LPS-bound Lpt transporter. To assess whether the conformation of LPS in the drug-bound structure reflects an on-pathway intermediate, we solved structures of *A. baylyi* LptB_2_FG bound to either *E. coli* or *Acinetobacter* LPS but now in the absence of **1** (Fig. 2e and Extended Data Fig. 4). In both cases, the overall conformation and contacts of LPS to the transporter are nearly identical to those determined in the presence of compound **1** (Fig. 2e). These findings are consistent with a mechanism in which compound **1** binds a pre-existing, LPS-loaded state of the transporter complex, identifying this state as a druggable conformation for antibiotic development.

We observed contacts from LptF (Arg30 and Arg55) to the same phosphate group on LPS that is coordinated by a primary amine from **1** (bold in Fig. 2a). To assess their functional importance, we separately mutated them to Ala and Gly, respectively (LptF R30A and LptF R55G). Neither of these variants produced viable cells in *A. baylyi*, suggesting that these residues are critical for protein folding or function. We found that we could express and purify complexes with these LptF substitutions, and both of the resulting LptB_2_FG variants had ATPase activity comparable to wild type but neither transferred LPS to LptA (Extended Data Fig. 4). Therefore, we concluded that both LptF Arg30 and Arg55 are critical for the function of the complex in *Acinetobacter*, probably because they help to position LPS during transport.

**Fig. 3:**
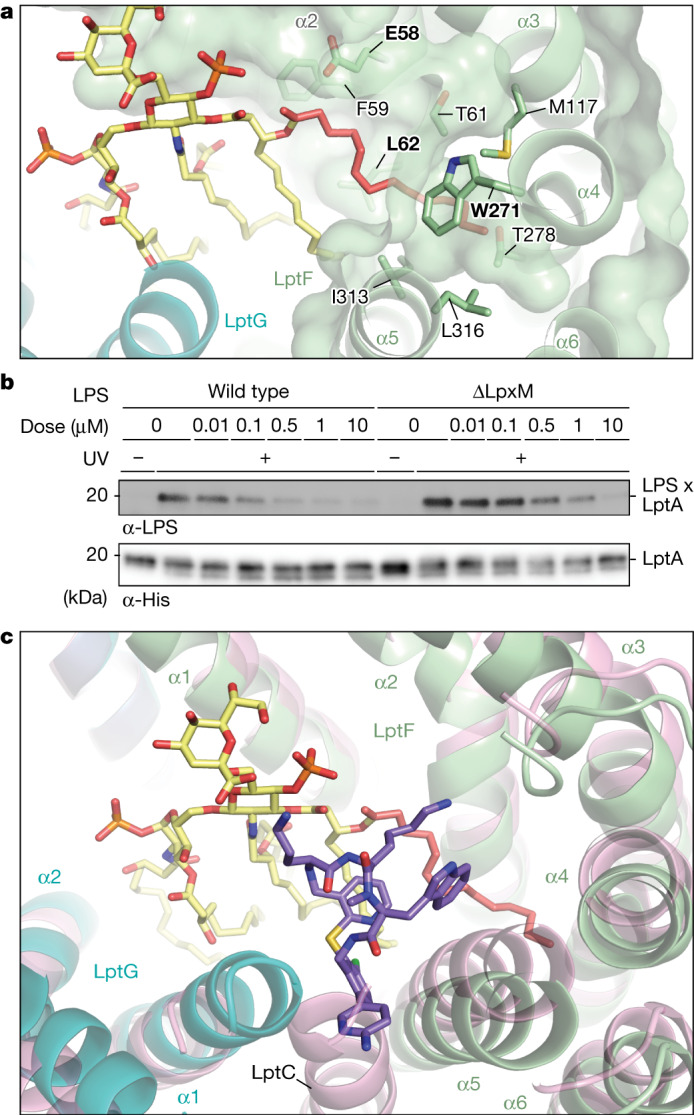
A binding pocket is created for **1** in LptB_2_FGC by moving TM helices of LptC and LptF. **a**, The acyl chain that is added by LpxM, highlighted in salmon, nestles between LptF helices 2, 4 and 5. Residues contacting this acyl chain are labelled. Residues that contact this acyl chain and elicited resistance in spontaneous mutation studies^32^ are bolded. LptF is shown in green, LPS in yellow and LptG in blue. **b**, LPS isolated from a Δ*LpxM* strain renders LptB_2_FGC resistant to **1** (ref. ^36^). Lipopolysaccharide transport from LptB_2_FGC to LptA was measured as described in Fig. 2. Data shown are representative of experiments conducted in biological triplicate. **c**, Cryo-EM structure of *Acinetobacter* LptB_2_FGC superimposed with the structure of *Acinetobacter* LptB_2_FG in complex with LPS and **1**. The LptB_2_FGC structure is shown in pink, whereas the LptB_2_FG-**1**-LPS structure is coloured as in Fig. 2a. The observed positioning of the TM helix of LptC sterically clashes with the compound **1** binding site observed in the LptB_2_FG structures. The positioning of LptF helices 2–5 are also shifted relative to what was observed in the LptB_2_FG structures.

**Extended Data Fig. 5:**
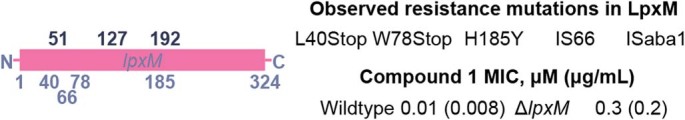
Loss of function mutations in LpxM render cells resistant to 1. 5 distinct mutations in LpxM were observed to provide resistance to **1**. The location of the mutated residues relative to the active site (active-site residues are bolded) is shown.

The ternary complex structure also showed an extensive interface between LPS and **1**. We therefore sought to determine whether changing LPS structure in *Acinetobacter* would affect the inhibitory potency of **1**. LPS biosynthesis involves more than 100 genes^1,12,33^ but mutations that confer decreased susceptibility to the compound were loss of function mutations in *lpxM* (Extended Data Fig. 5). LpxM performs the final acylation steps during LPS biosynthesis and of the total contact area of about 230 Å^2^ between LPS and **1**, 94 Å^2^ (41%) involves contact area between the drug and the acyl chain installed by LpxM (refs. ^34–36^) (Fig. 3a). In a biochemical reconstitution, transport of LPS isolated from an *E. coli* LpxM deletion strain was possible at 20-fold higher concentrations of compound **1** than that required to inhibit the transport of the matching wild-type LPS structure (Fig. 3b). *E. coli* and *Acinetobacter* Δ*lpxM* LPS chemotypes have identical acylation patterns. Consistent with the biochemical experiments using *E. coli* Δ*lpxM* LPS, we found the *A. baylyi lpxM* deletion strain to be 30-fold less susceptible to **1** than was wild type. Previous studies in *E. coli* and *A. baumannii* have established that the loss of the fatty acyl chains installed by LpxM does not prevent LPS transport to the outer membrane but does reduce outer membrane barrier function^37,38^. Consistent with this, our *A. baylyi ΔlpxM* strain was as much as 1,000-fold more susceptible to a broad range of other antibiotics (Supplementary Table 2). Because *ΔlpxM* strains are known to have reduced virulence, loss of *lpxM* may not cause reduced susceptibility to macrocyclic peptides in vivo^38,39^.

## LptC helix movement allows drug binding

**Extended Data Fig. 6:**
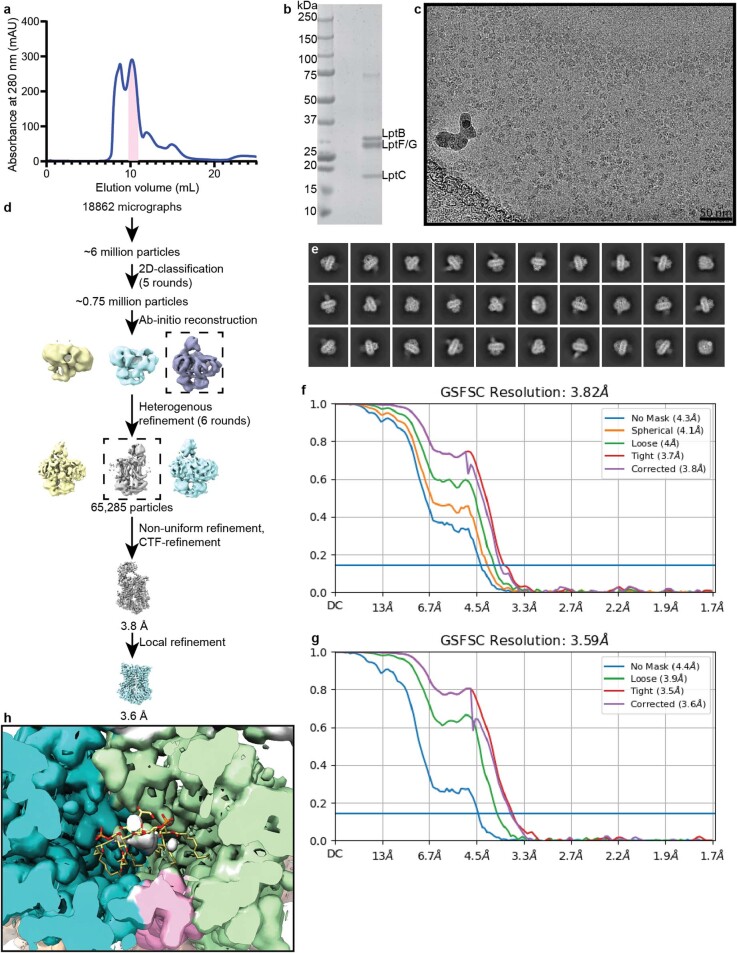
Cryo-EM data processing and analysis for LptB_2_FGC. **a**. Representative size-exclusion chromatogram of *Acinetobacter baylyi* LptB_2_FGC. **b**. SDS–PAGE gel showing peak fractions from the size-exclusion chromatography of the complex in **a** and used for the structural experiments. Three independent purifications of this construct gave similar size-exclusion and SDS–PAGE results. **c**. Representative (n = 18,862) micrograph of LptB_2_FGC embedded in vitreous ice. **d**. Scheme of three-dimensional classification and refinement of cryo-EM particle images. The initial models used for 3D classification were generated by ab initio reconstruction in cryoSPARC. **e**. Representative selected two-dimensional class averages of cryo-EM particle images. (**f**-**g**) Gold-standard Fourier shell correlation (FSC) curves calculated with different masks in cryoSPARC from the overall (**f**) and locally refined (**g**) structures of LptB_2_FGC. The resolution was determined at FSC = 0.143 (horizontal blue line). The final corrected mask gave an overall resolution of 3.6 Å. **h**. Electron density of the LptB_2_FGC structure with LptF coloured in green, LptG in blue and LptC in pink. The lipopolysaccharide positioning from our LptB_2_FG structure is overlayed with the LptB_2_FGC electron density. Post processing of the map was carried out using DeepEMHancer.

LptC, a member of the inner membrane Lpt complex, contains a periplasmic β-jellyroll domain that plays an essential role in transfer of LPS from LptF to LptA (refs. ^19,40^). LptC also contains a TM helix that exists in two states. In one, the TM helix is sandwiched between the TM helices of LptG (helix 1) and LptF (helices 5 and 6), which form the gate through which LPS enters the transporter lumen^19,41,42^. In a second state, the LptC helix has moved away from the transporter^42,43^. Movement of the LptC helix between these states is thought to be important in coordinating LPS transport with the catalytic cycle of ATP binding and hydrolysis^19,41–43^. The LptB_2_FG–LPS–**1** structure revealed that **1** protrudes into the gate formed between helix 5 of LptF and helix 1 of LptG, suggesting that its binding competes with the LptC TM helix for binding to the complex. We obtained a structure of *A. baylyi* LptB_2_FGC in the presence of LPS, although density for the LPS molecule was poorly resolved. In this structure, the LptC helix was sandwiched between LptG helix 1 and LptF helix 5, similar to the previously determined structures from other species^19,41,42,44^ (Fig. 3c and Extended Data Fig. 6). Despite several attempts, we were unable to obtain an LptB_2_FGC complex in which **1** was also bound. These data are compatible with a model in which binding of **1** and binding of the TM helix of LptC at the LptFG gate are mutually exclusive. Superimposing the LptB_2_FG–LPS–**1** structure with the LptB_2_FGC–LPS structure identified shifts in the conformation of LptFG, particularly in helices 4, 5 and 6 of LptF (Fig. 3). This conformation of LptF creates a pocket between helices 4 and 5 that better accommodates acyl chain 6 of LPS. Although the specific sequence of events is uncertain, we propose that C-helix movement away from LptFG during the transport cycle permits LPS to bind in the intermediate transport state observed in the structure of the LptB_2_FG–LPS complex and that this is the state required for inhibitor recognition.

**Fig. 4:**
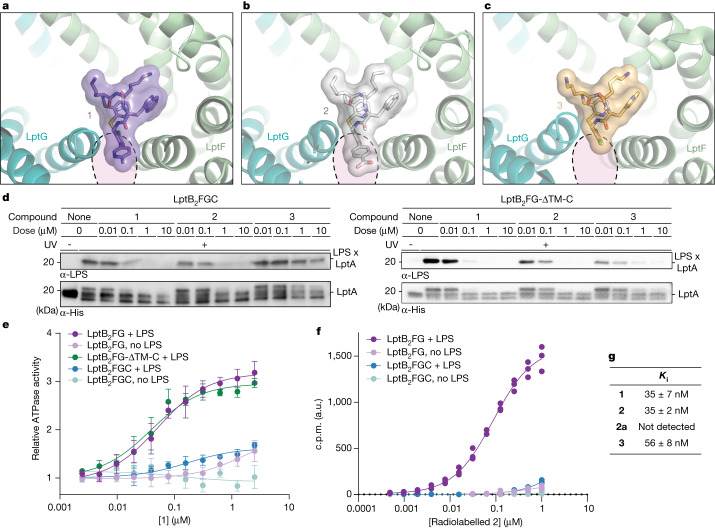
Macrocyclic peptide antibiotics bind an LPS-bound intermediate state in which the TM helix of LptC has moved from the lumen. **a**–**c**, Cryo-EM structures of **1** (**a**), **2** (**b**) and **3** (**c**) bound to LptB_2_FG. The observed positioning of the LptC TM helix from Fig. 3d is highlighted in pink. **d**, Transport of LPS to LptA by LptB_2_FGC is not inhibited by **3** in vitro but LPS transport by LptB_2_FG-ΔTM-C is inhibited by **1**, **2** and **3** in vitro. Data shown are representative of experiments conducted in biological triplicate. **e**, **1** treatment increases the ATPase activity of LptB_2_FG, LptB_2_FGC and LptB_2_FG-ΔTM-C in an LPS-dependent manner. This effect is reduced in the presence of the TM helix of LptC. ATP hydrolysis was monitored by measuring concentrations of inorganic phosphate. Experiments were conducted in biological triplicate and data are presented as mean values ± standard deviation. **f**, **2** binds Lpt in the presence of LPS and absence of LptC. The binding of radiolabelled **2** to His-tagged LptB_2_FG and LptB_2_FGC in the presence and absence of LPS was measured in a SPA. Data are presented as counts per minute (c.p.m.) in arbitrary units (a.u.) and are from three biological replicates. **g**, The cellular activity of **1**-derivatives correlates to their observed binding to LptB_2_FG through SPA. The ability of **1**-derivatives to displace radiolabelled **2** from His-tagged LptB_2_FG was measured in the presence of LPS. Active compounds **1**–**3** showed potent binding to LptB_2_FG, whereas the inactive control compound **2a** did not. **2a** is the epimer of **2** at the highlighted (*) carbon; Fig. 1. Uncertainties represent the standard deviation of three biological replicates.

**Extended Data Fig. 7:**
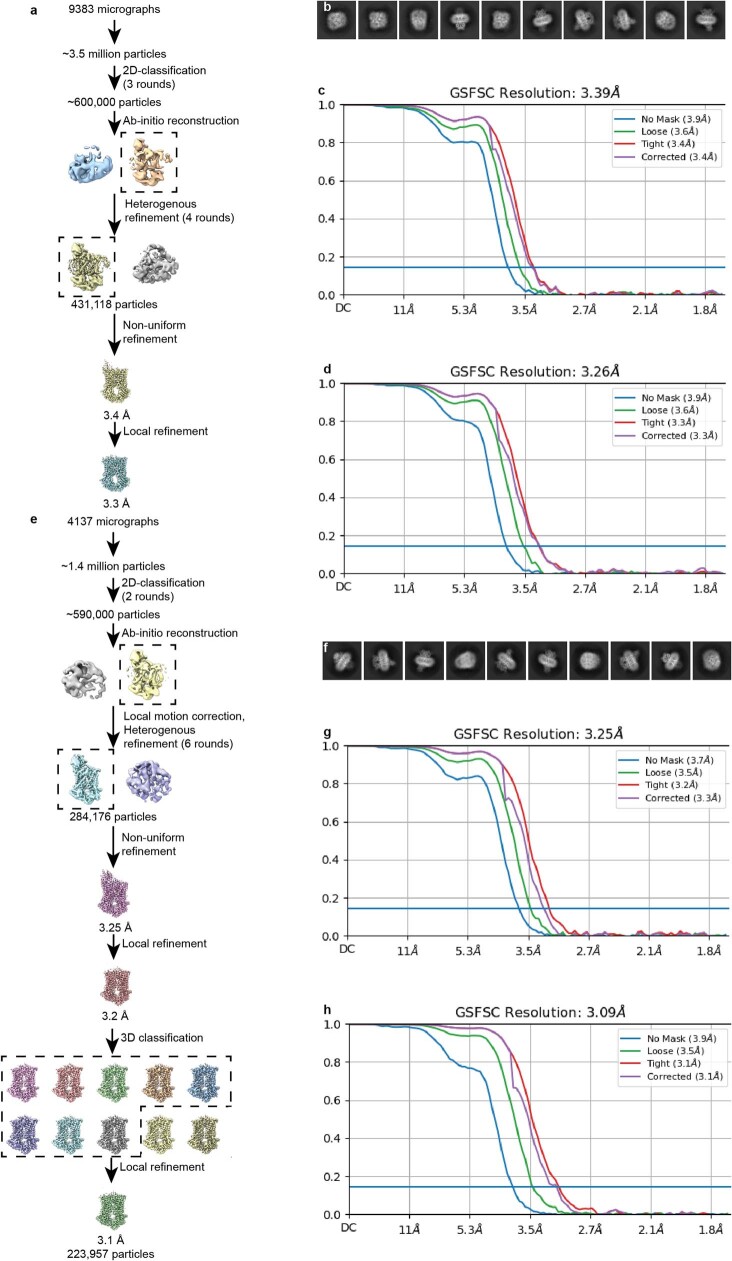
Cryo-EM data processing and analysis for 2- and 3-bound LptB_2_FG. **a**. Scheme of three-dimensional classification and refinement of cryo-EM particle images for **2**-bound LptB_2_FG. The initial models used for 3D classification were generated by ab initio reconstruction in cryoSPARC. **b**. Representative selected two-dimensional class averages of **2**-bound LptB_2_FG. (**c**-**d**) Gold-standard Fourier shell correlation (FSC) curves calculated with different masks in cryoSPARC from the overall (**c**) and locally refined (**d**) structures of **2**-bound LptB_2_FG. The resolution was determined at FSC = 0.143 (horizontal blue line). The final corrected mask gave an overall resolution of 3.25 Å. **e**. Scheme of three-dimensional classification and refinement of cryo-EM particle images for **3**-bound LptB_2_FG. The initial models used for 3D classification were generated by ab initio reconstruction in cryoSPARC. **f**. Representative selected two-dimensional class averages of **3**-bound LptB_2_FG. (**g**-**h**) Gold-standard Fourier shell correlation (FSC) curves calculated with different masks in cryoSPARC from the overall (**f**) and locally refined (**g**) structures of **3**-bound LptB_2_FG. The resolution was determined at FSC = 0.143 (horizontal blue line). The final corrected mask gave an overall resolution of 3.1 Å.

A model that requires C-helix movement for binding to occur would explain an otherwise perplexing observation. Compounds **1**–**3** have comparable cellular potency against wild-type strains and *lptFG* mutations cause similar reductions in susceptibility to all three compounds (Supplementary Table 1). However, when we tested **1**–**3** in a biochemical assay that monitors LPS release from the LptB_2_FGC complex to LptA, we found that compound **3** was 100-fold less effective than **1** and **2** (Fig. 4). To identify the structural basis for these differences in biochemical behaviour, we determined the cryo-EM structures of compounds **2** and **3** bound to LptB_2_FG–LPS. Both **2** and **3** bound in nearly identical positions as compound **1** (Fig. 4a–c and Extended Data Fig. 7). Compounds **1** and **2** contain relatively large substituents—an amino pyridine and a similarly sized benzoate, respectively—that overlap with the predicted position of the LptC TM helix in the gate-occluded state (dashed circles, Fig. 4a,b). These substituents would be predicted to compete more effectively with the LptC TM helix than compound **3**, which contains a smaller chlorine atom at that position (Fig. 4c). If varying degrees of LptC TM helix competition are responsible for the different biochemical activities, **3** should block LPS release as well as **1** and **2** when the LptC TM helix is absent (LptB_2_FGΔTM-LptC). It has previously been demonstrated that ΔTM-LptC is able to support LPS transport even though it lacks the TM helix, allowing us to test this in vitro^19,40^. Consistent with our prediction, compounds **1**–**3** were similarly effective at blocking release from the ΔTM-LptC-containing complex (Fig. 4d). Because **3** is as effective as **1** and **2** in vivo, we conclude that the macrocyclic peptides target a conformational state in vivo in which the C-helix has moved.

## Drug uncouples ATPase activity from LPS transport

**Extended Data Fig. 8:**
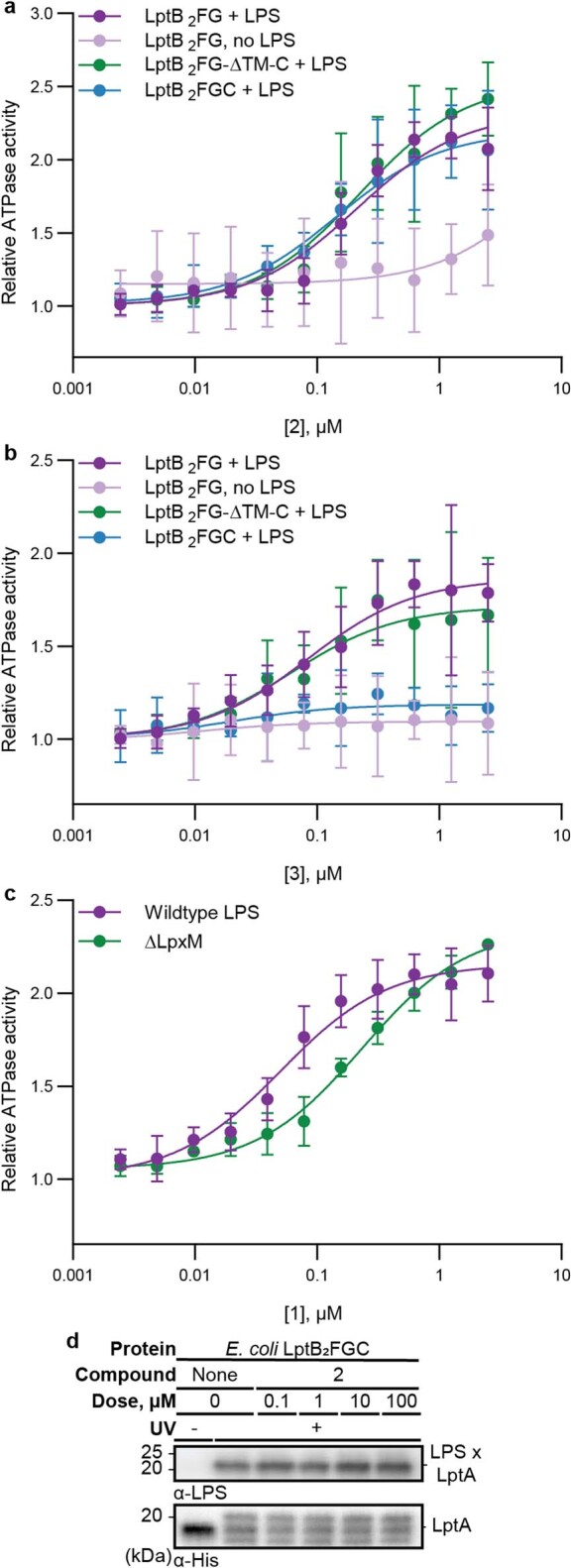
Biochemical determinants of compound binding. a) **2** treatment increases the ATPase activity of LptB_2_FG, LptB_2_FGC and LptB_2_FG-ΔTM-C but only in the presence of LPS. b) **3** treatment increases the ATPase activity of LptB_2_FG, LptB_2_FG-ΔTM-C but only in the presence of LPS. **3** does not affect the ATPase of LptB_2_FGC. c) LPS isolated from a Δ*LpxM* strain renders LptB_2_FGC resistant to **1**. For panels a–c, ATPase rates were measured using liposomes containing the indicated Lpt complex indicated LPS variant and the presented data represent averages and standard deviations of results determined using three different proteoliposomes preparations for each condition variant. Inorganic phosphate release was measured using a molybdate assay. d) **2** does not inhibits LPS transport to LptA by wild-type *E. coli* LptB_2_FGC. Lipopolysaccharide transport from LptB_2_FGC to LptA modified with a photocrosslinkable amino acid (I36*p*BPA) was monitored in the presence of the indicated dose of **2** by exposing the samples to UV light after 60 min of transport, quenching by addition of SDS-loading buffer, PAGE to separate LPS-LptA adducts from LPS and western blotting against LPS. Data shown is representative of experiments conducted in biological triplicate.

LPS loading in the LptB_2_FG transporter has been shown to stimulate ATPase activity^45^. Because our data have shown that these macrocyclic peptides trap LPS in the transporter, we sought to determine if these compounds would increase ATPase activity above the LPS-bound baseline. The addition of compounds **1**–**3** to the LptB_2_FG and LptB_2_FGΔTM-LptC complexes led to a large increase in ATPase activity (Fig. 4e and Extended Data Fig. 8). This activity was dependent on the presence of LPS, supporting the importance of LPS for drug binding to the transporter. The results of the ATPase activity and LPS release assays suggested that the best proxy for in vivo activity of the macrocyclic peptides is the affinity of a compound for LptB_2_FG, in the absence of LptC. Indeed, a scintillation-proximity assay (SPA) showed that radiolabelled **2** bound to LptB_2_FG but not LptB_2_FGC and that binding was dependent on the presence of LPS (Fig. 4f). Consistent with their similar potency in cellular assays, **1**, **2** and **3** had similar abilities to competitively displace H^3^-**2** from LptB_2_FG (*K*_i_ = 35, 35 and 56 nM, respectively). The ATPase and binding assays with complexes lacking LptC or its TM helix therefore recapitulated the in vivo findings that **1**–**3** have comparable activity, again suggesting that the macrocyclic peptides described here target a state in which the LptC TM helix has dissociated from the complex.

We have shown that a new family of macrocyclic peptide antibiotics kills *Acinetobacter* by trapping LPS as this substrate is in transit within the lipopolysaccharide transporter. Because LPS is not required for viability in *Acinetobacter*, a comment is warranted here on the mechanism of cell death. Previous work has shown that many genes involved in LPS biogenesis in *Acinetobacter* are conditionally essential and can only be deleted if initiation of LPS biosynthesis is blocked^46,47^. Toxic accumulation of LPS biosynthesis intermediates results when LPS transport initiates but cannot proceed to completion. We found that *A. baylyi* strains lacking LPS (Δ*lpxC*) can grow in vitro in the presence of very high concentrations of **1** (Supplementary Table 3). Therefore, the drug does not act by depleting LPS from the outer membrane because these cells can live without any LPS in the outer membrane, but through its toxic accumulation within the cell. Although loss of LPS in *Acinetobacter* provides a mechanism to escape drug susceptibility, it significantly decreases both fitness and virulence^46,48,49^. It remains to be seen whether elimination of LPS is a viable strategy to reduce susceptibility to macrocyclic peptide treatment in vivo.

**Fig. 5:**
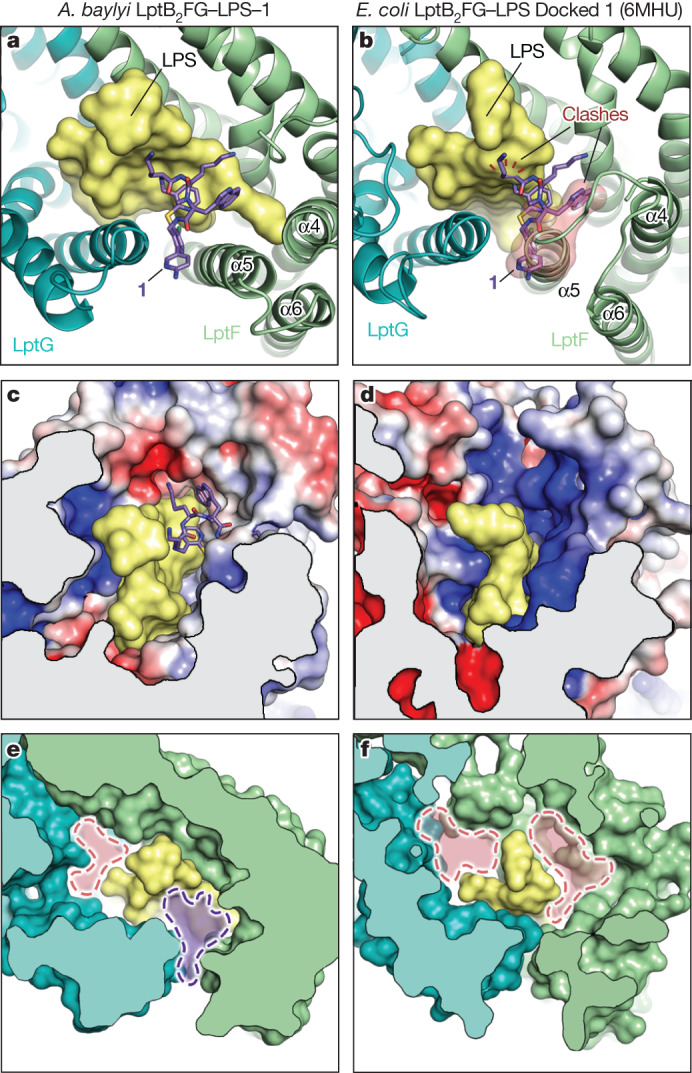
*E. coli* and *Acinetobacter* have distinct druggable pockets at the LPS–LptFG interface. **a**–**f**, Three different representations of the structures of either *Acinetobacter* LptB_2_FG (**a**,**c**,**e**) or *E. coli* LptB_2_FG (b,d,f) in complex with *E. coli* LPS. In the case of *Acinetobacter*, the positioning of **1** is as observed experimentally. In the case of *E. coli*, **1** is placed based on alignment to the *Acinetobacter* LptB_2_FG structure. **a**,**b**, The binding site for macrocyclic peptide **1** that is present in *Acinetobacter* (**a**) is not present in *E. coli* (**b**). As highlighted, the drug has steric clashes with both LPS and helix 5 of *E. coli* LptF. **c**,**d**, The electrostatic surface of *Acinetobacter* (**c**) and *E. coli* (**d**) LptB_2_FG, with negative surfaces shown in red and positive surfaces in blue. Note that the primary amine of the macrocyclic peptides lodge into a negative pocket in *Acinetobacter* LptB_2_FG that does not exist in *E. coli* LptB_2_FG. **e**,**f**, Both *Acinetobacter* and *E. coli* LptB_2_FG have extra cavities formed between LPS and Lpt protein. In this Article, we have validated that drug binding to a composite surface between *Acinetobacter* LptB_2_FG and LPS (purple pocket, **e**) can block LPS transport. Analogous pockets exist in other species (pink, **f**), providing opportunities for future drug design.

The macrocyclic peptide inhibitors are very potent against *Acinetobacter* strains but are inactive against other Gram-negative organisms and we have wondered what lessons our structures and mechanistic experiments hold for understanding this narrow drug susceptibility. We have shown that the ability of macrocyclic peptides to bind *Acinetobacter* LptB_2_FG requires LPS but the structures reveal that these inhibitors contact only the most conserved regions of the LPS lipid A core. Thus, variance of LPS structure alone does not explain the species-selectivity of these drugs (Fig. 1d). The drug pose observed in the ternary structures of *A. baylyi* LptB_2_FG in complex with *E. coli* LPS fully explained the resistance mutations that were isolated in *Acinetobacter*, indicating that contacts with LptFG are critical for drug recognition. Furthermore, results of biochemical transport assays using *E. coli* LPS were consistent with cellular assays that were performed in *Acinetobacter* strains. Therefore, it seems likely that the species-selectivity is due to differences in the Lpt proteins. Homologous bacterial proteins from different genera often have low sequence conservation. Although the sequences of *A. baumannii* and *A. baylyi* LptFG are 82% identical and share almost all the residues (16 of 18) that contact either LPS or the macrocyclic peptide, *E. coli* LptFG proteins are only 25% identical to their *Acinetobacter* counterparts and most of the residues that contact LPS are different (Extended Data Fig. 1). Structures of LptB_2_FG from *E. coli* previously determined in complex with *E. coli* LPS show that LPS occupies a different position in the central cavity of LptFG (refs. ^41,42^) (Fig. 5). Moreover, there are differences in the positions of some of the LptF helices that would result in a clash with the macrocyclic peptides, as well as differences in the electrostatic surface surrounding the LptB_2_FG–LPS binding pockets (Fig. 5c,d). In fact, purified *E. coli* LptB_2_FGC is more than 1,000-fold more resistant to inhibition by the clinical candidate, **2**, than its *Acinetobacter* homologue (Fig. 2c and Extended Data Fig. 8d). The narrow spectrum therefore reflects the differences in the proteins, which affect how they bind LPS. We note that there are other binding pockets surrounding LPS in *E. coli* LptB_2_FG and it may therefore be possible to design analogous inhibitors for this or other Gram-negative pathogens that trap an intermediate LPS-bound state (Fig. 5e,f). More broadly, the mechanism of these molecular glues provides a roadmap for the development of other compounds that bind a transporter and its substrate simultaneously to block lipid transport in prokaryotic and eukaryotic systems^50^.

## Methods

No statistical methods were used to predetermine sample size. The experiments were not randomized and investigators were not blinded to allocation during experiments and outcome assessment.

### SDS–PAGE and immunoblotting

Homemade Tris-HCl 4–20% polyacrylamide gradient gels or 4–20% Mini-PROTEAN TGX precast protein gels (Bio-Rad) were used with Tris-glycine running buffer. The 2× SDS sample loading buffer refers to a mixture containing 125 mM Tris (pH 6.8), 4% (w/v) SDS, 30% (v/v) glycerol, 0.005% bromophenol blue and 5% (v/v) β-mercaptoethanol. SDS–polyacrylamide gel electrophoresis (SDS–PAGE) gels were run for 45–60 min at 200 V. Protein complexes purified for cryo-EM were analysed by SDS–PAGE followed by staining with Coomassie blue (Alfa Aesar) and imaging using the Gel feature of an Azure Biosystems C400 imager. For western blotting, proteins were transferred onto Immun-Blot PVDF membranes (Bio-Rad). Membranes were then blocked using sterile-filtered Casein blocking buffer (Sigma-Aldrich) for 1 h and subsequently incubated with the appropriate antibodies. The following primary antibodies were used: mouse anti-His HRP conjugate (Biolegend, 652504, 1:10,000 dilution) and anti-LPS core mouse monoclonal (Hycult Biotechnology, HM6011, clone WN1 222-5, lot no. 18419M0715-A, 1:5,000 dilution). The following secondary antibodies were used: donkey-anti-rabbit RP conjugate (GE Amersham, NA934-1ML, lot no. 16801031, 1:10,000 dilution), sheep-anti-mouse HRP conjugate (GE AMersham, LNA931V/AH, lot no. 14251045, 1:10,000 dilution). Bands were visualized using ECL Prime western blotting detection reagent (GE Amersham) and an Azure c400 imaging system. Uncropped immunoblots are available in Supplementary Fig. 1.

### Plasmids, strains and oligonucleotides

Genes encoding the LptB, LptC and LptFG were amplified by polymerase chain reaction (PCR) from *Acinetobacter baylyi* ADP1 (ATCC 33305) genomic DNA. *lptB *and *lptFG* PCR products were inserted into pCDFduet by Gibson assembly (New England Biolabs) to generate plasmids analogous to those used for other LptB_2_FG homologues^19^. Similar design was used for the modified plasmid pTRAB-FLAG-LptB-LptFG for purification of the same complex from the native host, which was constructed by combining the gDNA amplicons of the same open reading frames incorporating a linker-less FLAG tag at the N terminus of LptB, a modified trp promoter of *E. coli* and adjacent regions from pTRC99a, a hybrid pBR322WH1266 replicon and a spectinomycin resistance cassette from pCDFduet by Gibson assembly. A linker-less N-terminal His_7_ tag was added to LptB in pCDFduet using NEBuilder HiFi DNA assembly (New England Biolabs). *lptC* PCR products were inserted into pET22/42 with a C-terminal thrombin cleavage site and a His_7_ tag. Oligonucleotide primers were purchased from Eton Biosciences, Genewiz or Integrated DNA Technologies. Plasmids and strains used in this study are reported in Supplementary Tables 4 and 5, respectively. Plasmid sequences are below.

### Construction and use of mutant *A. baylyi* strains

Culture, genetic manipulation and MIC measurements of *A. baylyi* ADP1 were conducted according to previously reported procedures^46,51^. Point mutants were constructed in a two-step procedure following ref. ^52^ with the introduction and excision of the integration cassette at codon 66 of *pepA*, wherein the excising fragment of otherwise wild-type chromosomal DNA sequence from codon 406 of *pepA* to codon 193 of *lptG* bore the desired mutation and the resulting clones were screened by amplicon sequencing from codon 81 of *HolC* to codon 501 of *GpmI*, whereas *lpxM* deletion was achieved following the same procedure except that the integration cassette insertion and excision removed codons 79–279 of *lpxM* to avoid interference with neighbouring and overlapping genes that a larger deletion may risk and replaced codon 78 with an ochre stop codon to prevent a readthrough resulting in an aberrant fusion, with the excising fragment sequence spanning from codon 211 of *sppA* to codon 497 of *ComA* and verified by amplicon sequencing from codon 94 of *MhpC* to codon 327 of *ComA* as well as by absence of a PCR product corresponding to a region spanning codons 79 to 279 of *lpxM* to check for duplications. A deletion of the lon protease was made in the same manner to produce the stain used for expression and purification of LptB_2_FG to mimic the BL21 strain of *E. coli* used in the rest of purifications, for which the region encompassing 72 base pairs (bp) upstream of the *lon* start codon and 1 bp downstream of the *lon* stop codon was excised after being replaced with the same integration cassette, yielding a markerless deletion, with the excising fragment sequence spanning from codon 491 of *ArnT* to codon 40 of *45_DOPA_Dioxygenase* and verified by amplicon sequencing spanning from codon 322 of *ArnT* to codon 221 of *45_DOPA_Dioxygenase* as well as by absence of a PCR product corresponding to a region spanning from codons 328 to 768 of *lon* to check for duplications. Following amplicon confirmation, three validated isolates of each constructed mutant were tested for susceptibility to a panel of antibiotics with known antibiotics with known mechanisms of action as a further validation step to ensure congruence of phenotypes across replicates, which was confirmed in all cases and one of the validated replicates was later used for MIC measurements reported here. In the case of R30A and R55G, no colonies that incorporated these mutations could be isolated, whereas the identical approach readily introduced conservative R30K and R55K substitutions, which resulted in increased antibiotic sensitivity in spite of their mild nature, indicating that impairments caused by substituting dissimilar residues at those positions are not survived.

### MIC determination

MIC determinations were performed by broth microdilution in line with CLSI guidelines (CLSI M07-A11 2018). Bacterial inocula were prepared by diluting overnight liquid cultures in LB. Antibacterial panels containing antibacterial solutions were inoculated with an appropriate volume of inoculum to give a final inoculum of about 5 × 10^5^ c.f.u. ml^−1^ and desired test concentrations of antibacterial agents in standard 96-well plates with 0.1 ml of culture per well. The test plates were incubated for 20–24 h and optical density (OD_600_) was recorded using a plate reader. MIC values corresponded to the lowest compound concentration inhibiting bacterial growth beyond which OD ceased to decrease.

### Purification of LptB_2_FG complexes for cryo-EM

LptB_2_FG complexes were purified as previously described, with slight modifications^20^. Overnight cultures of Bl21(λDE3) *E. coli* containing pCDFduet-His_7_LptB-LptFG or *A. baylyi* containing pTRAB-FLAGLptB-LptFG were diluted 1:100 into LB or terrific broth containing 50 mg l^−1^ of spectinomycin. Cells were grown at 37 °C (or 30 °C for *A. baylyi*) to an OD_600_ of about 0.8. Then 200 µM IPTG and 0.2% glucose (or 500 µM IPTG for *A. baylyi*) were added and cells were allowed to grow for another 2–3 h. Cells were harvested by centrifugation (4,200*g*, 20 min, 4 °C). Cell pellets were flash frozen using liquid nitrogen and stored at −80 °C. All subsequent steps were carried out at 4 °C unless otherwise noted.

Thawed cell pellets were resuspended in lysis buffer (50 mM Tris (pH 7.4), 300 mM NaCl, 1 mM PMSF, 100 μg ml^−1^ of lysozyme, 50 μg ml^−1^ of DNase I, 1 cOmplete Protease Inhibitor Cocktail tablet per 40 ml) homogenized and subjected to passage through an EmulsiFlex-C3 high-pressure cell disruptor three times. The cell lysate was centrifuged (10,000*g*, 10 min) and the supernatant was further centrifuged (100,000*g*, 1 h). The resulting pellets were resuspended and solubilized in solubilization buffer (20 mM Tris (pH 7.4), 300 mM NaCl, 15% glycerol, 5 mM MgCl2, 1% (wt/vol) DDM (Anatrace Maumee), 100 μM PMSF, 2 mM ATP) and rocked at 4 °C for 2 h. (*A. baylyi* cell lysate was immediately subjected to detergent solubilization without the preceding centrifugation steps or ATP addition but 0.35 µM **1** was used to supplement some batches from the solubilization step onward). The mixture was centrifuged (100,000*g*, 30 min), the supernatant was spiked with imidazole to a final concentration of 15 mM and then rocked with Ni-NTA Superflow resin (Qiagen) for 1 h. (*A. baylyi* supernatant was also filtered through a 0.45 µM pore size PVDF Durapore (Millipore-Sigma) membrane and incubated with M2-FLAG agarose resin (Millipore-Sigma) without imidazole supplementation instead of Ni-NTA Superflow resin). The resin was then washed with 2 × 10 column volumes affinity buffer (300 mM NaCl, 20 mM Tris (pH 7.4), 15% glycerol, 0.01% (wt/vol) DDM, 0.04% (wt/vol) GDN (Anatrace Maumee)) containing 20 mM imidazole followed by 2 × 15 column volumes of affinity buffer containing 35 mM imidazole. (*A. baylyi*-derived batches were washed with 3 × 10 column volumes of affinity buffer). Protein was eluted with 2 × 2 column volumes of affinity buffer containing 200 mM imidazole (12.5 column volumes of affinity buffer supplemented with 0.2 mg ml^−^^1^ of FLAG peptide (Genscript) for *A. baylyi*-derived batches) concentrated using a 100 kDa molecular weight cutoff Amicon Ultra centrifugal filter (Millipore) and purified by size-exclusion chromatography on a Superdex 200 increase column in SEC buffer (300 mM NaCl, 20 mM Tris (pH 7.4), 0.02% GDN, 0.25 mM tris(hydroxypropyl)phosphine). Fractions were pooled and concentrated to 7–8 mg ml^−1^ using a 100 kDa molecular weight cutoff Amicon Ultra centrifugal filter. Protein was then prepared for microscopy as described below.

### Purification of LptB_2_FGC complexes for cryo-EM

Purification was conducted largely as described for LptB_2_FG with the following modifications. Expression was conducted using C43(λDE3) *E. coli* containing pCDFduet-LptB-LptFG and pET22/42-LptC-thrombin-His_7_. Cultures were grown in the presence of 50 mg l^−1^ of spectinomycin and 50 mg l^−1^ of carbenicillin. The rest of the expression and purification was conducted identically to the LptB_2_FG purification until the size-exclusion chromatography step. Fractions collected after size-exclusion chromatography were incubated overnight with restriction-grade thrombin (Sigma) to cleave the His tag. The solution was spiked with 8 mM imidazole and the uncleaved protein was removed by passage through Ni-NTA resin and benzamidine Sepharose. Fractions were pooled and concentrated to 7–8 mg ml^−1^ using a 100 kDa molecular weight cutoff Amicon Ultra centrifugal filter. Protein was then prepared for microscopy as described below.

### Electron microscopy data collection

Protein was purified as described above and then incubated on ice with 0.2 mg ml^−1^ of lipopolysaccharides from *E. coli* EH100 (Ra mutant; Sigma-Aldrich) and 0.25 mM drug (if applicable) for 45 min with gentle agitation. For proteins purified out of *Acinetobacter*, *E. coli* lipopolysaccharides were not added. Sample was then applied to glow-discharged C-flat 20 nm holey carbon 1.2 μm hole diameter, 1.3 μm hole spacing, 400-mesh copper grids (Protochips). Grids were blotted for 6.5 s at 4 °C and 100% humidity with the blot force set to 12 and flash frozen by liquid nitrogen-cooled liquid ethane using a Thermo Fisher Scientific Vitrobot Mark IV (Thermo Fisher Scientific). The grid was then loaded onto a Titan Krios G3i electron cryo-microscope (Thermo Fisher) operated at 300 kV accelerating voltage. Image stacks (videos) were recorded on a Gatan Bioquantum K3 Imaging Filter (Gatan), using counting mode and a calibrated magnification of ×105,000 and a pixel size of 0.825 Å, using SerialEM^53^. The slit of the energy filter was set to 20 eV with a defocus range between 1.1 and 2.2 μm. The subframe time was set to allow the collection of 50 subframes per image stack with an electron dose rate of about 1 *e*^−^ per Å^2^ per frame. The total electron dose was about 50 *e*^−^ per Å^2^. The multishot scheme in SerialEM was used for data collection, with settings of nine holes per stage move and two shots per hole. The data collections for all structures were performed in the same manner.

### Image processing and three-dimensional reconstruction

The video frames were motion-corrected and dose-weighted and the contrast transfer function (CTF) parameters were estimated using CryoSPARC Live^54^. Particle picking was carried out using the cryoSPARC blob-picker and junk particles were filtered out by successive rounds of two-dimensional classification in cryoSPARC. Initial models were generated using the ab initio reconstruction in cryoSPARC and then particles were filtered by successive rounds of heterogeneous refinement. After an initial non-uniform refinement job, particles were subject to local motion correction, patch CTF estimation, local CTF refinement and global CTF refinement (fit for beam tilt, beam trefoil and spherical aberration). The particles were then subject to non-uniform refinement to yield the final global reconstruction. Maps were further refined using particle subtraction and local refinement with a mask focused on the TM and nucleotide-binding domains of the transporter. For all maps, we also tried classification without alignment in Relion. At best this only yielded nominal improvements in resolution after reimporting into cryoSPARC and conducting non-uniform refinement when compared to the preclassification maps. 3D classification without alignments in cryoSPARC revealed several possible conformations of the drug within the transporter, as highlighted in Extended Data Fig. 3b,f^55,56^. Maps used for figures were either filtered according to local resolution with *B*-factor sharpening within cryoSPARC or using postprocessing carried out in DeepEMhancer^57^. Structural biology applications used in this project were compiled and configured by SBGrid^58^.

### Model building, refinement and validation

Initial models for LptB, LptF and LptG were generated using SwissModel^59^. The resulting structures were docked into the LptBFG map using Chimera^60^. Cif restraints for *E. coli* lipopolysaccharide were generated using the sketcher tool in CCP4 (ref. ^61^). Cif restraints for *Acinetobacter* lipopolysaccharide were generated using the Grade2 web server from Global Phasing Limited. Cif restraints for the macrocyclic peptides were generated using eLBOW^62^. The coordinates were then refined using Phenix^63,64^. The model was further optimized using ISOLDE^65^, accessed through ChimeraX^66^. Manual model building was carried out in Coot^67^. The final model was visually inspected for general fit to the map and further inspected using MolProbity and the residue-wise local quality estimation DAQ^68,69^. All residues in our models had >0 DAQ scores, except those contained in the helix of LptC. The helix of LptC is modelled as poly-alanine because our maps were not of sufficient quality to allow unambiguous assignment of the helix register. The model validation statistics are summarized in Extended Data Table 1.

### Purification of LptB_2_FG complexes for biochemical reconstitution

LptB_2_FG used for biochemical experiments was purified as described for cryo-EM with the following modifications. The affinity buffer was 300 mM NaCl, 20 mM Tris (pH 7.4), 10% glycerol, 0.015% (wt/vol) DDM. The SEC buffer was 300 mM NaCl, 20 mM Tris (pH 7.4), 5% glycerol, 0.05% DDM, 0.5 mM tris(hydroxypropyl)phosphine.

### Purification of LptB_2_FGC complexes for biochemical reconstitution

LptB_2_FGC used for biochemical experiments was purified as described for cryo-EM with the following modifications. The affinity buffer was 300 mM NaCl, 20 mM Tris (pH 7.4), 10% glycerol, 0.015% (wt/vol) DDM. The SEC buffer was 300 mM NaCl, 20 mM Tris (pH 7.4), 5% glycerol, 0.05% DDM, 0.5 mM tris(hydroxypropyl)phosphine. *E. coli* LptB_2_FGC complexes were purified as described previously^20^.

### Purification of LptA^I36*p*BPA^

LptA^I36*p*BPA^ was purified as described previously^20^. Briefly, Bl21 (λDE3) *E. coli* cells containing pSup-BpaRS-6TRN and pET22b-LptA(I36Am) were grown to an OD_600_ of approximately 0.6 at 37 °C in LB media containing 50 μg ml^−1^ of carbenicillin, 30 μg ml^−1^ of chloramphenicol and 0.8 mM pBPA (BaChem). Cells were then induced with 50 μM IPTG; allowed to grow for 2 h; harvested; resuspended in a mixture containing 50 mM Tris-HCl (pH 7.4), 250 mM sucrose and 3 mM EDTA; incubated on ice for 30 min; and pelleted (6,000*g*, 10 min). The supernatant was supplemented with 1 mM PMSF and 10 mM imidazole and pelleted (100,000*g*, 30 min). The supernatant was incubated with Ni-NTA resin, which was then washed twice (20 column volumes of 20 mM Tris-HCl (pH 8.0), 150 mM NaCl, 10% (vol/vol) glycerol and 20 mM imidazole). LptA was eluted twice (2.5 column volumes of wash buffer supplemented with a further 180 mM imidazole), concentrated using a 10-kDa-cutoff Amicon centrifugal concentrator (Millipore), flash frozen and stored at −80 °C until use.

### Preparation of LptB_2_FG or LptB_2_FGC liposomes

Proteoliposomes were prepared as described previously^20^. Aqueous *E. coli* polar lipid extract (Avanti Polar Lipids) (30 mg ml^−1^) and aqueous LPS from *E. coli* EH100 (Ra mutant; Sigma) (2 mg ml^−1^) were sonicated briefly for homogenization. For experiments testing the effect of LPS structure, we used LPS isolated from either GKM374 (BL21DE3 *eptA*::*catR* *arnA*::*kanR* *eptC*::*gentR*) or TXM418 (BL21DE3 *eptA*::*catR* *arnA*::*FRT* *eptB*::*gentR* *lpxM*::*kanR*) as described previously^36^. A mixture of 20 mM Tris-HCl (pH 8.0), 150 mM NaCl, 7.5 mg ml^−1^ of *E. coli* polar lipids, 0.5 mg ml^−1^ of LPS and 0.25% DDM was prepared and kept on ice for 10 min. Purified LptB_2_FGC or LptB_2_FG was added to a final concentration of 0.86 μM and the mixture was left on ice for 20 min. The mixture was diluted 100-fold with cold 20 mM Tris-HCl (pH 8.0) and 150 mM NaCl and kept on ice for 20 min. The proteoliposomes were pelleted (300,000*g*, 2 h, 4 °C), resuspended in 20 mM Tris-HCl (pH 8.0) and 150 mM NaCl, diluted 100× and centrifuged (300,000*g*, 2 h, 4 °C). The pellets were resuspended in a mixture of 20 mM Tris-HCl (pH 8.0), 150 mM NaCl and 10% glycerol (250 μl per 100 μl of the original predilution solution), homogenized by sonication, flash frozen and stored at −80 °C until use.

### Purification of LptC(ΔTM)

LptC(ΔTM) was purified largely as previously described^19^. Briefly, Bl21 (λDE3) *E. coli* cells containing pET22/42-LptC(ΔTM)-His_7_ were grown to an OD_600_ of approximately 0.6 at 37 °C in LB media containing 50 μg ml^−1^ of carbenicillin. Cells were then induced with 50 μM IPTG; allowed to grow for 2 h; harvested and resuspended in lysis buffer (50 mM Tris pH 7.4, 300 mM NaCl, 0.1 mM EDTA). Lysozyme, DNaseI and PMSF were added to final concentrations of 100 µg ml^−1^, 50 µg ml^−1^ and 1 mM, respectively. Cells were homogenized and subjected to passage through an EmulsiFlex-C3 high-pressure cell disruptor three times. The cell lysate was centrifuged (10,000*g*, 10 min) and the supernatant was further centrifuged (100,000*g*, 1 h). The supernatant was spiked with imidazole to a final concentration of 15 mM and then rocked with Ni-NTA Superflow resin (Qiagen) for 1 h. The resin was then washed with 2 × 10 column volumes of affinity buffer (300 mM NaCl, 20 mM Tris (pH 7.4), 15% glycerol) containing 20 mM imidazole followed by 2 × 15 column volumes of affinity buffer containing 35 mM imidazole. Protein was eluted with 2 × 2 column volumes of affinity buffer containing 200 mM imidazole, concentrated using a 10 kDa molecular weight cutoff Amicon Ultra centrifugal filter (Millipore) and purified by size-exclusion chromatography on a Superdex 200 increase column in SEC buffer (300 mM NaCl, 20 mM Tris (pH 7.4), 5% glycerol). Fractions were pooled and stored at −80 °C.

### LPS release assay

The amounts of release of LPS from proteoliposomes to LptA were measured as previously described^20^. Assays used 60% proteoliposomes (by volume) in a solution containing 50 mM Tris-HCl (pH 8.0), 500 mM NaCl, 10% glycerol and 2 µM LptA^I36*p*BPA^. Reaction mixtures were incubated with drug for 10 min at room temperature, as applicable. Reactions were then initiated by the addition of ATP and MgCl_2_ (final concentrations of 5 mM and 2 mM, respectively) and proceeded at 30 °C. Aliquots (25 µl) were removed from the reaction mixtures and irradiated with ultraviolet (UV) light (365 nm) on ice for 10 min using a B-100AP lamp (Fisher Scientific). Following UV irradiation, 25 µl of 2× SDS–PAGE sample loading buffer was added, samples were boiled for 10 min and proteins were separated using Tris-HCl 4–20% polyacrylamide gradient gels with Tris-glycine running buffer. Immunoblotting was conducted as described above.

### ATPase assay

ATPase assays were done using a modified molybdate method, as previously reported, with slight modifications^20^. Assays used 30% proteoliposomes (by volume) in a mixture containing 50 mM Tris-HCl (pH 8.0), 500 mM NaCl, 10% glycerol and 2 mM MgCl_2_. Proteoliposome-containing reaction mixture was incubated with drug at room temperature for 10 min, as applicable. Reactions were initiated by the addition of ATP to a final concentration of 5 mM and run at 30 °C. Aliquots (5 µl) were taken at 0, 15, 30 and 45 min. Reactions were quenched with an equal volume of 12% SDS. The amounts of P_i_ were determined using a colorimetric method and potassium phosphate was used as a standard^46^. Reagents were obtained from Sigma-Aldrich. After the addition of SDS, a mixture containing 10 µl of 30 mg ml^−1^ of ascorbic acid, 0.5 N HCl, 5 mg ml^−1^ of ammonium molybdate and 6% SDS was added. The samples were incubated at room temperature for 7 min and 15 µl of an aqueous solution containing 20 mg ml^−1^ of sodium citrate tribasic dihydrate, 2 mg ml^−^^1^ of sodium arsenite and 2% (vol/vol) acetic acid was added. The absorbance at 850 nm was measured using a Spectramax Plus 384 (Molecular Devices) after 20 min. Error bars indicate the standard deviations of the average rates measured over three biological replicates.

### Scintillation-proximity assay

The binding of radioligand [^3^H]-RO7223280 to *abl*LptB_2_FG and *abl*LptB_2_FGC was measured by bead-based SPA. All steps were performed in SEC buffer (20 mM Tris pH 7.5, 300 mM NaCl, 5% glycerol, 0.5 mM TCEP, 0.05% DDM ± 10 µM *E.coli* J5 LPS(Rc) TLRGRADE (Enzo Life Sciences)) and at 4 °C unless otherwise indicated. Purified protein was first incubated with Copper PVT HIS-tag beads (Perkin Elmer) for 1.5 h under gentle rotation. Twelve radioligand concentrations were added to the PVT–protein mix and incubated for another 30 min. The mixture was diluted into SPA buffer without or with 500 nM cold RO7223280 to measure total and non-specific binding respectively into an Optiplate-384 microplate (Perkin Elmer). Each well contained 25 ul of total volume, 18 nM protein, 5% dimethylsulfoxide and 6% v/v PVT beads. The SPA plates were sealed (TopSeal, Perkin Elmer) and stored at 4 °C overnight. Before the measurement, plates were mixed on a shaker for 20 min, 750 rpm at room temperature and the seal was thoroughly wiped with antistatic spray to reduce electrostatic events. Scintillation data were recorded with a Topcount NXT C384, in the form of three independent replicates each consisting of three technical triplicates. Specific binding was calculated by subtracting non-specific binding raw counts from total binding raw counts. The dissociation constant *K*_d_ and standard deviation of the three independent replicates are reported and were calculated by the GraphPad Prism ‘One site – specific binding’ tool.

Radioligand displacement experiments were similarly conducted, applying a constant concentration of 25 nM [^3^H]-RO7223280 and 16 concentrations of cold ligands in the presence of 10 µM LPS(Rc). Displacement values were normalized by including nine wells containing no radioligand (defined as 100% competition) and nine wells containing 8 uM radioligand only (defined as 0% competition). The inhibitory constant *K*_i_ was calculated with the ‘One Site – Fit Ki‘ tool using the concentration (25 nM) and *K*_d_ (86 nM) of [^3^H]-RO7223280 as constraints. The Hill coefficient was used as a quality control metric (theoretically, *n*_H_ = 1 for a 1:1 competitive inhibitor) and determined with the ‘[Inhibitor] vs. response -- Variable slope (four parameters)’ tool.

### Reporting summary

Further information on research design is available in the Nature Portfolio Reporting Summary linked to this article.

## Online content

Any methods, additional references, Nature Portfolio reporting summaries, source data, extended data, supplementary information, acknowledgements, peer review information; details of author contributions and competing interests; and statements of data and code availability are available at 10.1038/s41586-023-06799-7.

### Supplementary information

Supplementary Information Supplementary Fig. 1, Tables 1–5 and Methods.

Reporting Summary

## Figures and Tables

**Extended Data Table 1:**
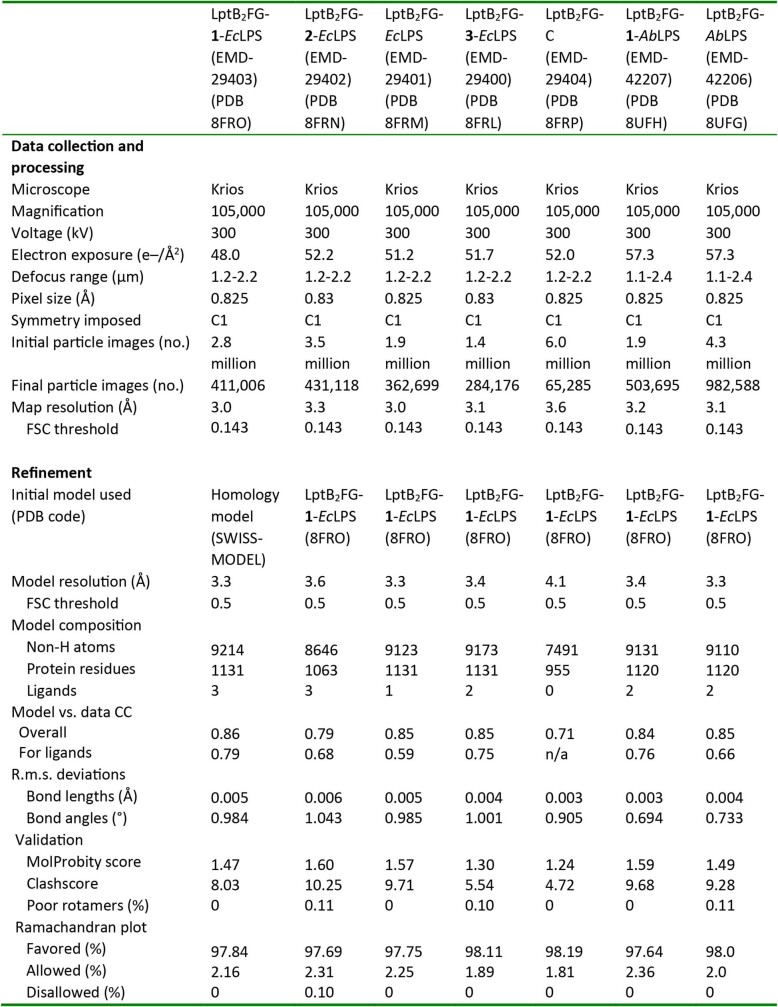
Cryo-EM data collection, refinement and validation statistics

Data processing was conducted using CryoSPARC^54^, as described in detail in the methods and extended data figures. Manual model building was carried out in Coot, further optimized using ISOLDE^65^ and the coordinates were refined using Phenix^63,64,67^.

## Data Availability

The atomic coordinates of *ab*LptB_2_FG-*ec*LPS-**1**, *ab*LptB_2_FG-*ec*LPS, *ab*LptB_2_FG-*ec*LPS-**2**, *ab*LptB_2_FG-*ec*LPS-**3**, *ab*LptB_2_FGC, *ab*LptB_2_FG-*ab*LPS and *ab*LptB_2_FG-*ab*LPS-**1** are deposited at the Protein Data Bank with accession codes 8FRL, 8FRM, 8FRN, 8FRO, 8FRP, 8UFG and 8UFH, respectively. Cryo-EM density maps of *ab*LptB_2_FG-LPS-**1**, *ab*LptB_2_FG-LPS, *ab*LptB_2_FG-*ec*LPS-**2**, *ab*LptB_2_FG-*ec*LPS-**3**, *ab*LptB_2_FGC, *ab*LptB_2_FG-*ab*LPS and *ab*LptB_2_FG-*ab*LPS-**1** are deposited at the Electron Microscopy Data Bank at accession codes EMD-29400, EMD-29401, EMD-29402, EMD-29403, EMD-29404, EMD-42206 and EMD-42207, respectively.
