# Author Correction: A new antibiotic traps lipopolysaccharide in its intermembrane transporter

**DOI:** 10.1038/s41586-024-07035-6

**Published:** 2024-01-10

**Authors:** Karanbir S. Pahil, Morgan S. A. Gilman, Vadim Baidin, Thomas Clairfeuille, Patrizio Mattei, Christoph Bieniossek, Fabian Dey, Dieter Muri, Remo Baettig, Michael Lobritz, Kenneth Bradley, Andrew C. Kruse, Daniel Kahne

**Affiliations:** 1 Department of Chemistry and Chemical Biology, Harvard University, Cambridge, MA USA; 2 Department of Biological Chemistry and Molecular Pharmacology, Harvard Medical School, Boston, MA USA; 3 Departments of Immunology, Infectious Disease and Ophthalmology (I2O), Medicinal Chemistry and Lead Discovery, Roche Pharma Research and Early Development, Roche Innovation Center Basel, Basel, Switzerland

**Keywords:** Cryoelectron microscopy, Mechanism of action, Transporters, Target validation, Antibiotics

Correction to: *Nature* 10.1038/s41586-023-06799-7 Published online 3 January 2024

In the version of this article initially published, there were typos in the Data availability section where PDB accessions 8FRL, 8FRM, 8FRN and 8FRO appeared incorrectly; the codes have been amended in the HTML and PDF versions of the article.

