# Author Correction: A new antibiotic traps lipopolysaccharide in its intermembrane transporter

**DOI:** 10.1038/s41586-024-07645-0

**Published:** 2024-07-11

**Authors:** Karanbir S. Pahil, Morgan S. A. Gilman, Vadim Baidin, Thomas Clairfeuille, Patrizio Mattei, Christoph Bieniossek, Fabian Dey, Dieter Muri, Remo Baettig, Michael Lobritz, Kenneth Bradley, Andrew C. Kruse, Daniel Kahne

**Affiliations:** 1 Department of Chemistry and Chemical Biology, Harvard University, Cambridge, MA USA; 2 Department of Biological Chemistry and Molecular Pharmacology, Harvard Medical School, Boston, MA USA; 3 Departments of Immunology, Infectious Disease and Ophthalmology (I2O), Medicinal Chemistry and Lead Discovery, Roche Pharma Research and Early Development, Roche Innovation Center Basel, Basel, Switzerland

**Keywords:** Cryoelectron microscopy, Mechanism of action, Transporters, Target validation, Antibiotics

Correction to: *Nature* 10.1038/s41586-023-06799-7 Published online 3 January 2024

In the version of the article initially published, three references were missing and have now been added as refs. 7–9: Srinivas, N. et al. Peptidomimetic antibiotics target outer-membrane biogenesis in *Pseudomonas aeruginosa*. *Science* **327**, 1010–1013 (2010); Vetterli, S. U. et al. Thanatin targets the intermembrane protein complex required for lipopolysaccharide transport in *Escherichia coli*. *Sci. Adv*. **4**, eaau2634 (2018) and Moura, E. C. C. M. et al. Thanatin impairs lipopolysaccharide transport complex assembly by targeting LptC–LptA interaction and decreasing LptA stability. *Front. Microbiol*., **11**, 909 (2020). Additionally, in Fig. 1, “R^1^” and “R^2^” appeared as “R_1_” and “R_2_” and in the Fig. 1 caption, the descriptions of panels b and c were swapped. These corrections have now been made to the HTML and PDF versions of the article.

