# Supplementary material for: A new antibiotic traps lipopolysaccharide in its intermembrane transporter
Source: Nature. 2024 Jan 3;625(7995):572–7. doi: 10.1038/s41586-023-06799-7 (PMC10794137; doi:10.1038/s41586-023-06799-7)
Supplement: Supplementary file 1 — Supplementary Fig. 1, Tables 1–5 and Methods. [file 41586_2023_6799_MOESM1_ESM.pdf]

nature portfolio

https://doi.org/10.1038/s41586-023-06799-7

Supplementary information

A new antibiotic traps lipopolysaccharide in its intermembrane transporter

In the format provided by the authors and unedited

Supplementary material for

A new antibiotic traps lipopolysaccharide in its intermembrane transporter

Authors: Karanbir S. Pahil*[1], Morgan S.A. Gilman*[2], Vadim Baidin[1], Thomas Clairfeuille[3], Patrizio Mattei[3], Christoph Bieniossek[3], Fabian Dey[3], Dieter Muri[3], Remo Baettig[3], Michael Lobritz[3], Kenneth Bradley[3], Andrew C. Kruse[†2], Daniel Kahne[†1]

Correspondence to: kahne@chemistry.harvard.edu and Andrew_Kruse@hms.harvard.edu

This PDF file includes:

Supplementary Figure 1

Supplementary Tables 1-5

Supplementary methods – Synthetic methods and sequences of unique plasmids

Supplementary figure 1 Uncropped gels and blots for data shown in main-text and extended data figures. For each figure, the cropped regions are denoted by boxes.

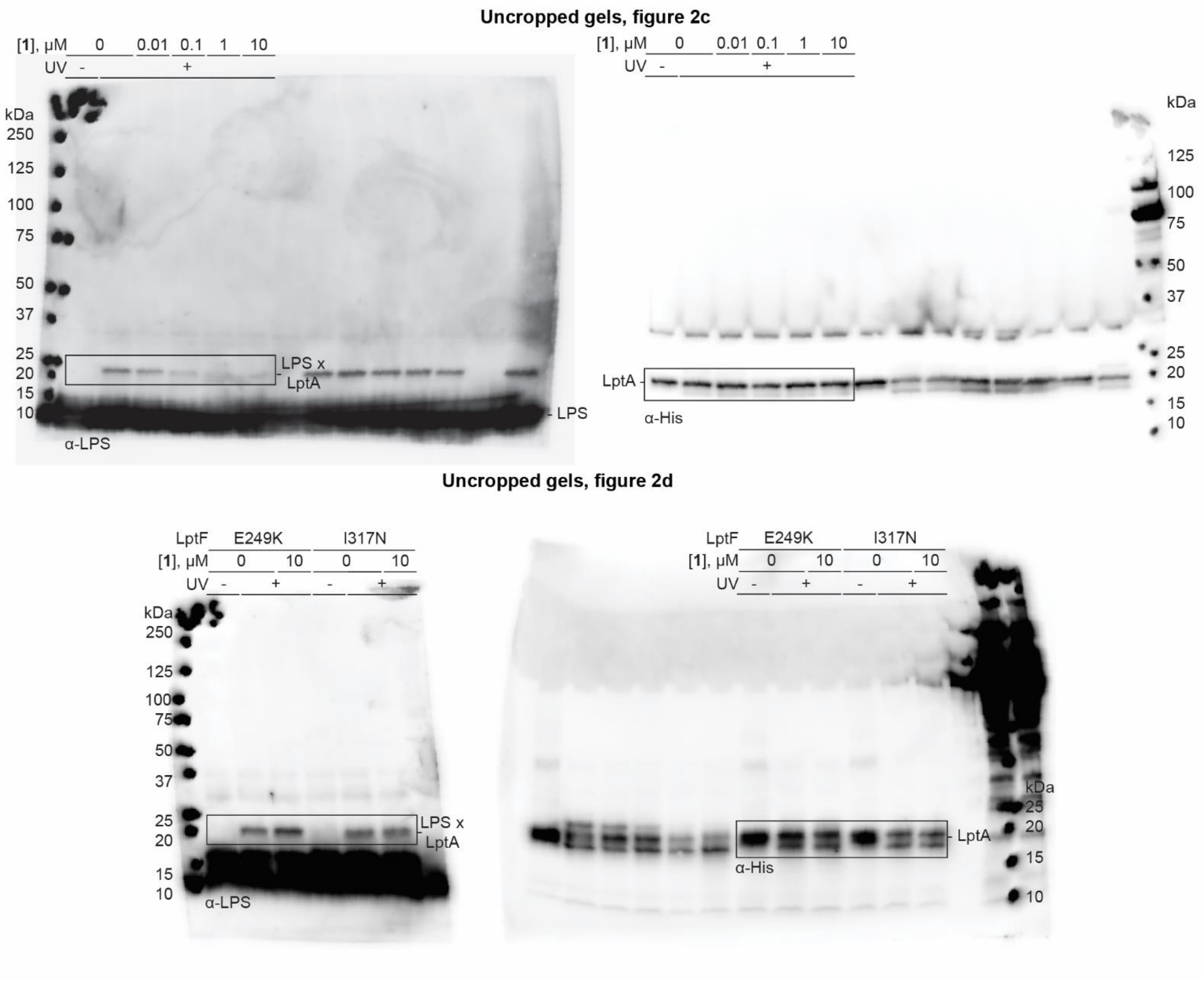

Uncropped gels, figure 3b

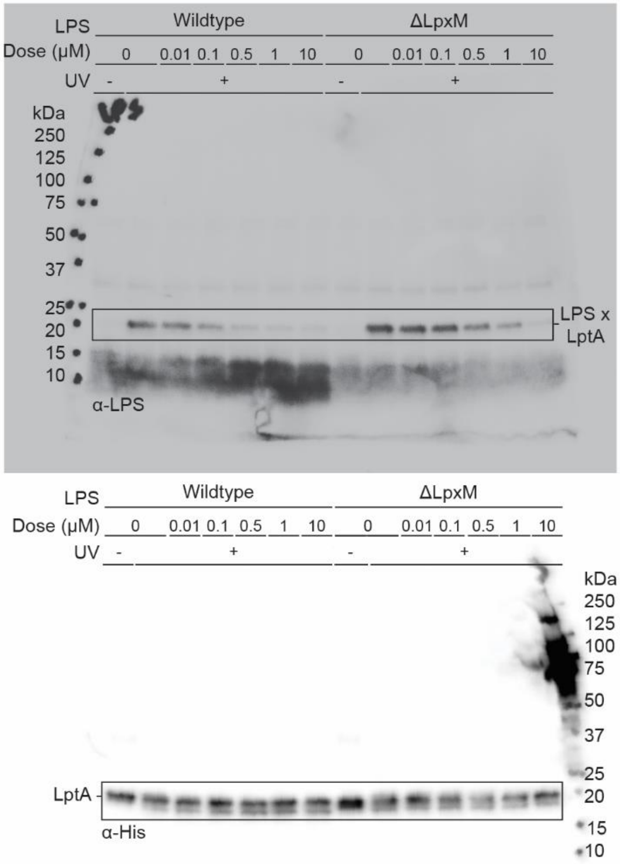

Uncropped gels, figure 4d

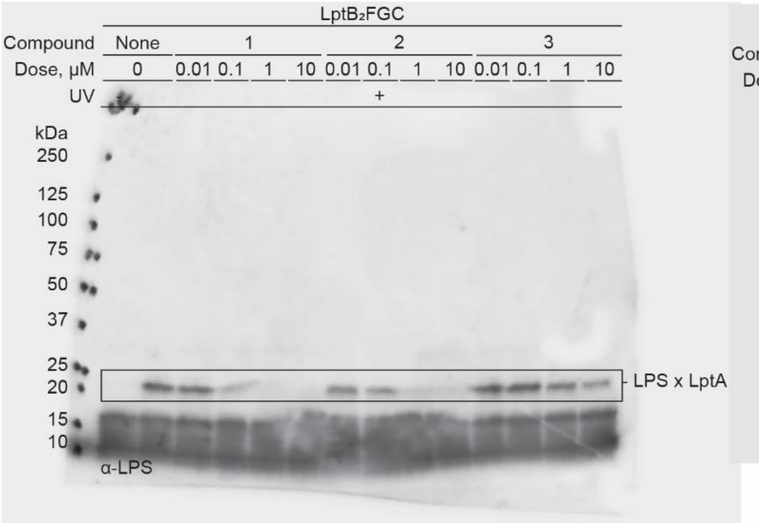

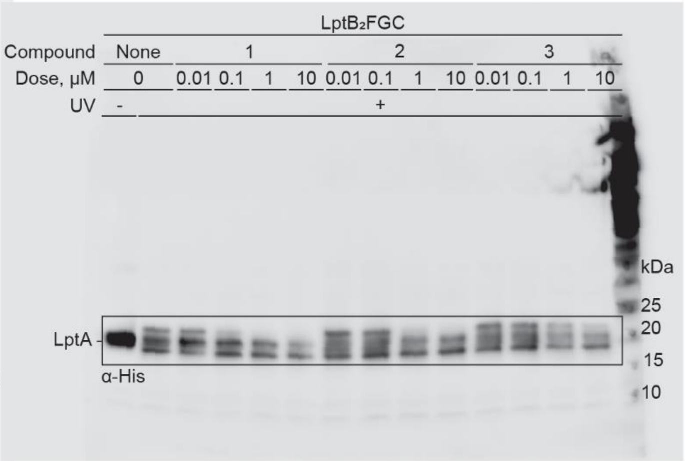

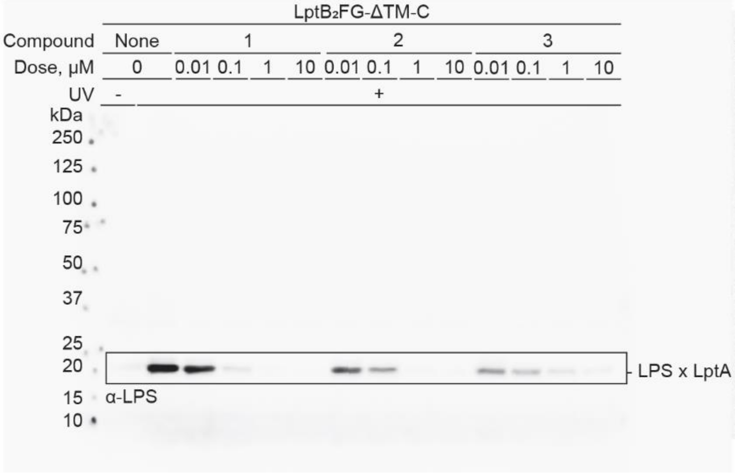

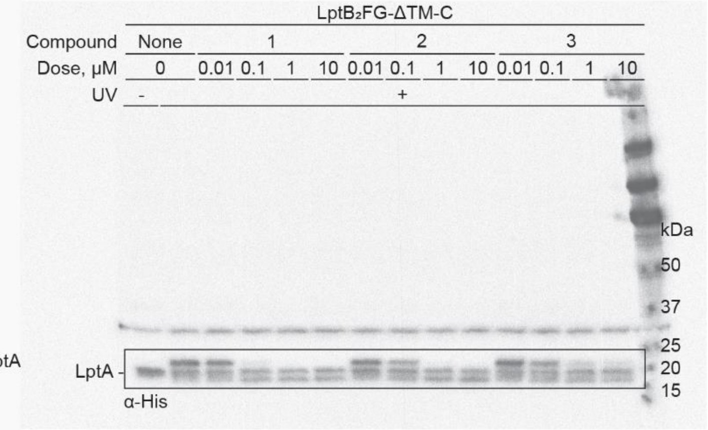

Uncropped gels, Extended data figure 4l

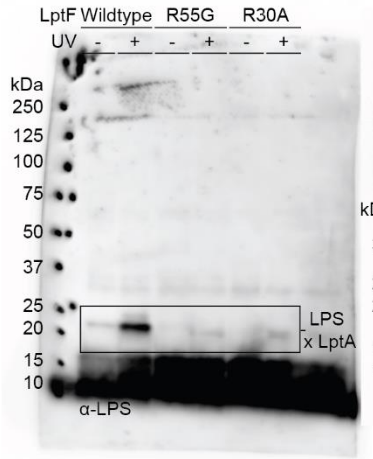

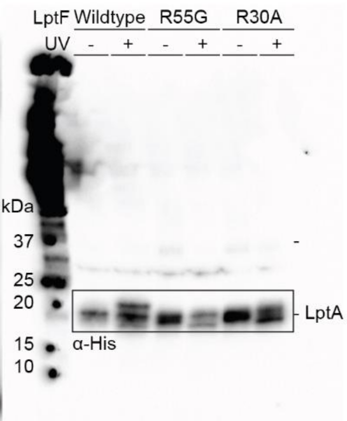

Uncropped gels, Extended data figure 8d

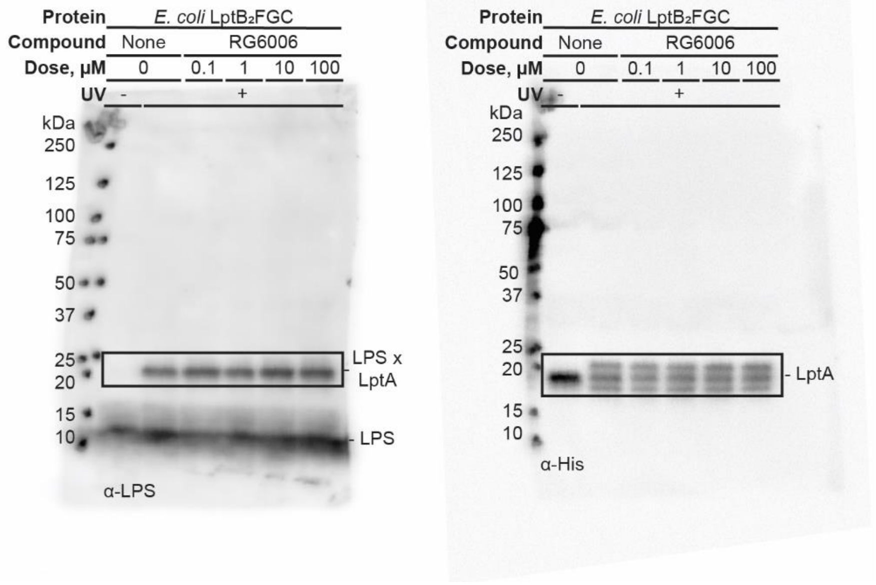

Compound	MIC, µM (µg/mL)		
	**1**	**2**	**3**
Wildtype	0.01 (0.008)	0.06 (0.05)	0.04 (0.03)
LptG(L36Q)	0.07 (0.06)	0.44 (0.35)	0.44 (0.33)
LptF(E58V)	1.2 (0.96)	3.4 (2.7)	14 (10)
LptF(E249K)	1.2 (0.96)	17 (13)	>50 (>37)
LptF(W271R)	0.02 (0.02)	0.1 (0.08)	0.1 (0.07)
LptF(V314L 315del)	0.07 (0.06)	3 (2)	0.24 (0.18)
LptF(I317N)	50 (40)	9 (7)	>50 (>37)
LptF(R320T)	0.04 (0.03)	1.4 (1.1)	0.24 (0.18)

Supplementary table 1. Mutations in LptFG renders *Acinetobacter baylyi* broadly resistant to compounds **1-3**. MIC values of *Acinetobacter baylyi* containing the indicated point mutants in LptF or LptG against compounds **1-3** are shown. MIC values were consistent across three cultures started from individual colonies.

	**MIC, µM (µg/mL)**	
	**Wildtype**	**Δ*LpxM***
Novobiocin	4 (2)	0.6 (0.4)
Coumermycin A1	5 (6)	0.16 (0.18)
Bacitracin	500 (700)	20 (30)
Rifampicin	1.6 (1.3)	0.6 (0.5)
Vancomycin	500 (700)	17 (25)
Triclosan	0.126 (0.036)	0.03 (0.009)

Supplementary table 2. The MICs of wildtype and Δ*lpxM Acinetobacter baylyi* against various antibiotics with diverse mechanisms of actions.

Strain	**MIC, µM (µg/mL)**	
	**Wildtype**	**Δ*LpxC***
**1**	0.01 (0.008)	>50 (>40)

Supplementary table 3. Knockout of LpxC renders *Acinetobactr baylyi* resistant to **1**. MIC values of wildtype and ΔLpxC *Acinetobacter baylyi* compound **1** are shown. MICs values were consistent across three cultures started from individual colonies.

Strains	Genotype	Source
NovaBlue	*endA1 hsd*R17 (r_K^-, m_K^+) *sup*E44 *thi*-1 *rec*A1 *gyr*A96 *rel*A1 *lac* F′[*pro*A$^+$ B$^+$ *lac*I^q ZΔM15::Tn*10*]	Novagen
BL21(DE3)	*fhuA2 [lon] ompT gal (λ DE3) [dcm] ΔhsdS*	Novagen
C43(DE3)	*F – ompT hsdSB (rB- mB-) gal dcm (DE3)*	Lucigen
A. baylyi	ADP1	ATCC
A. baylyi ΔlpxC::kan	Single deletion mutant in lpxC in the background of WT ADP1	[33]

Supplementary table 4. Bacterial strains used in this work

Plasmid	Description	Source
pET22/42-LptC-thrombin-His_7	pET22/42 encoding full-length *Acinetobacter baylyi* LptC with a C-terminal cleavable His_7 tag	Companion paper
pET22/42-LptCΔTM-thrombin-His_7	pET22/42 encoding *Acinetobacter baylyi* LptC residues 24-182 with a C-terminal cleavable His_7 tag	This work
pCDFduet-LptB-LptFG	pCDFduet encoding full-length *Acinetobacter baylyi* LptB at the first site and *Acinetobacter baylyi* LptFG at the second site.	Companion paper
pCDFduet-HisLptB-LptFG	pCDFduet encoding full-length *Acinetobacter baylyi* LptB with an N-terminal His_7 tag at the first site and *Acinetobacter baylyi* LptFG at the second site.	This work
pTRAB-FLAG-LptB-LptFG	pBR322WH1266 spectinomycinR plasmid encoding full-length *Acinetobacter baylyi* LptB with an N-terminal FLAG tag and LptFG under a TRC promoter.	This work
pCDFduet-LptB-$LptF^{E249K}G$	pCDFduet-LptB-LptFG with LptF(E249K)	Companion paper
pCDFduet-LptB-$LptF^{I317N}G$	pCDFduet-LptB-LptFG with LptF(I317N)	Companion paper
pCDFduet-LptB-$LptF^{E249K,I317N}G$	pCDFduet-LptB-LptFG with LptF(E249K, I317N)	Companion paper
pCDFduet-HisLptB-$LptF^{R30A}G$	pCDFduet-HisLptB-LptFG with LptF(R30A)	This work
pCDFduet-HisLptB-$LptF^{R55G}G$	pCDFduet-HisLptB-LptFG with LptF(R55G)	This work
pTRAB-FLAG-LptB-LptFG	The gDNA amplicons of the LptBFG from *Acinetobacter baylyi* incorporating a linker-less FLAG tag at the N-terminus of LptB, a modified trp promoter of *E. coli* and adjacent regions from pTRC99a, a hybrid pBR322WH1266 replicon, and a spectinomycin resistance cassette from pCDFduet	This work
pET22b-LptA(I36Am)	pET22b encoding LptA(I36Am) with a His tag.	[14]

Supplementary table 5. Bacterial plasmids used in this work

Supplementary methods

Synthetic methods

Synthesis of (11S,14S,17S)-14-(4-aminobutyl)-11-(3-aminopropyl)-22-(6-amino-3-pyridyl)-25-chloro-17-(1H-indol-3-ylmethyl)-16-methyl-2-thia-4,10,13,16,19-pentazatricyclo[19.4.0.03,8]pentacosa -1(25),3(8),4,6,21,23-hexaene-12,15,18-trione (1):

a) tert-Butyl 3-[[(11S,14S,17S)-22-(6-amino-3-pyridyl)-14-[4-(tert-butoxycarbonylamino)butyl]-11-[3-(tert-butoxycarbonylamino)propyl]-25-chloro-16-methyl-12,15,18-trioxo-2-thia-4,10,13, 16,19-pentazatricyclo[19.4.0.0^{3,8}]pentacosa-1(25),3(8),4,6,21, 23-hexaen-17-yl]methyl] indole-1-carboxylate

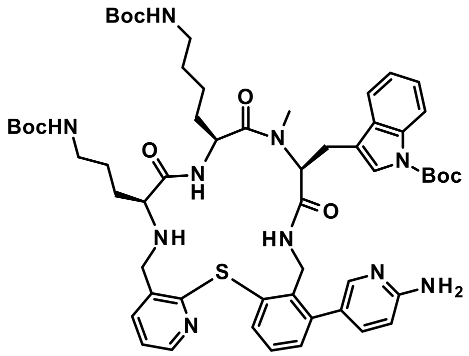

To a solution of tert-butyl 3-(((7S,10S,13S)-17-bromo-10-(4-((tert-butoxycarbonyl) amino) butyl)-7-(3-((tert-butoxycarbonyl)amino)propyl)-20-chloro-12-methyl-8,11,14-trioxo-5,6,7,8, 9,10,11,12,13,14,15,16-dodecahydrobenzo[b]pyrido[3,2-p][1]thia[5,8,11,14]tetraazacyclo heptadecin-13-yl)methyl)-1H-indole-1-carboxylate (190 mg, 0.18 mmol, 1.0 eq) and 6-aminopyridine-3-boronic acid (48.3 mg, 0.35 mmol, 2.0 eq) in 1,4-dioxane (2 mL) and water (0.2 mL) was added sodium carbonate (46.4 mg, 0.44 mmol, 2.5 eq) and cataCXium-A-PdG3 (CAS-RN [1651823-59-4]; 25.5 mg, 0.04 mmol, 0.2 eq) in glove box in a microwave tube. After addition, the mixture was stirred at 100 °C for 16 h. The mixture was then concentrated to give a residue, which was purified by preparative thin-layer chromatography (petroleum ether / ethyl acetate 1:3) to produce the title compound (75 mg, 39%). White solid, MS: 1097.5 $[M+H]^+$.

*b) (11S,14S,17S)-14-(4-Aminobutyl)-11-(3-aminopropyl)-22-(6-amino-3-pyridyl)-25-chloro-17-(1H-indol-3-ylmethyl)-16-methyl-2-thia-4,10,13,16,19-pentazatricyclo[19.4.0.0^{3,8}]pentacosa -1(25),3(8),4,6,21,23-hexaene-12,15,18-trione (**1**)*

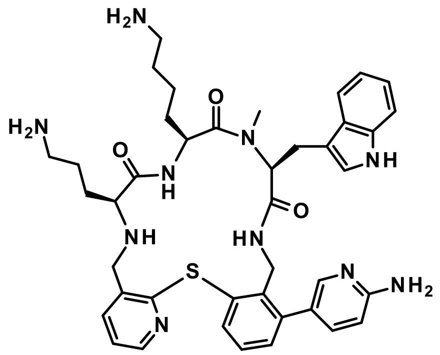

To a solution of tert-butyl 3-[[(11S,14S,17S)-22-(6-amino-3-pyridyl)-14-[4-(tert-butoxycarbonylamino)butyl]-11-[3-(tert-butoxycarbonylamino)propyl]-25-chloro-16-methyl-12,15,18-trioxo-2-thia-4,10,13, 16,19-pentazatricyclo[19.4.0.0^{3,8}]pentacosa-1(25),3(8),4,6,21, 23-hexaen-17-yl]methyl] indole-1-carboxylate (65.0 mg, 0.06 mmol, 1.0 eq) in 1,4-Dioxane (1 mL) was added hydrogen chloride solution (4 M in 1,4-dioxane, 3.25 mL, 13.0 mmol). After addition, the mixture was stirred at 25 °C for 2 h to give a white suspension. The solid was collected by filtration and washed with 1,4-dioxane (2 mL). The filter cake was collected and dissolved in water (2 mL), then the solution was

purified by preparative HPLC (column: YMC Triart C18 150*25mm particle size: 5 µm; mobile phase: A: water (0.05 % conc. HCl in water (v/v)), B: MeCN; gradient: B%: 10%-40%, 8.5 min; flow rate: 29 mL/min) and freeze drying to give the title compound as the dihydrochloride salt (25 mg, 48%).

Yellow solid, MS: 797.5 $[M+H]^+$. ^1H NMR (400 MHz, D_2O) δ ppm 8.77 (s, 1H), 8.38 (br s, 1H), 7.96 (d, J = 9.3 Hz, 1H), 7.91 (s, 1H), 7.72 (d, J = 6.7 Hz, 1H), 7.68 (d, J = 8.2 Hz, 1H), 7.58 (d, J = 7.1 Hz, 1H), 7.45 (d, J = 8.2 Hz, 2H), 7.31-7.18 (m, 2H), 7.10 (d, J = 9.0 Hz, 1H), 7.07-6.97 (m, 2H), 4.68-4.51 (m, 3H), 4.37-4.23 (m, 1H), 4.14-3.91 (m, 2H), 3.22-3.11 (m, 1H), 3.04-2.85 (m, 3H), 2.77-2.55 (m, 5H), 2.42-2.23 (m, 1H), 1.97-1.84 (m, 1H), 1.83-1.71 (m, 1H), 1.68-1.50 (m, 2H), 1.10-0.79 (m, 2H), 0.73-0.42 (m, 2H), -0.55--1.08 (m, 2H).

Synthesis of [^{3}H]-RG6006

a) (4-Methoxycarbonyl-3,5-ditritio-phenyl)boronic acid

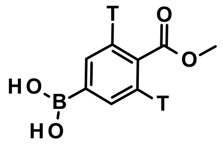

In a 2 ml tritiation flask, methyl 2,6-dichloro-4(4,4,5,5-tetramethyl-1,2,3-dioxoborolan-2-yl)benzoate (10.0 mg, 30.2 µmol, 1.00 eq.) Pd/C (10%) (9.65 mg, 9.06 µmol, 0.30 eq.), and diisopropylethylamine (14.8 mg, 20.1 µL, 115 µmol, 3.80 eq.) were suspended in dimethylformamide (0.8 mL). The flask was attached to the tritium manifold (RC-TRITEC) and degassed by 3 freeze-pump-thaws. Tritium gas was introduced, and the black suspension was vigorously stirred for 20 hours under an atmosphere of tritium at an initial pressure of 600 mbar. The solution was cooled by liquid nitrogen and the excess tritium gas in the reaction vessel was reabsorbed on a uranium-trap for waste-tritium. The solvent was lyophilized off and labile tritium was removed by lyophilization with methanol (3 x 1.0 mL). The remaining black residue was suspended in ethanol (15 mL) and filtered over a 17 mm Titan HPLC filter (0.45 µm, PTFE. The crude product was concentrated, deprotected and purified by preparative HPLC (Gilson PLC2050, Sunfire C18 OBD, 5 µm, 10 x 250 mm, detection: UV at 254 nm) using 1% trifluoroacetic acid in water[A] acetonitrile [B] and 5% acetonitrile in water [C] as eluent (gradient: 10% [A], 20% [B], 70% [C] to 10 %[A], 90 %[B] in 12 min, then back to initial conditions for 3 min at a flow rate of 6 mL/min). The pure fractions were lyophilized and dissolved in ethanol (20 mL). 19.7 GBq (533 mCi) were obtained of the title compound with a radiochemical purity of 98% and a molar activity of 1150 GBq/mmol (31 Ci/mmol), determined by MS spectrometry. The compound was stored as an ethanolic solution. MS m/z: 225.2 $[M+HCOO]^-$ (23%), 227.2 $[M(^3H)+HCOO]^-$ (46%), 229.1 $[M(^3H_2)+HCOO]^-$ (31%). The identity was confirmed by co-injection with a cold reference sample.

b) 4-(11S,14S,17S)-14-[4-(tert-Butoxycarbonylamino)butyl]-11-[3-(tert-butoxycarbonylamino)propyl]-17-(1H-indol-3-ylmethyl)-16-methyl-12,15,18-trioxo-2-thia-4,10,13,16,19-pentazatricyclo[19.4.0.0^{3,8}]pentacosa-1(21),3,5,7,22,24-hexaen-22-yl]-2,6-ditritio-benzoic acid

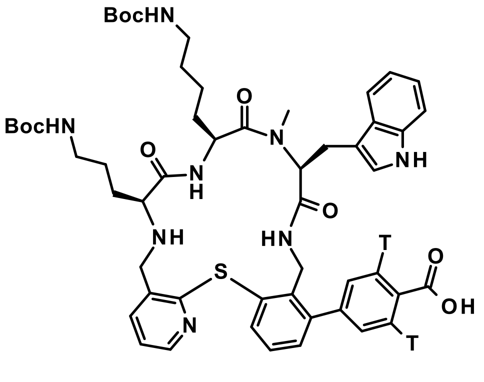

In a 1-mL reaction-vial (4-methoxycarbonyl-3,5-ditritio-phenyl)boronic acid (9.88 GBq (267 mCi), 1.58 mg, 8.6 µmol, 1.00 eq.), *tert*-butyl 3-(((*7R*,*10R*,*13R*)-17-bromo-10-(4-((*tert*-butoxycarbonyl)amino)butyl)-7-(3-((*tert*-

butoxycarbonyl)amino)propyl)-12-methyl-8,11,14-trioxo-5,6,7,8,9,10,11,12,13,14,15,16-dodecahydrobenzo[b]pyrido[3,2-p][1]thia[5,8,11,14]tetraazacycloheptadecin-13-yl)methyl)-1*H*-indole-1-carboxylate (9.03 mg, 2.15 µmol, 1.00 eq.) and CPhos Pd G3 (1.73 mg, 2.15 µmol, 0.25 eq.) were added to toluene (0.4 mL). Then a solution of potassium phosphate hydrate (3.11 mg, 13.5 µmol, 1.57 eq.) in water (79.1 µL) were added. Upon heating to 85 °C, the reaction mixture turned yellow and was stirred at that temperature for 18 h. The yellow emulsion was cooled down to room temperature, and toluene was removed under a stream of argon. To the remaining solid was added methanol (0.3 mL), water (47.5 µL), and aqueous sodium hydroxide solution (32%) (8.0 µL, 86 µmol, 10 eq.). The yellow reaction mixture was heated to 55 °C, stirred for 4 hours, and cooled down to room temperature. The mixture was added to dichloromethane (20 mL) and water (20 mL). Under strong stirring aqueous citric acid (5%) (0.8 µL) was slowly added to reach pH 5 (checked by pH paper). The layers were separated and the aqueous layer was washed with dichloromethane (20 mL). The combined organic phases were dried over sodium sulfate, filtered, and concentrated under reduced pressure at 40 °C. The product could not be separated by extraction, therefore, also the aqueous layer was concentrated. Both layers were dissolved in acetonitrile:water:trifluoroacetic acid 50:50:1 and purified by preparative HPLC (Gilson PLC2050, Sunfire C18 OBD, 5 µm, 10 x 250 mm, detection: UV at 254 nm, using 1% trifluoroacetic acid in water[A] acetonitrile [B] and 5% acetonitrile in water [C] as eluent (gradient: 20% [A], 30% [B], 50% [C] to 20 %[A], 80 %[B] in 12 min, hold for 2 min, then back to initial conditions for 3 min at a flow rate of 6 mL/min). The pure fraction was lyophilized and dissolved in ethanol (15 mL). 1.80 GBq (48.7 mCi) (18% RCY) of the desired compound was obtained with a radiochemical purity of 99% and a molar activity of 1147 GBq/mmol (31 Ci/mmol), as determined by MS spectrometry. MS m/z: 991.5 $[M(^3H)+H]^+$ (24%), 993.5 $[M(^3H)+H]^+$ (44%), 995.5 $[M(^3H)_2+H]^+$ (32%). The identity was confirmed by co-injection with a cold reference sample.

*c) 4-[(11S,14S,17S)-14-(4-Aminobutyl)-11-(3-aminopropyl)-17-(1H-indol-3-ylmethyl)-16-methyl-12,15,18-trioxo-2-thia-4,10,13,16,19-pentazatricyclo[19.4.0.0^{3,8}]pentacosa-1(25),3(8),4,6,21,23-hexaen-22-yl]-2,6-ditritio-benzoic acid (**[^{3}H]-RG6006**)*

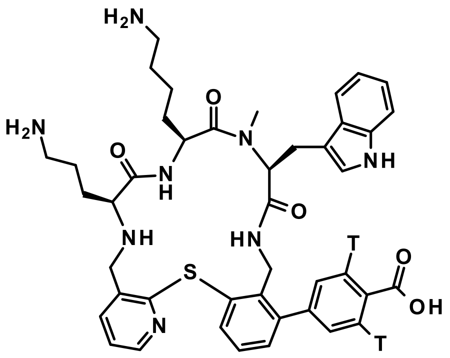

4-(11*S*,14*S*,17*S*)-14-[4-(*tert*-Butoxycarbonylamino)butyl]-11-[3-(*tert*-butoxycarbonylamino)propyl]-17-(1*H*-indol-3-ylmethyl)-16-methyl-12,15,18-trioxo-2-thia-4,10,13,16,19-pentazatricyclo[19.4.0.03,8]pentacosa-1(21),3,5,7,22,24-hexaen-22-yl]-2,6-ditritio-benzoic acid (1590 MBq (43.0 mCi), 1.39 µmol, 1.00 eq.) was dissolved in 1,4-dioxane (5.5 mL) and HCl in 1,4-dioxane (4 Molar) (347 µL, 1.39 mmol, 1000 eq.) were added, and the clear solution was stirred at 40 °C for 5 h. The light turbid suspension was evaporated and dried under vacuum. The crude product was dissolved in water:acetonitrile:TFA 50:50:1 and purified by preparative HPLC (Gilson PLC2050, Sunfire C18 OBD, 5 µm, 10 x 250 mm, detection: UV at 254 nm, using 1% trifluoroacetic acid in water[A] acetonitrile [B] and 5% acetonitrile in water [C] as eluent (start at 15% [A], 10% [B], 75% [C] for 1 min, then a gradient to 15 %[A], 50 %[B], 35% [C] in 14 min, to 15 %[A], 75 %[B], 10% [C] in 3 min, hold for 2 min, then back to initial conditions for 5 min at a flow rate of 6 mL/min). The product containing fraction was lyophilized and dissolved in ethanol/water (10 mL). 407 MBq (11.0 mCi) (26% RCY) of the desired compound as TFA salt was obtained with a radiochemical purity of 99% and a molar activity of 1151 GBq/mmol (31.1 Ci/mmol), as determined by MS spectrometry. MS m/z: 791.3 $[M(^3H)+H]^+$ (23%), 793.3 $[M(^3H)+H]^+$ (45%), 795.4 $[M(^3H)_2+H]^+$ (32%). The identity was confirmed by co-injection with a cold reference sample.

Sequence of unique plasmids

Sequence of the plasmid pET22/42-LptC-thrombin-His$_7$

agatctcgatcccgcgaaattaatacgactcactataggggaattgtgagcggataacaattcccctctagaaagaattttgtttaactttaagaaggagatatacat**ATGG**
ATACCAGAGCTCTTTATATTGCTGCTGTTGTAATCGCAAGTATTAGTGGCGGTTACTATTACTATAGTGGTAAGGCAAAAAAACTT
GATGTCGACTCGGCTCGAAATATGACTTATTCGGCCGAGGGAGTAAATCTTACACAAACGGATGATCAAGGGCATTTGTATGTAC
GTGCAACAGTAGATCGTCTAGAGCAAAATATGCAAAATCAGACATCTAAGTTGGAAAATCTGAATGCATCCACATATAAAGCTGG
CAATGTCGATTCAACATTTTTTTCTAAATATGCCTATGGTTACAATGACAATGAAAAGGTTGTCCTTTCAGATCAAGTTGTTGCAACT
AAACTCTCTGATCATGGAAAAATGCAATTTCAAACTGATGAGTTAACCACCTATCCTCCGAAAAAAATGATTGAGACGACTCATCA
AGTCAATGTGGATTCTCCACAGTCATCTTTTATCAGTCAGGGATTAAAAGCAGATTTAAACACTGGTCAATACGAGTTTTTAATAT
TCGAGGAAAGTATGCACCGAATTCTCTGGTACCGCGCGGCAGCCACCACCACCACCACCACTAAttgattaatacctaggctgctaaaca
aagcccgaaaggaagctgagttggctgctgccaccgctgagcaataactagcataaccccttggggcctctaaacgggtcttgaggggttttttgctgaaaggaggaactata
tccggattggcgaatgggacgcgccctgtagcggcgcattaagcgcggcgggtgtggtggttacgcgcagcgtgaccgctacacttgccagcgccctagcgcccgctcctttcg
ctttcttcccttcctttctcgccacgttcgccggctttccccgtcaagctctaaatcgggggctccctttagggttccgatttagtgctttacggcacctcgaccccaaaaaacttga
ttagggtgatggttcacgtagtgggccatcgccctgatagacggtttttcgccctttgacgttggagtccacgttctttaatagtggactcttgttccaaactggaacaacactca
accctatctcggtctattcttttgatttataagggattttgccgatttcggcctattggttaaaaaatgagctgatttaacaaaaatttaacgcgaattttaacaaaatattaacgc
ttacaatttaggtggcacttttcggggaaatgtgcgcggaacccctatttgtttatttttctaaatacattcaaatatgtatccgctcatgagacaataaccctgataaatgcttca
ataatattgaaaaaggaagagtatgagtattcaacatttccgtgtcgcccttattcccttttttgcggcattttgccttcctgtttttgctcacccagaaacgctggtgaaagtaaa
agatgctgaagatcagttgggtgcacgagtgggttacatcgaactggatctcaacagcggtaagatccttgagagttttcgccccgaagaacgttttccaatgatgagcactttt
aaagttctgctatgtggcgcggtattatcccgtattgacgccgggcaagagcaactcggtcgccgcatacactattctcagaatgacttggttgagtactcaccagtcacagaa
aagcatcttacggatggcatgacagtaagagaattatgcagtgctgccataaccatgagtgataacactgcggccaacttacttctgacaacgatcggaggaccgaaggagc
taaccgcttttttgcacaacatgggggatcatgtaactcgccttgatcgttgggaaccggagctgaatgaagccataccaaacgacgagcgtgacaccacgatgcctgcagca
atggcaacaacgttgcgcaaactattaactggcgaactacttactctagcttcccggcaacaattaatagactggatggaggcggataaagttgcaggaccacttctgcgctcg
gcccttccggctggctggtttattgctgataaatctggagccggtgagcgtgggtctcgcggtatcattgcagcactggggccagatggtaagccctcccgtatcgtagttatcta
cacgacggggagtcaggcaactatggatgaacgaaatagacagatcgctgagataggtgcctcactgattaagcattggtaactgtcagaccaagtttactcatatatacttt
agattgatttaaaacttcatttttaatttaaaaggatctaggtgaagatcctttttgataatctcatgaccaaaatcccttaacgtgagttttcgttccactgagcgtcagaccccg
tagaaaagatcaaaggatcttcttgagatcctttttttctgcgcgtaatctgctgcttgcaaacaaaaaaaccaccgctaccagcggtggtttgtttgccggatcaagagctacca
actctttttccgaaggtaactggcttcagcagagcgcagataccaaatactgtccttctagtgtagccgtagttaggccaccacttcaagaactctgtagcaccgcctacatacc
tcgctctgctaatcctgttaccagtggctgctgccagtggcgataagtcgtgtcttaccgggttggactcaagacgatagttaccggataaggcgcagcggtcgggctgaacgg
ggggttcgtgcacacagcccagcttggagcgaacgacctacaccgaactgagatacctacagcgtgagctatgagaaagcgccacgcttcccgaagggagaaaggcggac
aggtatccggtaagcggcagggtcggaacaggagagcgcacgagggagcttccagggggaaacgcctggtatctttatagtcctgtcgggtttcgccacctctgacttgagcg
tcgatttttgtgatgctcgtcaggggggcggagcctatggaaaaacgccagcaacgcggcctttttacggttcctggccttttgctggccttttgctcacatgttctttcctgcgtta
tcccctgattctgtggataaccgtattaccgcctttgagtgagctgataccgctcgccgcagccgaacgaccgagcgcagcgagtcagtgagcgaggaagcggaagagcgcc
tgatgcggtattttctccttacgcatctgtgcggtatttcacaccgcaatggtgcactctcagtacaatctgctctgatgccgcatagttaagccagtatacactccgctatcgcta
cgtgactgggtcatggctgcgccccgacacccgccaacacccgctgacgcgccctgacgggcttgtctgctcccggcatccgcttacagacaagctgtgaccgtctccgggagc
tgcatgtgtcagaggttttcaccgtcatcaccgaaacgcgcgaggcagctgcggtaaagctcatcagcgtggtcgtgaagcgattcacagatgtctgcctgttcatccgcgtcc
agctcgttgagtttctccagaagcgttaatgtctggcttctgataaagcgggccatgttaagggcggttttttcctgtttggtcactgatgcctccgtgtaagggggatttctgttca
tgggggtaatgataccgatgaaacgagagaggatgctcacgatacgggttactgatgatgaacatgcccggttactggaacgttgtgagggtaaacaactggcggtatggat
gcggcgggaccagagaaaaatcactcagggtcaatgccagcgcttcgttaatacagatgtaggtgttccacagggtagccagcagcatcctgcgatgcagatccggaacata
atggtgcagggcgctgacttccgcgtttccagactttacgaaacacggaaaccgaagaccattcatgttgttgctcaggtcgcagacgttttgcagcagcagtcgcttcacgttc
gctcgcgtatcggtgattcattctgctaaccagtaaggcaaccccgccagcctagccgggtcctcaacgacaggagcacgatcatgcgcacccgtggggccgccatgccggcg
ataatggcctgcttctcgccgaaacgtttggtggcgggaccagtgacgaaggcttgagcgagggcgtgcaagattccgaataccgcaagcgacaggccgatcatcgtcgcgc
tccagcgaaagcggtcctcgccgaaaatgacccagagcgctgccggcacctgtcctacgagttgcatgataaagaagacagtcataagtgcggcgacgatagtcatgccccg
cgcccaccggaaggagctgactgggttgaaggctctcaagggcatcggtcgagatcccggtgcctaatgagtgagctaacttacattaattgcgttgcgctcactgcccgcttt
ccagtcgggaaacctgtcgtgccagctgcattaatgaatcggccaacgcgcggggagaggcggtttgcgtattgggcgccagggtggtttttcttttcaccagtgagacgggca
acagctgattgcccttcaccgcctggccctgagagagttgcagcaagcggtccacgctggtttgccccagcaggcgaaaatcctgtttgatggtggttaacggcgggatataac
atgagctgtcttcggtatcgtcgtatcccactaccgagatatccgcaccaacgcgcagcccggactcggtaatggcgcgcattgcgcccagcgccatctgatcgttggcaacca
gcatcgcagtgggaacgatgccctcattcagcatttgcatggtttgttgaaaaccggacatggcactccagtcgccttcccgttccgctatcggctgaatttgattgcgagtgag
atatttatgccagccagccagacgcagacgcgccgagacagaacttaatgggcccgctaacagcgcgatttgctggtgacccaatgcgaccagatgctccacgcccagtcgc

gtaccgtcttcatgggagaaaataatactgttgatgggtgtctggtcagagacatcaagaaataacgccggaacattagtgcaggcagcttccacagcaatggcatcctggtc
atccagcggatagttaatgatcagcccactgacgcgttgcgcgagaagattgtgcaccgccgctttacaggcttcgacgccgcttcgttctaccatcgacaccaccacgctggc
acccagttgatcggcgcgagatttaatcgccgcgacaatttgcgacggcgcgtgcagggccagactggaggtggcaacgccaatcagcaacgactgtttgcccgccagttgtt
gtgccacgcggttgggaatgtaattcagctccgccatcgccgcttccactttttcccgcgttttcgcagaaacgtggctggcctggttcaccacgcgggaaacggtctgataaga
gacaccggcatactctgcgacatcgtataacgttactggtttcacattcaccaccctgaattgactctcttccgggcgctatcatgccataccgcgaaaggttttgcgccattcga
tggtgtccgggatctcgacgctctcccttatgcgactcctgcattaggaagcagcccagtagtaggttgaggccgttgagcaccgccgccgcaaggaatggtgcatgcaagga
gatggcgcccaacagtcccccggccacggggcctgccaccatacccacgccgaaacaagcgctcatgagcccgaagtggcgagcccgatcttccccatcggtgatgtcggcg
atataggcgccagcaaccgcacctgtggcgccggtgatgccggccacgatgcgtccggcgtagaggatcg

Sequence of the plasmid pET22/42-LptCΔTM-thrombin-His$_7$

agatctcgatcccgcgaaattaatacgactcactataggggaattgtgagcggataacaattcccctctagaaataattttgtttaactttaagaaggagatatacat**ATGGG**
TAAGGCAAAAAAACTTGATGTCGACTCGGCTCGAAATATGACTTATTCGGCCGAGGGAGTAAATCTTACACAAACGGATGATCAA
GGGCATTTGTATGTACGTGCAACAGTAGATCGTCTAGAGCAAAATATGCAAAATCAGACATCTAAGTTGGAAAATCTGAATGCAT
CCACATATAAAGCTGGCAATGTCGATTCAACATTTTTTTCTAAATATGCCTATGGTTACAATGACAATGAAAAGGTTGTCCTTTCAG
ATCAAGTTGTTGCAACTAAACTCTCTGATCATGGAAAAATGCAATTTCAAACTGATGAGTTAACCACCTATCCTCCGAAAAAAATG
ATTGAGACGACTCATCAAGTCAATGTGGATTCTCCACAGTCATCTTTTATCAGTCAGGGATTAAAAGCAGATTTAAACACTGGTCA
ATACGAGTTTTTAATATTCGAGGAAAGTATGCACCGAATTCTCTGGTACCGCGCGGCAGCCACCACCACCACCACCACCACTAAttg
attaatacctaggctgctaaacaaagcccgaaaggaagctgagttggctgctgccaccgctgagcaataactagcataaccccttggggcctctaaacgggtcttgaggggtt
ttttgctgaaaggaggaactatatccggattggcgaatgggacgcgccctgtagcggcgcattaagcgcggcgggtgtggtggttacgcgcagcgtgaccgctacacttgcca
gcgccctagcgcccgctcctttcgctttcttcccttcctttctcgccacgttcgccggctttccccgtcaagctctaaatcgggggctccctttagggttccgatttagtgctttacgg
cacctcgaccccaaaaaacttgattagggtgatggttcacgtagtgggccatcgccctgatagacggtttttcgccctttgacgttggagtccacgttctttaatagtggactctt
gttccaaactggaacaacactcaaccctatctcggtctattcttttgatttataagggattttgccgatttcggcctattggttaaaaaatgagctgatttaacaaaaatttaacgc
gaattttaacaaaatattaacgcttacaatttaggtggcacttttcggggaaatgtgcgcggaacccctatttgtttatttttctaaatacattcaaatatgtatccgctcatgaga
caataaccctgataaatgcttcaataatattgaaaaaggaagagtatgagtattcaacatttccgtgtcgcccttattcccttttttgcggcattttgccttcctgtttttgctcaccc
agaaacgctggtgaaagtaaaagatgctgaagatcagttgggtgcacgagtgggttacatcgaactggatctcaacagcggtaagatccttgagagttttcgccccgaagaa
cgttttccaatgatgagcacttttaaagttctgctatgtggcgcggtattatcccgtattgacgccgggcaagagcaactcggtcgccgcatacactattctcagaatgacttggt
tgagtactcaccagtcacagaaaagcatcttacggatggcatgacagtaagagaattatgcagtgctgccataaccatgagtgataacactgcggccaacttacttctgacaa
cgatcggaggaccgaaggagctaaccgcttttttgcacaacatgggggatcatgtaactcgccttgatcgttgggaaccggagctgaatgaagccataccaaacgacgagcg
tgacaccacgatgcctgcagcaatggcaacaacgttgcgcaaactattaactggcgaactacttactctagcttcccggcaacaattaatagactggatggaggcggataaag
ttgcaggaccacttctgcgctcggcccttccggctggctggtttattgctgataaatctggagccggtgagcgtgggtctcgcggtatcattgcagcactggggccagatggtaa
gccctcccgtatcgtagttatctacacgacggggagtcaggcaactatggatgaacgaaatagacagatcgctgagataggtgcctcactgattaagcattggtaactgtcag
accaagtttactcatatatactttagattgatttaaaacttcatttttaatttaaaaggatctaggtgaagatcctttttgataatctcatgaccaaaatcccttaacgtgagttttc
gttccactgagcgtcagaccccgtagaaaagatcaaaggatcttcttgagatcctttttttctgcgcgtaatctgctgcttgcaaacaaaaaaaccaccgctaccagcggtggttt
gtttgccggatcaagagctaccaactctttttccgaaggtaactggcttcagcagagcgcagataccaaatactgtccttctagtgtagccgtagttaggccaccacttcaagaa
ctctgtagcaccgcctacatacctcgctctgctaatcctgttaccagtggctgctgccagtggcgataagtcgtgtcttaccgggttggactcaagacgatagttaccggataag
gcgcagcggtcgggctgaacggggggttcgtgcacacagcccagcttggagcgaacgacctacaccgaactgagatacctacagcgtgagctatgagaaagcgccacgctt
cccgaagggagaaaggcggacaggtatccggtaagcggcagggtcggaacaggagagcgcacgagggagcttccagggggaaacgcctggtatctttatagtcctgtcgg
gtttcgccacctctgacttgagcgtcgatttttgtgatgctcgtcaggggggcggagcctatggaaaaacgccagcaacgcggcctttttacggttcctggccttttgctggccttt
tgctcacatgttctttcctgcgttatcccctgattctgtggataaccgtattaccgcctttgagtgagctgataccgctcgccgcagccgaacgaccgagcgcagcgagtcagtga
gcgaggaagcggaagagcgcctgatgcggtattttctccttacgcatctgtgcggtatttcacaccgcaatggtgcactctcagtacaatctgctctgatgccgcatagttaagc
cagtatacactccgctatcgctacgtgactgggtcatggctgcgccccgacacccgccaacacccgctgacgcgccctgacgggcttgtctgctcccggcatccgcttacagac
aagctgtgaccgtctccgggagctgcatgtgtcagaggttttcaccgtcatcaccgaaacgcgcgaggcagctgcggtaaagctcatcagcgtggtcgtgaagcgattcacag
atgtctgcctgttcatccgcgtccagctcgttgagtttctccagaagcgttaatgtctggcttctgataaagcgggccatgttaagggcggtttttttcctgtttggtcactgatgcct
ccgtgtaagggggatttctgttcatgggggtaatgataccgatgaaacgagagaggatgctcacgatacgggttactgatgatgaacatgcccggttactggaacgttgtgag
ggtaaacaactggcggtatggatgcggcgggaccagagaaaaatcactcagggtcaatgccagcgcttcgttaatacagatgtaggtgttccacagggtagccagcagcatc
ctgcgatgcagatccggaacataatggtgcagggcgctgacttccgcgtttccagactttacgaaacacggaaaccgaagaccattcatgttgttgctcaggtcgcagacgttt
tgcagcagcagtcgcttcacgttcgctcgcgtatcggtgattcattctgctaaccagtaaggcaaccccgccagcctagccgggtcctcaacgacaggagcacgatcatgcgca
cccgtggggccgccatgccggcgataatggcctgcttctcgccgaaacgtttggtggcgggaccagtgacgaaggcttgagcgagggcgtgcaagattccgaataccgcaag
cgacaggccgatcatcgtcgcgctccagcgaaagcggtcctcgccgaaaatgacccagagcgctgccggcacctgtcctacgagttgcatgataaagaagacagtcataagt

gcggcgacgatagtcatgccccgcgcccaccggaaggagctgactgggttgaaggctctcaagggcatcggtcgagatcccggtgcctaatgagtgagctaacttacattaat
tgcgttgcgctcactgcccgctttccagtcgggaaacctgtcgtgccagctgcattaatgaatcggccaacgcgcggggagaggcggtttgcgtattgggcgccagggtggtttt
tcttttcaccagtgagacgggcaacagctgattgcccttcaccgcctggccctgagagagttgcagcaagcggtccacgctggtttgccccagcaggcgaaaatcctgtttgat
ggtggttaacggcgggatataacatgagctgtcttcggtatcgtcgtatcccactaccgagatatccgcaccaacgcgcagcccggactcggtaatggcgcgcattgcgccca
gcgccatctgatcgttggcaaccagcatcgcagtgggaacgatgccctcattcagcatttgcatggtttgttgaaaaccggacatggcactccagtcgccttcccgttccgctat
cggctgaatttgattgcgagtgagatatttatgccagccagccagacgcagacgcgccgagacagaacttaatgggcccgctaacagcgcgatttgctggtgacccaatgcga
ccagatgctccacgcccagtcgcgtaccgtcttcatgggagaaaataatactgttgatgggtgtctggtcagagacatcaagaaataacgccggaacattagtgcaggcagct
tccacagcaatggcatcctggtcatccagcggatagttaatgatcagcccactgacgcgttgcgcgagaagattgtgcaccgccgctttacaggcttcgacgccgcttcgttcta
ccatcgacaccaccacgctggcacccagttgatcggcgcgagatttaatcgccgcgacaatttgcgacggcgcgtgcagggccagactggaggtggcaacgccaatcagcaa
cgactgtttgcccgccagttgttgtgccacgcggttgggaatgtaattcagctccgccatcgccgcttccactttttcccgcgttttcgcagaaacgtggctggcctggttcaccac
gcgggaaacggtctgataagagacaccggcatactctgcgacatcgtataacgttactggtttcacattcaccaccctgaattgactctcttccgggcgctatcatgccataccg
cgaaaggttttgcgccattcgatggtgtccgggatctcgacgctctcccttatgcgactcctgcattaggaagcagcccagtagtaggttgaggccgttgagcaccgccgccgc
aaggaatggtgcatgcaaggagatggcgcccaacagtcccccggccacggggcctgccaccatacccacgccgaaacaagcgctcatgagcccgaagtggcgagcccgat
cttccccatcggtgatgtcggcgatataggcgccagcaaccgcacctgtggcgccggtgatgccggccacgatgcgtccggcgtagaggatcg

Sequence of the plasmid pCDFduet-LptB-LptFG

gccataccgcgaaaggttttgcgccattcgatggtgtccgggatctcgacgctctcccttatgcgactcctgcattaggaaattaatacgactcactataggggaattgtgagcg
gataacaattcccctgtagaaataattttgtttaactttaataaggagatatacc**ATGGAGCAAATCGCGCAACAACAACCTCAGACTTTATGCATTAAG**
CATCTTGCAAAGAATTACAGCAAACGTTGGGTGGTAAAAGACGTATCGTTTGAGATGCAAAGTGGACAAATTGTTGGTTTGCTTG
GGCCGAATGGTGCTGGTAAAACAACCAGTTTCTATATGGTTGTCGGGTTGGTGCGCATGGATAAAGGTGAAATTCATCTTGATAA
TCTTGATTTGTCTGATCTAGCTATGCACGAACGCGCGAGAAAGGGAATTGGTTATTTGCCTCAAGAAGCTTCTATTTTCAGAAAACT
TACGATTGCTGAAAATATTATGGCTATTCTTGAGACTCGAAAAGACCTGAATAAGCAACAGCGTCAGCAACGTCTCCAAGAGTTAT
TGAATGATTTTAAAATCACGCATATTAAAGATTCTTTAGGGATGAGTGTATCTGGTGGTGAGCGACGACGTGCTGAAATTGCGCGT
GCATTGGCCGCAGACCCAAAGTTTATGCTGCTTGATGAACCTTTTGCGGGGGTGGATCCGATTTCGGTCGGTGATATTAAAGACAT
TATTCGTAATTTAAAAGATCGCGGTATTGGCGTACTCATTACCGATCATAATGTGCGTGAAACTTTGGCTATCTGTGAGCATGCTTA
CATTGTAAGTGAAGGCGCTGTAATTGCAGAGGGTTCTCCGCAGGATATTTTAGAAAATGAACAGGTACGTAAGGTATATCTAGGA
GATGATTTTACAGTCTAAgaattcgagctcggcgcgcctgcaggtcgacaagcttgcggccgcataatgcttaagtcgaacagaaagtaatcgtattgtacacggccg
cataatcgaaattaatacgactcactataggggaattgtgagcggataacaattccccatcttagtatattagttaagtataagaaggagatatacat**ATGATTATTCGA**
CGTTATCTTGTCAAACAAGTGGTATCGACGTCCTTGGTAGTGATTGCCTTATTGACCTTAATCATGATGGGTGGTCGTTTAATCAAG
TATTTTGGCGTGGCTGCTCAGGGTCGTTTGGATGCTGGCGTACTGTTTAGCATCATTGGCTATCGTATGCCTGAATTTTTAACCCTG
ATCTTACCGTTAGGATTCTTTATTGGTTTGATGCTGGTGTTTGGTCGACTTTACGTTGATCATGAAATGGCTGTACTCAATGGCAGT
GGCATCAGTCGAATTCGACTTGGACAGCTATTGATTCCACTGGCTTTGGTCTTCTTGGTGATACAAGGCATTTTGATGCTATGGATG
ACGCCTTGGGGGCTACGCCAGTTTGATCAACTCTCCAGTAGTCAGGCTGTTCGTACAGGCTTTGATTTAGTCAGACCCAAAGAATTT
ATATCATCGGGGCCTTATACCATCTATGCAGGGGATTTATCCGAAGACCGTAAAAACCTGAAAGATATTTTCTTCTACCAGCGAGC
ACAAAAAGAAGGCAAACCCGATGTCATGATTCTGGCCAAAGAAGCCACGCGTGTGGTCATGGAAAATGAAACGGCCAATGTAGT
GGACTTGATTCAGGGACGTCGCTACGAAATTTATCCAGGAAAGGCAAAATATTCACAGGCCGAATTCCAGCGCTATCGTTTACGTC
TAGAAAATGATAAGTCGGCAACCTTTGAAACTGACAAAGTTGAGGCATTGCCAAGTTCAAAACTCTGGAACAAATGGAACGATCC
AGTCATTGCCAGTGAAATGGGCTGGCGTGTATTTGGCCCTTTTACCATTGTCATTGCCTTGATGATGGCTGTTGCACTGTGTGAGGT
AAGTCCGCGTCAGGGACGTTATTATCGACTTATTCCTGCGATTTTTATCTTTGCGAGTTTAATTGTATTGTTAATCGCAATTCGTACA
CGTATTAGTCGTGATGAACTAGGTGTATGGGCTTATCCAGCAGCCTTGGCTGTGTATGGTATTGCTGCGGCGTTATTCTCACGCAA
GCAAAAGCTGGCGCCTAAAATTAAGAAACAGATCAAACGAGTGAGAGCATA***ATGTTAGCACGTCGAATCGTCGCAAAGCATGTG***
ACCAAAACCACGGCGCTAGCAATGCTAGGAACCACGATTGTTTTGGTGATTTTGCAGGTTTTATTTACCTATTTAGGTGAGCTGTCC
AATCTTAAAGCAGACTATAGCGCCTGGCAGGCATTTTTATATGTTTTATGGGGAGCACCGCGCTATCTCTATGAAATTCTGCCTATCT
CGGCGTTGATTGGTGCAATTTTAGGATTGGGTACACTGGCATCCAACAGTGAATTGATTGTGATGCGCTCGGTGGGAATCAGTTTA
TGGCGGATTGTCGGTTGGGTCATTCGTTCGGCATTAGTACTGGTTTTACTCTCGTTTGCATTGAGTGAGTGGGTCGTGCCGTATACC
AATGAGCGAGCAAATAGCGTGAAGAGCCATCAAAGTGTTGCGGCACTGGGCGAGGTACGAGGTTACTGGTCACGTGAAGGACAG
CGCTTTATTTATGTGGATTATGCCAACTCACAAGGTCAGCTTAAACGGATTCAGGTGGTCGATTTTGATGACAACTATCGTTTAAAG
TCGGTAACCAATGCCGAGCAAGGACAGTTTGTCAAAGATGGTCAATGGTTGTTAAATCATTCGCAGCAGATGGCGATTCAGGGAC
AAGGCGATGCTGTATTGGCAAATGCAGCTAAACAGCCATTTTCATTGGCATTGCAGCCTAAGTACGTGCATATGGTGACGATTGAT

CCAGAAGATTTATCCTTTAGCCAACTGGTCAGTTTTATGAACTATATGCGTGAATACAGCCAAGTGCCGAAAACCTATCAGTTGGCA
TTCTGGAAGAAAGTGGCTTCACCTTTTGCATTGATTACGTTGGTATTGGTTGCCTGTTCTTTTATTTTTGGGCCACTACGCCAGCAAT
CGATGGGTTTTCGCTTGGTGATCGCGCTGTTTATTGGACTAGGTTTTTACTATTTACAAGATTTTCTTGGATATGCAAGTTTGGTTTA
TAACCCATCACCCGCATGGTTTGTACTTGGGCCAATTGTACTCATGTTTGTTGCGGGGAGTTACTTGTTATATCGGGCAAGATAAggt
accctcgagtctggtaaagaaaccgctgctgcgaaatttgaacgccagcacatggactcgtctactagcgcagcttaattaacctaggctgctgccaccgctgagcaataacta
gcataaccccttggggcctctaaacgggtcttgaggggttttttgctgaaacctcaggcatttgagaagcacacggtcacactgcttccggtagtcaataaaccggtaaaccag
caatagacataagcggctatttaacgaccctgccctgaaccgacgaccgggtcatcgtggccggatcttgcggcccctcggcttgaacgaattgttagacattatttgccgact
accttggtgatctcgcctttcacgtagtggacaaattcttccaactgatctgcgcgcgaggccaagcgatcttcttcttgtccaagataagcctgtctagcttcaagtatgacggg
ctgatactgggccggcaggcgctccattgcccagtcggcagcgacatccttcggcgcgattttgccggttactgcgctgtaccaaatgcgggacaacgtaagcactacatttcg
ctcatcgccagcccagtcgggcggcgagttccatagcgttaaggtttcatttagcgcctcaaatagatcctgttcaggaaccggatcaaagagttcctccgccgctggacctacc
aaggcaacgctatgttctcttgcttttgtcagcaagatagccagatcaatgtcgatcgtggctggctcgaagatacctgcaagaatgtcattgcgctgccattctccaaattgca
gttcgcgcttagctggataacgccacggaatgatgtcgtcgtgcacaacaatggtgacttctacagcgcggagaatctcgctctctccaggggaagccgaagtttccaaaagg
tcgttgatcaaagctcgccgcgttgtttcatcaagccttacggtcaccgtaaccagcaaatcaatatcactgtgtggcttcaggccgccatccactgcggagccgtacaaatgta
cggccagcaacgtcggttcgagatggcgctcgatgacgccaactacctctgatagttgagtcgatacttcggcgatcaccgcttccctcatactcttcctttttcaatattattgaa
gcatttatcagggttattgtctcatgagcggatacatatttgaatgtatttagaaaaataaacaaatagctagctcactcggtcgctacgctccgggcgtgagactgcggcgggc
gctgcggacacatacaaagttacccacagattccgtggataagcaggggactaacatgtgaggcaaaacagcagggccgcgccggtggcgtttttccataggctccgccctc
ctgccagagttcacataaacagacgcttttccggtgcatctgtgggagccgtgaggctcaaccatgaatctgacagtacgggcgaaacccgacaggacttaaagatccccacc
gtttccggcgggtcgctccctcttgcgctctcctgttccgaccctgccgtttaccggatacctgttccgcctttctcccttacgggaagtgtggcgctttctcatagctcacacactg
gtatctcggctcggtgtaggtcgttcgctccaagctgggctgtaagcaagaactccccgttcagcccgactgctgcgccttatccggtaactgttcacttgagtccaacccggaa
aagcacggtaaaacgccactggcagcagccattggtaactgggagttcgcagaggatttgtttagctaaacacgcggttgctcttgaagtgtgcgccaaagtccggctacact
ggaaggacagatttggttgctgtgctctgcgaaagccagttaccacggttaagcagttccccaactgacttaaccttcgatcaaaccacctccccaggtggttttttcgtttacag
ggcaaaagattacgcgcagaaaaaaaggatctcaagaagatcctttgatcttttctactgaaccgctctagatttcagtgcaatttatctcttcaaatgtagcacctgaagtcag
ccccatacgatataagttgtaattctcatgttagtcatgccccgcgcccaccggaaggagctgactgggttgaaggctctcaagggcatcggtcgagatcccggtgcctaatga
gtgagctaacttacattaattgcgttgcgctcactgcccgctttccagtcgggaaacctgtcgtgccagctgcattaatgaatcggccaacgcgcggggagaggcggtttgcgta
ttgggcgccagggtggtttttcttttcaccagtgagacgggcaacagctgattgcccttcaccgcctggccctgagagagttgcagcaagcggtccacgctggtttgccccagca
ggcgaaaatcctgtttgatggtggttaacggcgggatataacatgagctgtcttcggtatcgtcgtatcccactaccgagatgtccgcaccaacgcgcagcccggactcggtaa
tggcgcgcattgcgcccagcgccatctgatcgttggcaaccagcatcgcagtgggaacgatgccctcattcagcatttgcatggtttgttgaaaaccggacatggcactccagt
cgccttcccgttccgctatcggctgaatttgattgcgagtgagatatttatgccagccagccagacgcagacgcgccgagacagaacttaatgggcccgctaacagcgcgattt
gctggtgacccaatgcgaccagatgctccacgcccagtcgcgtaccgtcttcatgggagaaaataatactgttgatgggtgtctggtcagagacatcaagaaataacgccgga
acattagtgcaggcagcttccacagcaatggcatcctggtcatccagcggatagttaatgatcagcccactgacgcgttgcgcgagaagattgtgcaccgccgctttacaggct
tcgacgccgcttcgttctaccatcgacaccaccacgctggcacccagttgatcggcgcgagatttaatcgccgcgacaatttgcgacggcgcgtgcagggccagactggaggt
ggcaacgccaatcggcaacgactgtttgcccgccagttgttgtgccacgcggttgggaatgtaattcagctccgccatcgccgcttccactttttcccgcgttttcgcagaaacgt
ggctggcctggttcaccacgcgggaaacggtctgataagagacaccggcatactctgcgacatcgtataacgttactggtttcacattcaccaccctgaattgactctcttccgg
gcgctatcat

Sequence of the plasmid pCDFduet-HisLptB-LptFG

gccataccgcgaaaggttttgcgccattcgatggtgtccgggatctcgacgctctcccttatgcgactcctgcattaggaaattaatacgactcactataggggaattgtgagcg
gataacaattcccctgtagaaataattttgtttaactttaataaggagatatacc**ATGCACCACCACCACCACCACATGGAGCAAATCGCGCAACAA**
CAACCTCAGACTTTATGCATTAAGCATCTTGCAAAGAATTACAGCAAACGTTGGGTGGTAAAAGACGTATCGTTTGAGATGCAAA
GTGGACAAATTGTTGGTTTGCTTGGGCCGAATGGTGCTGGTAAAACAACCAGTTTCTATATGGTTGTCGGGTTGGTGCGCATGGAT
AAAGGTGAAATTCATCTTGATAATCTTGATTTGTCTGATCTAGCTATGCACGAACGCGCGAGAAAGGGAATTGGTTATTTGCCTCA
AGAAGCTTCTATTTTCAGAAAACTTACGATTGCTGAAAATATTATGGCTATTCTTGAGACTCGAAAAGACCTGAATAAGCAACAGC
GTCAGCAACGTCTCCAAGAGTTATTGAATGATTTTAAAATCACGCATATTAAAGATTCTTTAGGGATGAGTGTATCTGGTGGTGAG
CGACGACGTGCTGAAATTGCGCGTGCATTGGCCGCAGACCCAAAGTTTATGCTGCTTGATGAACCTTTTGCGGGGGTGGATCCGAT
TTCGGTCGGTGATATTAAAGACATTATTCGTAATTTAAAAGATCGCGGTATTGGCGTACTCATTACCGATCATAATGTGCGTGAAA
CTTTGGCTATCTGTGAGCATGCTTACATTGTAAGTGAAGGCGCTGTAATTGCAGAGGGTTCTCCGCAGGATATTTTAGAAAATGAA
CAGGTACGTAAGGTATATCTAGGAGATGATTTTACAGTCTAAgaattcgagctcggcgcgcctgcaggtcgacaagcttgcggccgcataatgcttaagt
cgaacagaaagtaatcgtattgtacacggccgcataatcgaaattaatacgactcactataggggaattgtgagcggataacaattccccatcttagtatattagttaagtata
agaaggagatatacat**ATGATTATTCGACGTTATCTTGTCAAACAAGTGGTATCGACGTCCTTGGTAGTGATTGCCTTATTGACCTTAATC**

ATGATGGGTGGTCGTTTAATCAAGTATTTTGGCGTGGCTGCTCAGGGTCGTTTGGATGCTGGCGTACTGTTTAGCATCATTGGCTAT
CGTATGCCTGAATTTTTAACCCTGATCTTACCGTTAGGATTCTTTATTGGTTTGATGCTGGTGTTTGGTCGACTTTACGTTGATCATG
AAATGGCTGTACTCAATGGCAGTGGCATCAGTCGAATTCGACTTGGACAGCTATTGATTCCACTGGCTTTGGTCTTCTTGGTGATAC
AAGGCATTTTGATGCTATGGATGACGCCTTGGGGGCTACGCCAGTTTGATCAACTCTCCAGTAGTCAGGCTGTTCGTACAGGCTTT
GATTTAGTCAGACCCAAAGAATTTATATCATCGGGGCCTTATACCATCTATGCAGGGGATTTATCCGAAGACCGTAAAAACCTGAA
AGATATTTTCTTCTACCAGCGAGCACAAAAAGAAGGCAAACCCGATGTCATGATTCTGGCCAAAGAAGCCACGCGTGTGGTCATG
GAAAATGAAACGGCCAATGTAGTGGACTTGATTCAGGGACGTCGCTACGAAATTTATCCAGGAAAGGCAAAATATTCACAGGCCG
AATTCCAGCGCTATCGTTTACGTCTAGAAAATGATAAGTCGGCAACCTTTGAAACTGACAAAGTTGAGGCATTGCCAAGTTCAAAA
CTCTGGAACAAATGGAACGATCCAGTCATTGCCAGTGAAATGGGCTGGCGTGTATTTGGCCCTTTTACCATTGTCATTGCCTTGATG
ATGGCTGTTGCACTGTGTGAGGTAAGTCCGCGTCAGGGACGTTATTATCGACTTATTCCTGCGATTTTTATCTTTGCGAGTTTAATT
GTATTGTTAATCGCAATTCGTACACGTATTAGTCGTGATGAACTAGGTGTATGGGCTTATCCAGCAGCCTTGGCTGTGTATGGTATT
GCTGCGGCGTTATTCTCACGCAAGCAAAAGCTGGCGCCTAAAATTAAGAAACAGATCAAACGAGTGAGAGCATA***ATGTTAGCACG***
TCGAATCGTCGCAAAGCATGTGACCAAAACCACGGCGCTAGCAATGCTAGGAACCACGATTGTTTTGGTGATTTTGCAGGTTTTATT
TACCTATTTAGGTGAGCTGTCCAATCTTAAAGCAGACTATAGCGCCTGGCAGGCATTTTTATATGTTTTATGGGGAGCACCGCGCTA
TCTCTATGAAATTCTGCCTATCTCGGCGTTGATTGGTGCAATTTTAGGATTGGGTACACTGGCATCCAACAGTGAATTGATTGTGAT
GCGCTCGGTGGGAATCAGTTTATGGCGGATTGTCGGTTGGGTCATTCGTTCGGCATTAGTACTGGTTTTACTCTCGTTTGCATTGAG
TGAGTGGGTCGTGCCGTATACCAATGAGCGAGCAAATAGCGTGAAGAGCCATCAAAGTGTTGCGGCACTGGGCGAGGTACGAGG
TTACTGGTCACGTGAAGGACAGCGCTTTATTTATGTGGATTATGCCAACTCACAAGGTCAGCTTAAACGGATTCAGGTGGTCGATTT
TGATGACAACTATCGTTTAAAGTCGGTAACCAATGCCGAGCAAGGACAGTTTGTCAAAGATGGTCAATGGTTGTTAAATCATTCGC
AGCAGATGGCGATTCAGGGACAAGGCGATGCTGTATTGGCAAATGCAGCTAAACAGCCATTTTCATTGGCATTGCAGCCTAAGTAC
GTGCATATGGTGACGATTGATCCAGAAGATTTATCCTTTAGCCAACTGGTCAGTTTTATGAACTATATGCGTGAATACAGCCAAGTG
CCGAAAACCTATCAGTTGGCATTCTGGAAGAAAGTGGCTTCACCTTTTGCATTGATTACGTTGGTATTGGTTGCCTGTTCTTTTATTT
TTGGGCCACTACGCCAGCAATCGATGGGTTTTCGCTTGGTGATCGCGCTGTTTATTGGACTAGGTTTTTACTATTTACAAGATTTTCT
TGGATATGCAAGTTTGGTTTATAACCCATCACCCGCATGGTTTGTACTTGGGCCAATTGTACTCATGTTTGTTGCGGGGAGTTACTT
GTTATATCGGGCAAGATAAggtaccctcgagtctggtaaagaaaccgctgctgcgaaatttgaacgccagcacatggactcgtctactagcgcagcttaattaacct
aggctgctgccaccgctgagcaataactagcataaccccttggggcctctaaacgggtcttgaggggttttttgctgaaacctcaggcatttgagaagcacacggtcacactgc
ttccggtagtcaataaaccggtaaaccagcaatagacataagcggctatttaacgaccctgccctgaaccgacgaccgggtcatcgtggccggatcttgcggcccctcggcttg
aacgaattgttagacattatttgccgactaccttggtgatctcgcctttcacgtagtggacaaattcttccaactgatctgcgcgcgaggccaagcgatcttcttcttgtccaagat
aagcctgtctagcttcaagtatgacgggctgatactgggccggcaggcgctccattgcccagtcggcagcgacatccttcggcgcgattttgccggttactgcgctgtaccaaat
gcgggacaacgtaagcactacatttcgctcatcgccagcccagtcgggcggcgagttccatagcgttaaggtttcatttagcgcctcaaatagatcctgttcaggaaccggatc
aaagagttcctccgccgctggacctaccaaggcaacgctatgttctcttgcttttgtcagcaagatagccagatcaatgtcgatcgtggctggctcgaagatacctgcaagaat
gtcattgcgctgccattctccaaattgcagttcgcgcttagctggataacgccacggaatgatgtcgtcgtgcacaacaatggtgacttctacagcgcggagaatctcgctctct
ccaggggaagccgaagtttccaaaaggtcgttgatcaaagctcgccgcgttgtttcatcaagccttacggtcaccgtaaccagcaaatcaatatcactgtgtggcttcaggccg
ccatccactgcggagccgtacaaatgtacggccagcaacgtcggttcgagatggcgctcgatgacgccaactacctctgatagttgagtcgatacttcggcgatcaccgcttcc
ctcatactcttcctttttcaatattattgaagcatttatcagggttattgtctcatgagcggatacatatttgaatgtatttagaaaaataaacaaatagctagctcactcggtcgct
acgctccgggcgtgagactgcggcgggcgctgcggacacatacaaagttacccacagattccgtggataagcaggggactaacatgtgaggcaaaacagcagggccgcgc
cggtggcgtttttccataggctccgcccctcctgccagagttcacataaacagacgcttttccggtgcatctgtgggagccgtgaggctcaaccatgaatctgacagtacgggcga
aacccgacaggacttaaagatccccaccgtttccggcgggtcgctccctcttgcgctctcctgttccgaccctgccgtttaccggatacctgttccgcctttctcccttacgggaag
tgtggcgctttctcatagctcacacactggtatctcggctcggtgtaggtcgttcgctccaagctgggctgtaagcaagaactccccgttcagcccgactgctgcgccttatccgg
taactgttcacttgagtccaacccggaaaagcacggtaaaacgccactggcagcagccattggtaactgggagttcgcagaggatttgtttagctaaacacgcggttgctcttg
aagtgtgcgccaaagtccggctacactggaaggacagatttggttgctgtgctctgcgaaagccagttaccacggttaagcagttccccaactgacttaaccttcgatcaaacc
acctccccaggtggtttttttcgtttacagggcaaaagattacgcgcagaaaaaaaggatctcaagaagatcctttgatcttttctactgaaccgctctagatttcagtgcaattta
tctcttcaaatgtagcacctgaagtcagccccatacgatataagttgtaattctcatgttagtcatgccccgcgcccaccggaaggagctgactgggttgaaggctctcaagggc
atcggtcgagatcccggtgcctaatgagtgagctaacttacattaattgcgttgcgctcactgcccgctttccagtcgggaaacctgtcgtgccagctgcattaatgaatcggcc
aacgcgcggggagaggcggtttgcgtattgggcgccagggtggtttttcttttcaccagtgagacgggcaacagctgattgcccttcaccgcctggccctgagagagttgcagc
aagcggtccacgctggtttgccccagcaggcgaaaatcctgtttgatggtggttaacggcgggatataacatgagctgtcttcggtatcgtcgtatcccactaccgagatgtccg
caccaacgcgcagcccggactcggtaatggcgcgcattgcgcccagcgccatctgatcgttggcaaccagcatcgcagtgggaacgatgccctcattcagcatttgcatggttt
gttgaaaaccggacatggcactccagtcgccttcccgttccgctatcggctgaatttgattgcgagtgagatatttatgccagccagccagacgcagacgcgccgagacagaa
cttaatgggcccgctaacagcgcgatttgctggtgacccaatgcgaccagatgctccacgcccagtcgcgtaccgtcttcatgggagaaaataatactgttgatgggtgtctgg

tcagagacatcaagaaataacgccggaacattagtgcaggcagcttccacagcaatggcatcctggtcatccagcggatagttaatgatcagcccactgacgcgttgcgcga
gaagattgtgcaccgccgctttacaggcttcgacgccgcttcgttctaccatcgacaccaccacgctggcacccagttgatcggcgcgagatttaatcgccgcgacaatttgcg
acggcgcgtgcagggccagactggaggtggcaacgccaatcggcaacgactgtttgcccgccagttgttgtgccacgcggttgggaatgtaattcagctccgccatcgccgctt
ccactttttcccgcgttttcgcagaaacgtggctggcctggttcaccacgcgggaaacggtctgataagagacaccggcatactctgcgacatcgtataacgttactggtttcac
attcaccaccctgaattgactctcttccgggcgctatcat

Sequence of the plasmid pTRAB-FLAGLptB-LptFG

gtttgacagcttatcatcgactgcacggtgcaccaatgcttctggcgtcaggcagccatcggaagctgtggtatggctgtgcaggtcgtaaatcactgcataattcgtgtcgctc
aaggcgcactcccgttctggataatgttttttgcgccgacatcataacggttctggcaaatattctgaaatgagctgttgacaattaatcatccggctcgtataatgtgtggaatt
gtgagcggataacaatttcacacaggaaacagcatatggttaggaagaacgcataataacg**ATGGACTATAAGGATGATGATGATAAGGAGCAAATCG**
CGCAACAACAACCTCAGACTTTATGCATTAAGCATCTTGCAAAGAATTACAGCAAACGTTGGGTGGTAAAAGACGTATCGTTTGAG
ATGCAAAGTGGACAAATTGTTGGTTTGCTTGGGCCGAATGGTGCTGGTAAAACAACCAGTTTCTATATGGTTGTCGGGTTGGTGCG
CATGGATAAAGGTGAAATTCATCTTGATAATCTTGATTTGTCTGATCTAGCTATGCACGAACGCGCGAGAAAGGGAATTGGTTATT
TGCCTCAAGAAGCTTCTATTTTCAGAAAACTTACGATTGCTGAAAATATTATGGCTATTCTTGAGACTCGAAAAGACCTGAATAAGC
AACAGCGTCAGCAACGTCTCCAAGAGTTATTGAATGATTTTAAAATCACGCATATTAAAGATTCTTTAGGGATGAGTGTATCTGGT
GGTGAGCGACGACGTGCTGAAATTGCGCGTGCATTGGCCGCAGACCCAAAGTTTATGCTGCTTGATGAACCTTTTGCGGGGGTGG
ATCCGATTTCGGTCGGTGATATTAAAGACATTATTCGTAATTTAAAAGATCGCGGTATTGGCGTACTCATTACCGATCATAATGTGC
GTGAAACTTTGGCTATCTGTGAGCATGCTTACATTGTAAGTGAAGGCGCTGTAATTGCAGAGGGTTCTCCGCAGGATATTTTAGAA
AATGAACAGGTACGTAAGGTATATCTAGGAGATGATTTTACAGTCTAGgtgggatctgagagggacccg**ATGATTATTCGACGTTATCTTG**
TCAAACAAGTGGTATCGACGTCCTTGGTAGTGATTGCCTTATTGACCTTAATCATGATGGGTGGTCGTTTAATCAAGTATTTTGGCG
TGGCTGCTCAGGGTCGTTTGGATGCTGGCGTACTGTTTAGCATCATTGGCTATCGTATGCCTGAATTTTTAACCCTGATCTTACCGTT
AGGATTCTTTATTGGTTTGATGCTGGTGTTTGGTCGACTTTACGTTGATCATGAAATGGCTGTACTCAATGGCAGTGGCATCAGTCG
AATTCGACTTGGACAGCTATTGATTCCACTGGCTTTGGTCTTCTTGGTGATACAAGGCATTTTGATGCTATGGATGACGCCTTGGGG
GCTACGCCAGTTTGATCAACTCTCCAGTAGTCAGGCTGTTCGTACAGGCTTTGATTTAGTCAGACCCAAAGAATTTATATCATCGGG
GCCTTATACCATCTATGCAGGGGATTTATCCGAAGACCGTAAAAACCTGAAAGATATTTTCTTCTACCAGCGAGCACAAAAAGAAG
GCAAACCCGATGTCATGATTCTGGCCAAAGAAGCCACGCGTGTGGTCATGGAAAATGAAACGGCCAATGTAGTGGACTTGATTCA
GGGACGTCGCTACGAAATTTATCCAGGAAAGGCAAAATATTCACAGGCCGAATTCCAGCGCTATCGTTTACGTCTAGAAAATGAT
AAGTCGGCAACCTTTGAAACTGACAAAGTTGAGGCATTGCCAAGTTCAAAACTCTGGAACAAATGGAACGATCCAGTCATTGCCA
GTGAAATGGGCTGGCGTGTATTTGGCCCTTTTACCATTGTCATTGCCTTGATGATGGCTGTTGCACTGTGTGAGGTAAGTCCGCGTC
AGGGACGTTATTATCGACTTATTCCTGCGATTTTTATCTTTGCGAGTTTAATTGTATTGTTAATCGCAATTCGTACACGTATTAGTCG
TGATGAACTAGGTGTATGGGCTTATCCAGCAGCCTTGGCTGTGTATGGTATTGCTGCGGCGTTATTCTCACGCAAGCAAAAGCTGG
CGCCTAAAATTAAGAAACAGATCAAACGAGTGAGAGCATA*ATGTTAGCACGTCGAATCGTCGCAAAGCATGTGACCAAAACCACG*
GCGCTAGCAATGCTAGGAACCACGATTGTTTTGGTGATTTTGCAGGTTTTATTTACCTATTTAGGTGAGCTGTCCAATCTTAAAGCA
GACTATAGCGCCTGGCAGGCATTTTTATATGTTTTATGGGGAGCACCGCGCTATCTCTATGAAATTCTGCCTATCTCGGCGTTGATTG
GTGCAATTTTAGGATTGGGTACACTGGCATCCAACAGTGAATTGATTGTGATGCGCTCGGTGGGAATCAGTTTATGGCGGATTGTC
GGTTGGGTCATTCGTTCGGCATTAGTACTGGTTTTACTCTCGTTTGCATTGAGTGAGTGGGTCGTGCCGTATACCAATGAGCGAGCA
AATAGCGTGAAGAGCCATCAAAGTGTTGCGGCACTGGGCGAGGTACGAGGTTACTGGTCACGTGAAGGACAGCGCTTTATTTATG
TGGATTATGCCAACTCACAAGGTCAGCTTAAACGGATTCAGGTGGTCGATTTTGATGACAACTATCGTTTAAAGTCGGTAACCAAT
GCCGAGCAAGGACAGTTTGTCAAAGATGGTCAATGGTTGTTAAATCATTCGCAGCAGATGGCGATTCAGGGACAAGGCGATGCTG
TATTGGCAAATGCAGCTAAACAGCCATTTTCATTGGCATTGCAGCCTAAGTACGTGCATATGGTGACGATTGATCCAGAAGATTTAT
CCTTTAGCCAACTGGTCAGTTTTATGAACTATATGCGTGAATACAGCCAAGTGCCGAAAACCTATCAGTTGGCATTCTGGAAGAAA
GTGGCTTCACCTTTTGCATTGATTACGTTGGTATTGGTTGCCTGTTCTTTTATTTTTGGGCCACTACGCCAGCAATCGATGGGTTTTCG
CTTGGTGATCGCGCTGTTTATTGGACTAGGTTTTTACTATTTACAAGATTTTCTTGGATATGCAAGTTTGGTTTATAACCCATCACCC
GCATGGTTTGTACTTGGGCCAATTGTACTCATGTTTGTTGCGGGGAGTTACTTGTTATATCGGGCAAGATAAgatcctctagagtcgacctg
caggcatgcaagcttggctgttttggcggatgagagaagattttcagcctgatacagattaaatcagaacgcagaagcggtctgataaaacagaatttgcctggcggcagtag
cgcggtggtcccacctgaccccatgccgaactcagaagtgaaacgccgtagcgccgatggtagtgtggggtctccccatgcgagagtagggaactgccaggcatcaaataaa
acgaaaggctcagtcgaaagactgggcctttcgttttatctgttgtttgtcggtgaacgctctcctgagtaggacaaatccgccgggagcggatttgaacgttgcgaagcaacg
gcccggagggtggcgggcaggacgcccgccataaactgccaggcatcaaattaagcagaaggccatcctgacggatggcctttttgcgtttctacaaactctttttgtttattttt
ctaaatacattcaaatatgtatccgctcatgagacaataaccctgataaatgcttcaataatattgaaaaaggaagagtatgagggaagcggtgatcgccgaagtatcgactc

aactatcagaggtagttggcgtcatcgagcgccatctcgaaccgacgttgctggccgtacatttgtacggctccgcagtggatggcggcctgaagccacacagtgatattgatt
tgctggttacggtgaccgtaaggcttgatgaaacaacgcggcgagctttgatcaacgaccttttggaaacttcggcttcccctggagagagcgagattctccgcgctgtagaag
tcaccattgttgtgcacgacgacatcattccgtggcgttatccagctaagcgcgaactgcaatttggagaatggcagcgcaatgacattcttgcaggtatcttcgagccagccac
gatcgacattgatctggctatcttgctgacaaaagcaagagaacatagcgttgccttggtaggtccagcggcggaggaactctttgatccggttcctgaacaggatctatttga
ggcgctaaatgaaaccttaacgctatggaactcgccgcccgactgggctggcgatgagcgaaatgtagtgcttacgttgtcccgcatttggtacagcgcagtaaccggcaaaa
tcgcgccgaaggatgtcgctgccgactgggcaatggagcgcctgccggcccagtatcagcccgtcatacttgaagctagacaggcttatcttggacaagaagaagatcgcttg
gcctcgcgcgcagatcagttggaagaatttgtccactacgtgaaaggcgagatcaccaaggtagtcggcaaataactgtcagaccaagtttactcatatatactttagattgat
ttaaaacttcatttttaatttaaaaggatctaggtgaagatcctttttgataatctcatgaccaaaatcccttaacgtgagttttcgttccactgagcgtcagaccccgtagaaaa
gatcaaaggatcttcttgagatcctttttttctgcgcgtaatctgctgcttgcaaacaaaaaaaccaccgctaccagcggtggtttgtttgccggatcaagagctaccaactctttt
tccgaaggtaactggcttcagcagagcgcagataccaaatactgtccttctagtgtagccgtagttaggccaccacttcaagaactctgtagcaccgcctacatacctcgctctg
ctaatcctgttaccagtggctgctgccagtggcgataagtcgtgtcttaccgggttggactcaagacgatagttaccggataaggcgcagcggtcgggctgaacggggggttcg
tgcacacagcccagcttggagcgaacgacctacaccgaactgagatacctacagcgtgagctatgagaaagcgccacgcttcccgaagggagaaaggcggacaggtatccg
gtaagcggcagggtcggaacaggagagcgcacgagggagcttccagggggaaacgcctggtatctttatagtcctgtcgggtttcgccacctctgacttgagcgtcgatttttg
tgatgctcgtcaggggggcggagcctatggaaaaacgccagcaacgcggatcgtagaaatatctatgattatcttgaagaacgcaaccctatagcagctattgaaattgatga
tttaattgaagaaaagacagatttagttgttgataatcgactgatggggcgcacaggcagacagaaagatactagggagttagtgatacatccgcattatgtggttgtatatga
catcactgatataatacggatactcagagtgctacacacatcgcaggagtggtcatgacttactcatgtactttggattatttagtgttataaaatcctgatttataaattttttttg
ttaaaaaagataaaagccccttgcaattgcttggggctttaccgtaatttatggggtacagatcttcgatactgacatatcggcaatcgaaagcattaaggtttgacgaccgcta
atgatttcaccacaggggcttaatgtacctgtcttaaattctaaggttttaactcgctttgtcaagcatagaccccaaaaatttagccaatgtctgtaactcaatctgtccatgtgt
gggtgatgaggtacagtgacgctagcacacatcggaaaaacgctattactaggggaactgaacagagtagcggacgcaatgagtagtcatttaattggcggttatgagcgtg
ttcaggcggtgctatcaatcgtaatcataacagtggcagcttgatacagtgatgtcatccctgatgcgaaagcgaccgaccgacggtacatcgaatgggaatactttagggtg
atttttaagaatcgctctagggtgagtatttcccattcagctctgctccctccctctggtactttaatcaaaagcactactaaacatatgtttttaaataaaaaatattgatataga
gataatattagtaagaataattaaacaattgaatatagataaatcattgttaaataaagattaattattaaaatgaatgtatacttatatataaatcaatgatttaaaatatttga
taaagaaaacttttcaaaaaaaatataattgagattgtgtcatttcggtcaattcttaatatgttccacgcaagttttagctatggtgctaaacagaaatttgctgaaaaagaac
ttttcactgaactggttaaaatgtaagcagcctgagagccgccaaaaattttaaaaacaaaccgccttaatcatcttcaaaaaatacctctaaaacctcaccatttgcgttttaa
gacccatatttcatcctgcccttatgttcccatgctgatagctataaagtgtctgtaatcgcttcctatgacgttctaggctgttgataacttttggaacaacgcaaaatgttaaaa
tccgcctttttacggttcctggccttttgctggccttttgctcacatgttctttcctgcgttatcccctgattctgtggataaccgtattaccgcctttgagtgagctgataccgctcgc
cgcagccgaacgaccgagcgcagcgagtcagtgagcgaggaagcggaagagcgcctgatgcggtattttctccttacgcatctgtgcggtatttcacaccgcagatggtgcac
tctcagtacaatctgctctgatgccgcatagttaagccagtatacactccgctatcgctacgtgactgggtcatggctgcgccccgacacccgccaacacccgctgacgcgccct
gacgggcttgtctgctcccggcatccgcttacagacaagctgtgaccgtctccgggagctgcatgtgtcagaggttttcaccgtcatcaccgaaacgcgcgaggcagcagatca
attcgcgcgcgaaggcgaagcggcatgcatttacgttgacaccatcgaatggtgcaaaacctttcgcggtatggcatgatagcgcccggaagagagtcaattcagggtggtg
aatgtgaaaccagtaacgttatacgatgtcgcagagtatgccggtgtctcttatcagaccgtttcccgcgtggtgaaccaggccagccacgtttctgcgaaaacgcgggaaaa
agtggaagcggcgatggcggagctgaattacattcccaaccgcgtggcacaacaactggcgggcaaacagtcgttgctgattggcgttgccacctccagtctggccctgcacg
cgccgtcgcaaattgtcgcggcgattaaatctcgcgccgatcaactgggtgccagcgtggtggtgtcgatggtagaacgaagcggcgtcgaagcctgtaaagcggcggtgca
caatcttctcgcgcaacgcgtcagtgggctgatcattaactatccgctggatgaccaggatgccattgctgtggaagctgcctgcactaatgttccggcgttatttcttgatgtctc
tgaccagacacccatcaacagtattattttctcccatgaagacggtacgcgactgggcgtggagcatctggtcgcattgggtcaccagcaaatcgcgctgttagcgggcccatt
aagttctgtctcggcgcgtctgcgtctggctggctggcataaatatctcactcgcaatcaaattcagccgatagcggaacgggaaggcgactggagtgccatgtccggttttca
acaaaccatgcaaatgctgaatgagggcatcgttcccactgcgatgctggttgccaacgatcagatggcgctgggcgcaatgcgcgccattaccgagtccgggctgcgcgttg
gtgcggatatctcggtagtgggatacgacgataccgaagacagctcatgttatatcccgccgttaaccaccatcaaacaggattttcgcctgctggggcaaaccagcgtggac
cgcttgctgcaactctctcagggccaggcggtgaagggcaatcagctgttgcccgtctcactggtgaaaagaaaaaccaccctggcgcccaatacgcaaaccgcctctccccg
cgcgttggccgattcattaatgcagctggcacgacaggtttcccgactggaaagcgggcagtgagcgcaacgcaattaatgtgagttagcgcgaattgatctg

Sequence of the plasmid pCDFduet-LptB-LptF(E249K)G

gccataccgcgaaaggttttgcgccattcgatggtgtccgggatctcgacgctctcccttatgcgactcctgcattaggaaattaatacgactcactataggggaattgtgagcg
gataacaattcccctgtagaaataattttgtttaactttaataaggagatatacc**ATGGAGCAAATCGCGCAACAACAACCTCAGACTTTATGCATTAAG**
CATCTTGCAAAGAATTACAGCAAACGTTGGGTGGTAAAAGACGTATCGTTTGAGATGCAAAGTGGACAAATTGTTGGTTTGCTTG
GGCCGAATGGTGCTGGTAAAACAACCAGTTTCTATATGGTTGTCGGGTTGGTGCGCATGGATAAAGGTGAAATTCATCTTGATAA
TCTTGATTTGTCTGATCTAGCTATGCACGAACGCGCGAGAAAGGGAATTGGTTATTTGCCTCAAGAAGCTTCTATTTTCAGAAAACT
TACGATTGCTGAAAATATTATGGCTATTCTTGAGACTCGAAAAGACCTGAATAAGCAACAGCGTCAGCAACGTCTCCAAGAGTTAT
TGAATGATTTTAAAATCACGCATATTAAAGATTCTTTAGGGATGAGTGTATCTGGTGGTGAGCGACGACGTGCTGAAATTGCGCGT

GCATTGGCCGCAGACCCAAAGTTTATGCTGCTTGATGAACCTTTTGCGGGGGTGGATCCGATTTCGGTCGGTGATATTAAAGACAT
TATTCGTAATTTAAAAGATCGCGGTATTGGCGTACTCATTACCGATCATAATGTGCGTGAAACTTTGGCTATCTGTGAGCATGCTTA
CATTGTAAGTGAAGGCGCTGTAATTGCAGAGGGTTCTCCGCAGGATATTTTAGAAAATGAACAGGTACGTAAGGTATATCTAGGA
GATGATTTTACAGTCTAAgaattcgagctcggcgcgcctgcaggtcgacaagcttgcggccgcataatgcttaagtcgaacagaaagtaatcgtattgtacacggccg
cataatcgaaattaatacgactcactataggggaattgtgagcggataacaattccccatcttagtatattagttaagtataagaaggagatatacat**ATGATTATTCGA**
CGTTATCTTGTCAAACAAGTGGTATCGACGTCCTTGGTAGTGATTGCCTTATTGACCTTAATCATGATGGGTGGTCGTTTAATCAAG
TATTTTGGCGTGGCTGCTCAGGGTCGTTTGGATGCTGGCGTACTGTTTAGCATCATTGGCTATCGTATGCCTGAATTTTTAACCCTG
ATCTTACCGTTAGGATTCTTTATTGGTTTGATGCTGGTGTTTGGTCGACTTTACGTTGATCATGAAATGGCTGTACTCAATGGCAGT
GGCATCAGTCGAATTCGACTTGGACAGCTATTGATTCCACTGGCTTTGGTCTTCTTGGTGATACAAGGCATTTTGATGCTATGGATG
ACGCCTTGGGGGCTACGCCAGTTTGATCAACTCTCCAGTAGTCAGGCTGTTCGTACAGGCTTTGATTTAGTCAGACCCAAAGAATTT
ATATCATCGGGGCCTTATACCATCTATGCAGGGGATTTATCCGAAGACCGTAAAAACCTGAAAGATATTTTCTTCTACCAGCGAGC
ACAAAAAGAAGGCAAACCCGATGTCATGATTCTGGCCAAAGAAGCCACGCGTGTGGTCATGGAAAATGAAACGGCCAATGTAGT
GGACTTGATTCAGGGACGTCGCTACGAAATTTATCCAGGAAAGGCAAAATATTCACAGGCCGAATTCCAGCGCTATCGTTTACGTC
TAGAAAATGATAAGTCGGCAACCTTTGAAACTGACAAAGTTaagGCATTGCCAAGTTCAAAACTCTGGAACAAATGGAACGATCCA
GTCATTGCCAGTGAAATGGGCTGGCGTGTATTTGGCCCTTTTACCATTGTCATTGCCTTGATGATGGCTGTTGCACTGTGTGAGGTA
AGTCCGCGTCAGGGACGTTATTATCGACTTATTCCTGCGATTTTTATCTTTGCGAGTTTAATTGTATTGTTAATCGCAATTCGTACAC
GTATTAGTCGTGATGAACTAGGTGTATGGGCTTATCCAGCAGCCTTGGCTGTGTATGGTATTGCTGCGGCGTTATTCTCACGCAAG
CAAAAGCTGGCGCCTAAAATTAAGAAACAGATCAAACGAGTGAGAGCATA*ATGTTAGCACGTCGAATCGTCGCAAAGCATGTGAC*
CAAAACCACGGCGCTAGCAATGCTAGGAACCACGATTGTTTTGGTGATTTTGCAGGTTTTATTTACCTATTTAGGTGAGCTGTCCAA
TCTTAAAGCAGACTATAGCGCCTGGCAGGCATTTTTATATGTTTTATGGGGAGCACCGCGCTATCTCTATGAAATTCTGCCTATCTCG
GCGTTGATTGGTGCAATTTTAGGATTGGGTACACTGGCATCCAACAGTGAATTGATTGTGATGCGCTCGGTGGGAATCAGTTTATG
GCGGATTGTCGGTTGGGTCATTCGTTCGGCATTAGTACTGGTTTTACTCTCGTTTGCATTGAGTGAGTGGGTCGTGCCGTATACCAA
TGAGCGAGCAAATAGCGTGAAGAGCCATCAAAGTGTTGCGGCACTGGGCGAGGTACGAGGTTACTGGTCACGTGAAGGACAGCG
CTTTATTTATGTGGATTATGCCAACTCACAAGGTCAGCTTAAACGGATTCAGGTGGTCGATTTTGATGACAACTATCGTTTAAAGTC
GGTAACCAATGCCGAGCAAGGACAGTTTGTCAAAGATGGTCAATGGTTGTTAAATCATTCGCAGCAGATGGCGATTCAGGGACAA
GGCGATGCTGTATTGGCAAATGCAGCTAAACAGCCATTTTCATTGGCATTGCAGCCTAAGTACGTGCATATGGTGACGATTGATCC
AGAAGATTTATCCTTTAGCCAACTGGTCAGTTTTATGAACTATATGCGTGAATACAGCCAAGTGCCGAAAACCTATCAGTTGGCATT
CTGGAAGAAAGTGGCTTCACCTTTTGCATTGATTACGTTGGTATTGGTTGCCTGTTCTTTTATTTTTGGGCCACTACGCCAGCAATCG
ATGGGTTTTCGCTTGGTGATCGCGCTGTTTATTGGACTAGGTTTTTACTATTTACAAGATTTTCTTGGATATGCAAGTTTGGTTTATA
ACCCATCACCCGCATGGTTTGTACTTGGGCCAATTGTACTCATGTTTGTTGCGGGGAGTTACTTGTTATATCGGCAAGATAAggtacc
ctcgagtctggtaaagaaaccgctgctgcgaaatttgaacgccagcacatggactcgtctactagcgcagcttaattaacctaggctgctgccaccgctgagcaataactagc
ataaccccttggggcctctaaacgggtcttgaggggttttttgctgaaacctcaggcatttgagaagcacacggtcacactgcttccggtagtcaataaaccggtaaaccagca
atagacataagcggctatttaacgaccctgccctgaaccgacgaccgggtcatcgtggccggatcttgcggcccctcggcttgaacgaattgttagacattatttgccgactacc
ttggtgatctcgcctttcacgtagtggacaaattcttccaactgatctgcgcgcgaggccaagcgatcttcttcttgtccaagataagcctgtctagcttcaagtatgacgggctg
atactgggccggcaggcgctccattgcccagtcggcagcgacatccttcggcgcgattttgccggttactgcgctgtaccaaatgcgggacaacgtaagcactacatttcgctc
atcgccagcccagtcgggcggcgagttccatagcgttaaggtttcatttagcgcctcaaatagatcctgttcaggaaccggatcaaagagttcctccgccgctggacctaccaa
ggcaacgctatgttctcttgcttttgtcagcaagatagccagatcaatgtcgatcgtggctggctcgaagatacctgcaagaatgtcattgcgctgccattctccaaattgcagtt
cgcgcttagctggataacgccacggaatgatgtcgtcgtgcacaacaatggtgacttctacagcgcggagaatctcgctctctccaggggaagccgaagtttccaaaaggtcg
ttgatcaaagctcgccgcgttgtttcatcaagccttacggtcaccgtaaccagcaaatcaatatcactgtgtggcttcaggccgccatccactgcggagccgtacaaatgtacgg
ccagcaacgtcggttcgagatggcgctcgatgacgccaactacctctgatagttgagtcgatacttcggcgatcaccgcttccctcatactcttcctttttcaatattattgaagca
tttatcagggttattgtctcatgagcggatacatatttgaatgtatttagaaaaataaacaaatagctagctcactcggtcgctacgctccgggcgtgagactgcggcgggcgct
gcggacacatacaaagttacccacagattccgtggataagcaggggactaacatgtgaggcaaaacagcagggccgcgccggtggcgtttttccataggctccgccctcctg
ccagagttcacataaacagacgcttttccggtgcatctgtgggagccgtgaggctcaaccatgaatctgacagtacgggcgaaacccgacaggacttaaagatccccaccgtt
tccggcgggtcgctccctcttgcgctctcctgttccgaccctgccgtttaccggatacctgttccgcctttctcccttacgggaagtgtggcgctttctcatagctcacacactggta
tctcggctcggtgtaggtcgttcgctccaagctgggctgtaagcaagaactccccgttcagcccgactgctgcgccttatccggtaactgttcacttgagtccaacccggaaaag
cacggtaaaacgccactggcagcagccattggtaactgggagttcgcagaggatttgtttagctaaacacgcggttgctcttgaagtgtgcgccaaagtccggctacactgga
aggacagatttggttgctgtgctctgcgaaagccagttaccacggttaagcagttccccaactgacttaaccttcgatcaaaccacctccccaggtggttttttcgtttacagggc
aaaagattacgcgcagaaaaaaaggatctcaagaagatcctttgatcttttctactgaaccgctctagatttcagtgcaatttatctcttcaaatgtagcacctgaagtcagccc
catacgatataagttgtaattctcatgttagtcatgccccgcgcccaccggaaggagctgactgggttgaaggctctcaagggcatcggtcgagatcccggtgcctaatgagtg

agctaacttacattaattgcgttgcgctcactgcccgctttccagtcgggaaacctgtcgtgccagctgcattaatgaatcggccaacgcgcggggagaggcggtttgcgtattg
ggcgccagggtggtttttcttttcaccagtgagacgggcaacagctgattgcccttcaccgcctggccctgagagagttgcagcaagcggtccacgctggtttgccccagcagg
cgaaaatcctgtttgatggtggttaacggcgggatataacatgagctgtcttcggtatcgtcgtatcccactaccgagatgtccgcaccaacgcgcagcccggactcggtaatg
gcgcgcattgcgcccagcgccatctgatcgttggcaaccagcatcgcagtgggaacgatgccctcattcagcatttgcatggtttgttgaaaaccggacatggcactccagtcg
ccttcccgttccgctatcggctgaatttgattgcgagtgagatatttatgccagccagccagacgcagacgcgccgagacagaacttaatgggcccgctaacagcgcgatttgc
tggtgacccaatgcgaccagatgctccacgcccagtcgcgtaccgtcttcatgggagaaaataatactgttgatgggtgtctggtcagagacatcaagaaataacgccggaac
attagtgcaggcagcttccacagcaatggcatcctggtcatccagcggatagttaatgatcagcccactgacgcgttgcgcgagaagattgtgcaccgccgctttacaggcttc
gacgccgcttcgttctaccatcgacaccaccacgctggcacccagttgatcggcgcgagatttaatcgccgcgacaatttgcgacggcgcgtgcagggccagactggaggtgg
caacgccaatcggcaacgactgtttgcccgccagttgttgtgccacgcggttgggaatgtaattcagctccgccatcgccgcttccactttttcccgcgttttcgcagaaacgtgg
ctggcctggttcaccacgcgggaaacggtctgataagagacaccggcatactctgcgacatcgtataacgttactggtttcacattcaccaccctgaattgactctcttccgggc
gctatcat

Sequence of the plasmid pCDFduet-LptB-LptF(I317N)G

gccataccgcgaaaggttttgcgccattcgatggtgtccgggatctcgacgctctcccttatgcgactcctgcattaggaaattaatacgactcactataggggaattgtgagcg
gataacaattcccctgtagaaataattttgtttaactttaataaggagatatacc**ATGGAGCAAATCGCGCAACAACAACCTCAGACTTTATGCATTAAG
CATCTTGCAAAGAATTACAGCAAACGTTGGGTGGTAAAAGACGTATCGTTTGAGATGCAAAGTGGACAAATTGTTGGTTTGCTTG
GGCCGAATGGTGCTGGTAAAACAACCAGTTTCTATATGGTTGTCGGGTTGGTGCGCATGGATAAAGGTGAAATTCATCTTGATAA
TCTTGATTTGTCTGATCTAGCTATGCACGAACGCGCGAGAAAGGGAATTGGTTATTTGCCTCAAGAAGCTTCTATTTTCAGAAAACT
TACGATTGCTGAAAATATTATGGCTATTCTTGAGACTCGAAAAGACCTGAATAAGCAACAGCGTCAGCAACGTCTCCAAGAGTTAT
TGAATGATTTTAAAATCACGCATATTAAAGATTCTTTAGGGATGAGTGTATCTGGTGGTGAGCGACGACGTGCTGAAATTGCGCGT
GCATTGGCCGCAGACCCAAAGTTTATGCTGCTTGATGAACCTTTTGCGGGGGTGGATCCGATTTCGGTCGGTGATATTAAAGACAT
TATTCGTAATTTAAAAGATCGCGGTATTGGCGTACTCATTACCGATCATAATGTGCGTGAAACTTTGGCTATCTGTGAGCATGCTTA
CATTGTAAGTGAAGGCGCTGTAATTGCAGAGGGTTCTCCGCAGGATATTTTAGAAAATGAACAGGTACGTAAGGTATATCTAGGA
GATGATTTTACAGTCTAA**gaattcgagctcggcgcgcctgcaggtcgacaagcttgcggccgcataatgcttaagtcgaacagaaagtaatcgtattgtacacggccg
cataatcgaaattaatacgactcactataggggaattgtgagcggataacaattccccatcttagtatattagttaagtataagaaggagatatacat**ATGATTATTCGA
CGTTATCTTGTCAAACAAGTGGTATCGACGTCCTTGGTAGTGATTGCCTTATTGACCTTAATCATGATGGGTGGTCGTTTAATCAAG
TATTTTGGCGTGGCTGCTCAGGGTCGTTTGGATGCTGGCGTACTGTTTAGCATCATTGGCTATCGTATGCCTGAATTTTTAACCCTG
ATCTTACCGTTAGGATTCTTTATTGGTTTGATGCTGGTGTTTGGTCGACTTTACGTTGATCATGAAATGGCTGTACTCAATGGCAGT
GGCATCAGTCGAATTCGACTTGGACAGCTATTGATTCCACTGGCTTTGGTCTTCTTGGTGATACAAGGCATTTTGATGCTATGGATG
ACGCCTTGGGGGCTACGCCAGTTTGATCAACTCTCCAGTAGTCAGGCTGTTCGTACAGGCTTTGATTTAGTCAGACCCAAAGAATTT
ATATCATCGGGGCCTTATACCATCTATGCAGGGGATTTATCCGAAGACCGTAAAAACCTGAAAGATATTTTCTTCTACCAGCGAGC
ACAAAAAGAAGGCAAACCCGATGTCATGATTCTGGCCAAAGAAGCCACGCGTGTGGTCATGGAAAATGAAACGGCCAATGTAGT
GGACTTGATTCAGGGACGTCGCTACGAAATTTATCCAGGAAAGGCAAAATATTCACAGGCCGAATTCCAGCGCTATCGTTTACGTC
TAGAAAATGATAAGTCGGCAACCTTTGAAACTGACAAAGTTGAGGCATTGCCAAGTTCAAAACTCTGGAACAAATGGAACGATCC
AGTCATTGCCAGTGAAATGGGCTGGCGTGTATTTGGCCCTTTTACCATTGTCATTGCCTTGATGATGGCTGTTGCACTGTGTGAGGT
AAGTCCGCGTCAGGGACGTTATTATCGACTTATTCCTGCGATTTTTATCTTTGCGAGTTTAATTGTATTGTTAaacGCAATTCGTACAC
GTATTAGTCGTGATGAACTAGGTGTATGGGCTTATCCAGCAGCCTTGGCTGTGTATGGTATTGCTGCGGCGTTATTCTCACGCAAG
CAAAAGCTGGCGCCTAAAATTAAGAAACAGATCAAACGAGTGAGAGCATA*ATGTTAGCACGTCGAATCGTCGCAAAGCATGTGAC
CAAAACCACGGCGCTAGCAATGCTAGGAACCACGATTGTTTTGGTGATTTTGCAGGTTTTATTTACCTATTTAGGTGAGCTGTCCAA
TCTTAAAGCAGACTATAGCGCCTGGCAGGCATTTTTATATGTTTTATGGGGAGCACCGCGCTATCTCTATGAAATTCTGCCTATCTCG
GCGTTGATTGGTGCAATTTTAGGATTGGGTACACTGGCATCCAACAGTGAATTGATTGTGATGCGCTCGGTGGGAATCAGTTTATG
GCGGATTGTCGGTTGGGTCATTCGTTCGGCATTAGTACTGGTTTTACTCTCGTTTGCATTGAGTGAGTGGGTCGTGCCGTATACCAA
TGAGCGAGCAAATAGCGTGAAGAGCCATCAAAGTGTTGCGGCACTGGGCGAGGTACGAGGTTACTGGTCACGTGAAGGACAGCG
CTTTATTTATGTGGATTATGCCAACTCACAAGGTCAGCTTAAACGGATTCAGGTGGTCGATTTTGATGACAACTATCGTTTAAAGTC
GGTAACCAATGCCGAGCAAGGACAGTTTGTCAAAGATGGTCAATGGTTGTTAAATCATTCGCAGCAGATGGCGATTCAGGGACAA
GGCGATGCTGTATTGGCAAATGCAGCTAAACAGCCATTTTCATTGGCATTGCAGCCTAAGTACGTGCATATGGTGACGATTGATCC
AGAAGATTTATCCTTTAGCCAACTGGTCAGTTTTATGAACTATATGCGTGAATACAGCCAAGTGCCGAAAACCTATCAGTTGGCATT
CTGGAAGAAAGTGGCTTCACCTTTTGCATTGATTACGTTGGTATTGGTTGCCTGTTCTTTTATTTTTGGGCCACTACGCCAGCAATCG
ATGGGTTTTCGCTTGGTGATCGCGCTGTTTATTGGACTAGGTTTTTACTATTTACAAGATTTTCTTGGATATGCAAGTTTGGTTTATA***

ACCCATCACCCGCATGGTTTGTACTTGGGCCAATTGTACTCATGTTTGTTGCGGGGAGTTACTTGTTATATCGGGCAAGATAAggtacc
ctcgagtctggtaaagaaaccgctgctgcgaaatttgaacgccagcacatggactcgtctactagcgcagcttaattaacctaggctgctgccaccgctgagcaataactagc
ataaccccttggggcctctaaacgggtcttgaggggttttttgctgaaacctcaggcatttgagaagcacacggtcacactgcttccggtagtcaataaaccggtaaaccagca
atagacataagcggctatttaacgaccctgccctgaaccgacgaccgggtcatcgtggccggatcttgcggcccctcggcttgaacgaattgttagacattatttgccgactacc
ttggtgatctcgcctttcacgtagtggacaaattcttccaactgatctgcgcgcgaggccaagcgatcttcttcttgtccaagataagcctgtctagcttcaagtatgacgggctg
atactgggccggcaggcgctccattgcccagtcggcagcgacatccttcggcgcgattttgccggttactgcgctgtaccaaatgcgggacaacgtaagcactacatttcgctc
atcgccagcccagtcgggcggcgagttccatagcgttaaggtttcatttagcgcctcaaatagatcctgttcaggaaccggatcaaagagttcctccgccgctggacctaccaa
ggcaacgctatgttctcttgcttttgtcagcaagatagccagatcaatgtcgatcgtggctggctcgaagatacctgcaagaatgtcattgcgctgccattctccaaattgcagtt
cgcgcttagctggataacgccacggaatgatgtcgtcgtgcacaacaatggtgacttctacagcgcggagaatctcgctctctccaggggaagccgaagtttccaaaaggtcg
ttgatcaaagctcgccgcgttgtttcatcaagccttacggtcaccgtaaccagcaaatcaatatcactgtgtggcttcaggccgccatccactgcggagccgtacaaatgtacgg
ccagcaacgtcggttcgagatggcgctcgatgacgccaactacctctgatagttgagtcgatacttcggcgatcaccgcttccctcatactcttcctttttcaatattattgaagca
tttatcagggttattgtctcatgagcggatacatatttgaatgtatttagaaaaataaacaaatagctagctcactcggtcgctacgctccgggcgtgagactgcggcgggcgct
gcggacacatacaaagttacccacagattccgtggataagcaggggactaacatgtgaggcaaaacagcagggccgcgccggtggcgtttttccataggctccgccctcctg
ccagagttcacataaacagacgcttttccggtgcatctgtgggagccgtgaggctcaaccatgaatctgacagtacgggcgaaacccgacaggacttaaagatccccaccgtt
tccggcgggtcgctccctcttgcgctctcctgttccgaccctgccgtttaccggatacctgttccgcctttctcccttacgggaagtgtggcgctttctcatagctcacacactggta
tctcggctcggtgtaggtcgttcgctccaagctgggctgtaagcaagaactccccgttcagcccgactgctgcgccttatccggtaactgttcacttgagtccaacccggaaaag
cacggtaaaacgccactggcagcagccattggtaactgggagttcgcagaggatttgtttagctaaacacgcggttgctcttgaagtgtgcgccaaagtccggctacactgga
aggacagatttggttgctgtgctctgcgaaagccagttaccacggttaagcagttccccaactgacttaaccttcgatcaaaccacctccccaggtggttttttcgtttacagggc
aaaagattacgcgcagaaaaaaaggatctcaagaagatcctttgatcttttctactgaaccgctctagatttcagtgcaatttatctcttcaaatgtagcacctgaagtcagccc
catacgatataagttgtaattctcatgttagtcatgccccgcgcccaccggaaggagctgactgggttgaaggctctcaagggcatcggtcgagatcccggtgcctaatgagtg
agctaacttacattaattgcgttgcgctcactgcccgctttccagtcgggaaacctgtcgtgccagctgcattaatgaatcggccaacgcgcggggagaggcggtttgcgtattg
ggcgccagggtggtttttcttttcaccagtgagacgggcaacagctgattgcccttcaccgcctggccctgagagagttgcagcaagcggtccacgctggtttgccccagcagg
cgaaaatcctgtttgatggtggttaacggcgggatataacatgagctgtcttcggtatcgtcgtatcccactaccgagatgtccgcaccaacgcgcagcccggactcggtaatg
gcgcgcattgcgcccagcgccatctgatcgttggcaaccagcatcgcagtgggaacgatgccctcattcagcatttgcatggtttgttgaaaaccggacatggcactccagtcg
ccttcccgttccgctatcggctgaatttgattgcgagtgagatatttatgccagccagccagacgcagacgcgccgagacagaacttaatgggcccgctaacagcgcgatttgc
tggtgacccaatgcgaccagatgctccacgcccagtcgcgtaccgtcttcatgggagaaaataatactgttgatgggtgtctggtcagagacatcaagaaataacgccggaac
attagtgcaggcagcttccacagcaatggcatcctggtcatccagcggatagttaatgatcagcccactgacgcgttgcgcgagaagattgtgcaccgccgctttacaggcttc
gacgccgcttcgttctaccatcgacaccaccacgctggcacccagttgatcggcgcgagatttaatcgccgcgacaatttgcgacggcgcgtgcagggccagactggaggtgg
caacgccaatcggcaacgactgtttgcccgccagttgttgtgccacgcggttgggaatgtaattcagctccgccatcgccgcttccactttttcccgcgttttcgcagaaacgtgg
ctggcctggttcaccacgcgggaaacggtctgataagagacaccggcatactctgcgacatcgtataacgttactggtttcacattcaccaccctgaattgactctcttccgggc
gctatcat

Sequence of the plasmid pCDFduet-LptB-LptF(E249K I317N)G

gccataccgcgaaaggttttgcgccattcgatggtgtccgggatctcgacgctctcccttatgcgactcctgcattaggaaattaatacgactcactataggggaattgtgagcg
gataacaattcccctgtagaaataattttgtttaactttaataaggagatatacc**ATGGAGCAAATCGCGCAACAACAACCTCAGACTTTATGCATTAAG
CATCTTGCAAAGAATTACAGCAAACGTTGGGTGGTAAAAGACGTATCGTTTGAGATGCAAAGTGGACAAATTGTTGGTTTGCTTG
GGCCGAATGGTGCTGGTAAAACAACCAGTTTCTATATGGTTGTCGGGTTGGTGCGCATGGATAAAGGTGAAATTCATCTTGATAA
TCTTGATTTGTCTGATCTAGCTATGCACGAACGCGCGAGAAAGGGAATTGGTTATTTGCCTCAAGAAGCTTCTATTTTCAGAAAACT
TACGATTGCTGAAAATATTATGGCTATTCTTGAGACTCGAAAAGACCTGAATAAGCAACAGCGTCAGCAACGTCTCCAAGAGTTAT
TGAATGATTTTAAAATCACGCATATTAAAGATTCTTTAGGGATGAGTGTATCTGGTGGTGAGCGACGACGTGCTGAAATTGCGCGT
GCATTGGCCGCAGACCCAAAGTTTATGCTGCTTGATGAACCTTTTGCGGGGGTGGATCCGATTTCGGTCGGTGATATTAAAGACAT
TATTCGTAATTTAAAAGATCGCGGTATTGGCGTACTCATTACCGATCATAATGTGCGTGAAACTTTGGCTATCTGTGAGCATGCTTA
CATTGTAAGTGAAGGCGCTGTAATTGCAGAGGGTTCTCCGCAGGATATTTTAGAAAATGAACAGGTACGTAAGGTATATCTAGGA
GATGATTTTACAGTCTAA**GAATTCGAGCTCGGCGCGCCTGCAGGTCGACAAGCTTGCGGCCGCATAATGCTTAAGTCGAACAGAAA
GTAATCGTATTGTACACGGCCGCATAATCGAAATTAATACGACTCACTATAGGGGAATTGTGAGCGGATAACAATTCCCCATCTTAGT
ATATTAGTTAAGTATAAGAAGGAGATATACAT**ATGATTATTCGACGTTATCTTGTCAAACAAGTGGTATCGACGTCCTTGGTAGTGA
TTGCCTTATTGACCTTAATCATGATGGGTGGTCGTTTAATCAAGTATTTTGGCGTGGCTGCTCAGGGTCGTTTGGATGCTGGCGTAC
TGTTTAGCATCATTGGCTATCGTATGCCTGAATTTTTAACCCTGATCTTACCGTTAGGATTCTTTATTGGTTTGATGCTGGTGTTTGGT
CGACTTTACGTTGATCATGAAATGGCTGTACTCAATGGCAGTGGCATCAGTCGAATTCGACTTGGACAGCTATTGATTCCACTGGCT**

TTGGTCTTCTTGGTGATACAAGGCATTTTGATGCTATGGATGACGCCTTGGGGGCTACGCCAGTTTGATCAACTCTCCAGTAGTCAG
GCTGTTCGTACAGGCTTTGATTTAGTCAGACCCAAAGAATTTATATCATCGGGGCCTTATACCATCTATGCAGGGGATTTATCCGAA
GACCGTAAAAACCTGAAAGATATTTTCTTCTACCAGCGAGCACAAAAAGAAGGCAAACCCGATGTCATGATTCTGGCCAAAGAAG
CCACGCGTGTGGTCATGGAAAATGAAACGGCCAATGTAGTGGACTTGATTCAGGGACGTCGCTACGAAATTTATCCAGGAAAGGC
AAAATATTCACAGGCCGAATTCCAGCGCTATCGTTTACGTCTAGAAAATGATAAGTCGGCAACCTTTGAAACTGACAAAGTT<u>aag</u>G
CATTGCCAAGTTCAAAACTCTGGAACAAATGGAACGATCCAGTCATTGCCAGTGAAATGGGCTGGCGTGTATTTGGCCCTTTTACC
ATTGTCATTGCCTTGATGATGGCTGTTGCACTGTGTGAGGTAAGTCCGCGTCAGGGACGTTATTATCGACTTATTCCTGCGATTTTT
ATCTTTGCGAGTTTAATTGTATTGTTA<u>aac</u>GCAATTCGTACACGTATTAGTCGTGATGAACTAGGTGTATGGGCTTATCCAGCAGCCT
TGGCTGTGTATGGTATTGCTGCGGCGTTATTCTCACGCAAGCAAAAGCTGGCGCCTAAAATTAAGAAACAGATCAAACGAGTGAG
AGCATA*<u>ATG</u>TTAGCACGTCGAATCGTCGCAAAGCATGTGACCAAAACCACGGCGCTAGCAATGCTAGGAACCACGATTGTTTTGGT*
GATTTTGCAGGTTTTATTTACCTATTTAGGTGAGCTGTCCAATCTTAAAGCAGACTATAGCGCCTGGCAGGCATTTTTATATGTTTTA
TGGGGAGCACCGCGCTATCTCTATGAAATTCTGCCTATCTCGGCGTTGATTGGTGCAATTTTAGGATTGGGTACACTGGCATCCAAC
AGTGAATTGATTGTGATGCGCTCGGTGGGAATCAGTTTATGGCGGATTGTCGGTTGGGTCATTCGTTCGGCATTAGTACTGGTTTT
ACTCTCGTTTGCATTGAGTGAGTGGGTCGTGCCGTATACCAATGAGCGAGCAAATAGCGTGAAGAGCCATCAAAGTGTTGCGGCA
CTGGGCGAGGTACGAGGTTACTGGTCACGTGAAGGACAGCGCTTTATTTATGTGGATTATGCCAACTCACAAGGTCAGCTTAAACG
GATTCAGGTGGTCGATTTTGATGACAACTATCGTTTAAAGTCGGTAACCAATGCCGAGCAAGGACAGTTTGTCAAAGATGGTCAAT
GGTTGTTAAATCATTCGCAGCAGATGGCGATTCAGGGACAAGGCGATGCTGTATTGGCAAATGCAGCTAAACAGCCATTTTCATTG
GCATTGCAGCCTAAGTACGTGCATATGGTGACGATTGATCCAGAAGATTTATCCTTTAGCCAACTGGTCAGTTTTATGAACTATATG
CGTGAATACAGCCAAGTGCCGAAAACCTATCAGTTGGCATTCTGGAAGAAAGTGGCTTCACCTTTTGCATTGATTACGTTGGTATTG
GTTGCCTGTTCTTTTATTTTTGGGCCACTACGCCAGCAATCGATGGGTTTTCGCTTGGTGATCGCGCTGTTTATTGGACTAGGTTTTTA
CTATTTACAAGATTTTCTTGGATATGCAAGTTTGGTTTATAACCCATCACCCGCATGGTTTGTACTTGGGCCAATTGTACTCATGTTT
GTTGCGGGGAGTTACTTGTTATATCGGGCAAGATAAggtaccctcgagtctggtaaagaaaccgctgctgcgaaatttgaacgccagcacatggactcgtct
actagcgcagcttaattaacctaggctgctgccaccgctgagcaataactagcataaccccttggggcctctaaacgggtcttgaggggttttttgctgaaacctcaggcatttg
agaagcacacggtcacactgcttccggtagtcaataaaccggtaaaccagcaatagacataagcggctatttaacgaccctgccctgaaccgacgaccgggtcatcgtggcc
ggatcttgcggcccctcggcttgaacgaattgttagacattatttgccgactaccttggtgatctcgcctttcacgtagtggacaaattcttccaactgatctgcgcgcgaggcca
agcgatcttcttcttgtccaagataagcctgtctagcttcaagtatgacgggctgatactgggccggcaggcgctccattgcccagtcggcagcgacatccttcggcgcgatttt
gccggttactgcgctgtaccaaatgcgggacaacgtaagcactacatttcgctcatcgccagcccagtcgggcggcgagttccatagcgttaaggtttcatttagcgcctcaaa
tagatcctgttcaggaaccggatcaaagagttcctccgccgctggacctaccaaggcaacgctatgttctcttgcttttgtcagcaagatagccagatcaatgtcgatcgtggct
ggctcgaagatacctgcaagaatgtcattgcgctgccattctccaaattgcagttcgcgcttagctggataacgccacggaatgatgtcgtcgtgcacaacaatggtgacttcta
cagcgcggagaatctcgctctctccaggggaagccgaagtttccaaaaggtcgttgatcaaagctcgccgcgttgtttcatcaagccttacggtcaccgtaaccagcaaatcaa
tatcactgtgtggcttcaggccgccatccactgcggagccgtacaaatgtacggccagcaacgtcggttcgagatggcgctcgatgacgccaactacctctgatagttgagtcg
atacttcggcgatcaccgcttccctcatactcttcctttttcaatattattgaagcatttatcagggttattgtctcatgagcggatacatatttgaatgtatttagaaaaataaaca
aatagctagctcactcggtcgctacgctccgggcgtgagactgcggcgggcgctgcggacacatacaaagttacccacagattccgtggataagcaggggactaacatgtga
ggcaaaacagcagggccgcgccggtggcgtttttccataggctccgccctcctgccagagttcacataaacagacgcttttccggtgcatctgtgggagccgtgaggctcaacc
atgaatctgacagtacgggcgaaacccgacaggacttaaagatccccaccgtttccggcgggtcgctccctcttgcgctctcctgttccgaccctgccgtttaccggatacctgtt
ccgcctttctcccttacgggaagtgtggcgctttctcatagctcacacactggtatctcggctcggtgtaggtcgttcgctccaagctgggctgtaagcaagaactccccgttcag
cccgactgctgcgccttatccggtaactgttcacttgagtccaacccggaaaagcacggtaaaacgccactggcagcagccattggtaactgggagttcgcagaggatttgttt
agctaaacacgcggttgctcttgaagtgtgcgccaaagtccggctacactggaaggacagatttggttgctgtgctctgcgaaagccagttaccacggttaagcagttccccaa
ctgacttaaccttcgatcaaaccacctccccaggtggttttttcgtttacagggcaaaagattacgcgcagaaaaaaaggatctcaagaagatcctttgatcttttctactgaac
cgctctagatttcagtgcaatttatctcttcaaatgtagcacctgaagtcagccccatacgatataagttgtaattctcatgttagtcatgccccgcgcccaccggaaggagctga
ctgggttgaaggctctcaagggcatcggtcgagatcccggtgcctaatgagtgagctaacttacattaattgcgttgcgctcactgcccgctttccagtcgggaaacctgtcgtg
ccagctgcattaatgaatcggccaacgcgcggggagaggcggtttgcgtattgggcgccagggtggtttttcttttcaccagtgagacgggcaacagctgattgcccttcaccg
cctggccctgagagagttgcagcaagcggtccacgctggtttgccccagcaggcgaaaatcctgtttgatggtggttaacggcgggatataacatgagctgtcttcggtatcgt
cgtatcccactaccgagatgtccgcaccaacgcgcagcccggactcggtaatggcgcgcattgcgcccagcgccatctgatcgttggcaaccagcatcgcagtgggaacgatg
ccctcattcagcatttgcatggtttgttgaaaaccggacatggcactccagtcgccttcccgttccgctatcggctgaatttgattgcgagtgagatatttatgccagccagccag
acgcagacgcgccgagacagaacttaatgggcccgctaacagcgcgatttgctggtgacccaatgcgaccagatgctccacgcccagtcgcgtaccgtcttcatgggagaaa
ataatactgttgatgggtgtctggtcagagacatcaagaaataacgccggaacattagtgcaggcagcttccacagcaatggcatcctggtcatccagcggatagttaatgat
cagcccactgacgcgttgcgcgagaagattgtgcaccgccgctttacaggcttcgacgccgcttcgttctaccatcgacaccaccacgctggcacccagttgatcggcgcgaga
tttaatcgccgcgacaatttgcgacggcgcgtgcagggccagactggaggtggcaacgccaatcggcaacgactgtttgcccgccagttgttgtgccacgcggttgggaatgt

aattcagctccgccatcgccgcttccactttttcccgcgttttcgcagaaacgtggctggcctggttcaccacgcgggaaacggtctgataagagacaccggcatactctgcgac
atcgtataacgttactggtttcacattcaccaccctgaattgactctcttccgggcgctatcat

Sequence of the plasmid pCDFduet-HisLptB-LptF(R30A)G

gccataccgcgaaaggttttgcgccattcgatggtgtccgggatctcgacgctctcccttatgcgactcctgcattaggaaattaatacgactcactataggggaattgtgagcg
gataacaattcccctgtagaaataattttgtttaactttaataaggagatatacc**ATGCACCACCACCACCACCACATGGAGCAAATCGCGCAACAA**
CAACCTCAGACTTTATGCATTAAGCATCTTGCAAAGAATTACAGCAAACGTTGGGTGGTAAAAGACGTATCGTTTGAGATGCAAA
GTGGACAAATTGTTGGTTTGCTTGGGCCGAATGGTGCTGGTAAAACAACCAGTTTCTATATGGTTGTCGGGTTGGTGCGCATGGAT
AAAGGTGAAATTCATCTTGATAATCTTGATTTGTCTGATCTAGCTATGCACGAACGCGCGAGAAAGGGAATTGGTTATTTGCCTCA
AGAAGCTTCTATTTTCAGAAAACTTACGATTGCTGAAAATATTATGGCTATTCTTGAGACTCGAAAAGACCTGAATAAGCAACAGC
GTCAGCAACGTCTCCAAGAGTTATTGAATGATTTTAAAATCACGCATATTAAAGATTCTTTAGGGATGAGTGTATCTGGTGGTGAG
CGACGACGTGCTGAAATTGCGCGTGCATTGGCCGCAGACCCAAAGTTTATGCTGCTTGATGAACCTTTTGCGGGGGTGGATCCGAT
TTCGGTCGGTGATATTAAAGACATTATTCGTAATTTAAAAGATCGCGGTATTGGCGTACTCATTACCGATCATAATGTGCGTGAAA
CTTTGGCTATCTGTGAGCATGCTTACATTGTAAGTGAAGGCGCTGTAATTGCAGAGGGTTCTCCGCAGGATATTTTAGAAAATGAA
CAGGTACGTAAGGTATATCTAGGAGATGATTTTACAGTCTAAgaattcgagctcggcgcgcctgcaggtcgacaagcttgcggccgcataatgcttaagt
cgaacagaaagtaatcgtattgtacacggccgcataatcgaaattaatacgactcactataggggaattgtgagcggataacaattccccatcttagtatattagttaagtata
agaaggagatatacat**ATGATTATTCGACGTTATCTTGTCAAACAAGTGGTATCGACGTCCTTGGTAGTGATTGCCTTATTGACCTTAATC**
ATGATGGGTGGTgctTTAATCAAGTATTTTGGCGTGGCTGCTCAGGGTCGTTTGGATGCTGGCGTACTGTTTAGCATCATTGGCTAT
CGTATGCCTGAATTTTTAACCCTGATCTTACCGTTAGGATTCTTTATTGGTTTGATGCTGGTGTTTGGTCGACTTTACGTTGATCATG
AAATGGCTGTACTCAATGGCAGTGGCATCAGTCGAATTCGACTTGGACAGCTATTGATTCCACTGGCTTTGGTCTTCTTGGTGATAC
AAGGCATTTTGATGCTATGGATGACGCCTTGGGGGCTACGCCAGTTTGATCAACTCTCCAGTAGTCAGGCTGTTCGTACAGGCTTT
GATTTAGTCAGACCCAAAGAATTTATATCATCGGGGCCTTATACCATCTATGCAGGGGATTTATCCGAAGACCGTAAAAACCTGAA
AGATATTTTCTTCTACCAGCGAGCACAAAAAGAAGGCAAACCCGATGTCATGATTCTGGCCAAAGAAGCCACGCGTGTGGTCATG
GAAAATGAAACGGCCAATGTAGTGGACTTGATTCAGGGACGTCGCTACGAAATTTATCCAGGAAAGGCAAAATATTCACAGGCCG
AATTCCAGCGCTATCGTTTACGTCTAGAAAATGATAAGTCGGCAACCTTTGAAACTGACAAAGTTGAGGCATTGCCAAGTTCAAAA
CTCTGGAACAAATGGAACGATCCAGTCATTGCCAGTGAAATGGGCTGGCGTGTATTTGGCCCTTTTACCATTGTCATTGCCTTGATG
ATGGCTGTTGCACTGTGTGAGGTAAGTCCGCGTCAGGGACGTTATTATCGACTTATTCCTGCGATTTTTATCTTTGCGAGTTTAATT
GTATTGTTAATCGCAATTCGTACACGTATTAGTCGTGATGAACTAGGTGTATGGGCTTATCCAGCAGCCTTGGCTGTGTATGGTATT
GCTGCGGCGTTATTCTCACGCAAGCAAAAGCTGGCGCCTAAAATTAAGAAACAGATCAAACGAGTGAGAGCATA*ATGTTAGCACG*
TCGAATCGTCGCAAAGCATGTGACCAAAACCACGGCGCTAGCAATGCTAGGAACCACGATTGTTTTGGTGATTTTGCAGGTTTTATT
TACCTATTTAGGTGAGCTGTCCAATCTTAAAGCAGACTATAGCGCCTGGCAGGCATTTTTATATGTTTTATGGGGAGCACCGCGCTA
TCTCTATGAAATTCTGCCTATCTCGGCGTTGATTGGTGCAATTTTAGGATTGGGTACACTGGCATCCAACAGTGAATTGATTGTGAT
GCGCTCGGTGGGAATCAGTTTATGGCGGATTGTCGGTTGGGTCATTCGTTCGGCATTAGTACTGGTTTTACTCTCGTTTGCATTGAG
TGAGTGGGTCGTGCCGTATACCAATGAGCGAGCAAATAGCGTGAAGAGCCATCAAAGTGTTGCGGCACTGGGCGAGGTACGAGG
TTACTGGTCACGTGAAGGACAGCGCTTTATTTATGTGGATTATGCCAACTCACAAGGTCAGCTTAAACGGATTCAGGTGGTCGATTT
TGATGACAACTATCGTTTAAAGTCGGTAACCAATGCCGAGCAAGGACAGTTTGTCAAAGATGGTCAATGGTTGTTAAATCATTCGC
AGCAGATGGCGATTCAGGGACAAGGCGATGCTGTATTGGCAAATGCAGCTAAACAGCCATTTTCATTGGCATTGCAGCCTAAGTAC
GTGCATATGGTGACGATTGATCCAGAAGATTTATCCTTTAGCCAACTGGTCAGTTTTATGAACTATATGCGTGAATACAGCCAAGTG
CCGAAAACCTATCAGTTGGCATTCTGGAAGAAAGTGGCTTCACCTTTTGCATTGATTACGTTGGTATTGGTTGCCTGTTCTTTTATTT
TTGGGCCACTACGCCAGCAATCGATGGGTTTTCGCTTGGTGATCGCGCTGTTTATTGGACTAGGTTTTTACTATTTACAAGATTTTCT
TGGATATGCAAGTTTGGTTTATAACCCATCACCCGCATGGTTTGTACTTGGGCCAATTGTACTCATGTTTGTTGCGGGGAGTTACTT
GTTATATCGGGCAAGATAAggtaccctcgagtctggtaaagaaaccgctgctgcgaaatttgaacgccagcacatggactcgtctactagcgcagcttaattaacct
aggctgctgccaccgctgagcaataactagcataaccccttggggcctctaaacgggtcttgaggggttttttgctgaaacctcaggcatttgagaagcacacggtcacactgc
ttccggtagtcaataaaccggtaaaccagcaatagacataagcggctatttaacgaccctgccctgaaccgacgaccgggtcatcgtggccggatcttgcggcccctcggcttg
aacgaattgttagacattatttgccgactaccttggtgatctcgcctttcacgtagtggacaaattcttccaactgatctgcgcgcgaggccaagcgatcttcttcttgtccaagat
aagcctgtctagcttcaagtatgacgggctgatactgggccggcaggcgctccattgcccagtcggcagcgacatccttcggcgcgattttgccggttactgcgctgtaccaaat
gcgggacaacgtaagcactacatttcgctcatcgccagcccagtcgggcggcgagttccatagcgttaaggtttcatttagcgcctcaaatagatcctgttcaggaaccggatc
aaagagttcctccgccgctggacctaccaaggcaacgctatgttctcttgcttttgtcagcaagatagccagatcaatgtcgatcgtggctggctcgaagatacctgcaagaat
gtcattgcgctgccattctccaaattgcagttcgcgcttagctggataacgccacggaatgatgtcgtcgtgcacaacaatggtgacttctacagcgcggagaatctcgctctct

ccaggggaagccgaagtttccaaaaggtcgttgatcaaagctcgccgcgttgtttcatcaagccttacggtcaccgtaaccagcaaatcaatatcactgtgtggcttcaggccg
ccatccactgcggagccgtacaaatgtacggccagcaacgtcggttcgagatggcgctcgatgacgccaactacctctgatagttgagtcgatacttcggcgatcaccgcttcc
ctcatactcttcctttttcaatattattgaagcatttatcagggttattgtctcatgagcggatacatatttgaatgtatttagaaaaataaacaaatagctagctcactcggtcgct
acgctccgggcgtgagactgcggcgggcgctgcggacacatacaaagttacccacagattccgtggataagcaggggactaacatgtgaggcaaaacagcagggccgcgc
cggtggcgtttttccataggctccgccctcctgccagagttcacataaacagacgcttttccggtgcatctgtgggagccgtgaggctcaaccatgaatctgacagtacgggcga
aacccgacaggacttaaagatccccaccgtttccggcgggtcgctccctcttgcgctctcctgttccgaccctgccgtttaccggatacctgttccgcctttctcccttacgggaag
tgtggcgctttctcatagctcacacactggtatctcggctcggtgtaggtcgttcgctccaagctgggctgtaagcaagaactccccgttcagcccgactgctgcgccttatccgg
taactgttcacttgagtccaacccggaaaagcacggtaaaacgccactggcagcagccattggtaactgggagttcgcagaggatttgtttagctaaacacgcggttgctcttg
aagtgtgcgccaaagtccggctacactggaaggacagatttggttgctgtgctctgcgaaagccagttaccacggttaagcagttccccaactgacttaaccttcgatcaaacc
acctccccaggtggtttttttcgtttacagggcaaaagattacgcgcagaaaaaaaggatctcaagaagatcctttgatcttttctactgaaccgctctagatttcagtgcaattta
tctcttcaaatgtagcacctgaagtcagccccatacgatataagttgtaattctcatgttagtcatgccccgcgcccaccggaaggagctgactgggttgaaggctctcaagggc
atcggtcgagatcccggtgcctaatgagtgagctaacttacattaattgcgttgcgctcactgcccgctttccagtcgggaaacctgtcgtgccagctgcattaatgaatcggcc
aacgcgcggggagaggcggtttgcgtattgggcgccagggtggtttttcttttcaccagtgagacgggcaacagctgattgcccttcaccgcctggccctgagagagttgcagc
aagcggtccacgctggtttgccccagcaggcgaaaatcctgtttgatggtggttaacggcgggatataacatgagctgtcttcggtatcgtcgtatcccactaccgagatgtccg
caccaacgcgcagcccggactcggtaatggcgcgcattgcgcccagcgccatctgatcgttggcaaccagcatcgcagtgggaacgatgccctcattcagcatttgcatggttt
gttgaaaaccggacatggcactccagtcgccttcccgttccgctatcggctgaatttgattgcgagtgagatatttatgccagccagccagacgcagacgcgccgagacagaa
cttaatgggcccgctaacagcgcgatttgctggtgacccaatgcgaccagatgctccacgcccagtcgcgtaccgtcttcatgggagaaaataatactgttgatgggtgtctgg
tcagagacatcaagaaataacgccggaacattagtgcaggcagcttccacagcaatggcatcctggtcatccagcggatagttaatgatcagcccactgacgcgttgcgcga
gaagattgtgcaccgccgctttacaggcttcgacgccgcttcgttctaccatcgacaccaccacgctggcacccagttgatcggcgcgagatttaatcgccgcgacaatttgcg
acggcgcgtgcagggccagactggaggtggcaacgccaatcggcaacgactgtttgcccgccagttgttgtgccacgcggttgggaatgtaattcagctccgccatcgccgctt
ccactttttcccgcgttttcgcagaaacgtggctggcctggttcaccacgcgggaaacggtctgataagagacaccggcatactctgcgacatcgtataacgttactggtttcac
attcaccaccctgaattgactctcttccgggcgctatcat

Sequence of the plasmid pCDFduet-HisLptB-LptF(R55G)G

gccataccgcgaaaggttttgcgccattcgatggtgtccgggatctcgacgctctcccttatgcgactcctgcattaggaaattaatacgactcactataggggaattgtgagcg
gataacaattcccctgtagaaataattttgtttaactttaataaggagatatacc**ATGCACCACCACCACCACCACATGGAGCAAATCGCGCAACAA**
CAACCTCAGACTTTATGCATTAAGCATCTTGCAAAGAATTACAGCAAACGTTGGGTGGTAAAAGACGTATCGTTTGAGATGCAAA
GTGGACAAATTGTTGGTTTGCTTGGGCCGAATGGTGCTGGTAAAACAACCAGTTTCTATATGGTTGTCGGGTTGGTGCGCATGGAT
AAAGGTGAAATTCATCTTGATAATCTTGATTTGTCTGATCTAGCTATGCACGAACGCGCGAGAAAGGGAATTGGTTATTTGCCTCA
AGAAGCTTCTATTTTCAGAAAACTTACGATTGCTGAAAATATTATGGCTATTCTTGAGACTCGAAAAGACCTGAATAAGCAACAGC
GTCAGCAACGTCTCCAAGAGTTATTGAATGATTTTAAAATCACGCATATTAAAGATTCTTTAGGGATGAGTGTATCTGGTGGTGAG
CGACGACGTGCTGAAATTGCGCGTGCATTGGCCGCAGACCCAAAGTTTATGCTGCTTGATGAACCTTTTGCGGGGGTGGATCCGAT
TTCGGTCGGTGATATTAAAGACATTATTCGTAATTTAAAAGATCGCGGTATTGGCGTACTCATTACCGATCATAATGTGCGTGAAA
CTTTGGCTATCTGTGAGCATGCTTACATTGTAAGTGAAGGCGCTGTAATTGCAGAGGGTTCTCCGCAGGATATTTTAGAAAATGAA
CAGGTACGTAAGGTATATCTAGGAGATGATTTTACAGTCTAAgaattcgagctcggcgcgcctgcaggtcgacaagcttgcggccgcataatgcttaagt
cgaacagaaagtaatcgtattgtacacggccgcataatcgaaattaatacgactcactataggggaattgtgagcggataacaattccccatcttagtatattagttaagtata
agaaggagatatacat**ATGATTATTCGACGTTATCTTGTCAAACAAGTGGTATCGACGTCCTTGGTAGTGATTGCCTTATTGACCTTAATC**
ATGATGGGTGGTCGTTTAATCAAGTATTTTGGCGTGGCTGCTCAGGGTCGTTTGGATGCTGGCGTACTGTTTAGCATCATTGGCTAT
ggtATGCCTGAATTTTTAACCCTGATCTTACCGTTAGGATTCTTTATTGGTTTGATGCTGGTGTTTGGTCGACTTTACGTTGATCATGA
AATGGCTGTACTCAATGGCAGTGGCATCAGTCGAATTCGACTTGGACAGCTATTGATTCCACTGGCTTTGGTCTTCTTGGTGATACA
AGGCATTTTGATGCTATGGATGACGCCTTGGGGGCTACGCCAGTTTGATCAACTCTCCAGTAGTCAGGCTGTTCGTACAGGCTTTG
ATTTAGTCAGACCCAAAGAATTTATATCATCGGGGCCTTATACCATCTATGCAGGGGATTTATCCGAAGACCGTAAAAACCTGAAA
GATATTTTCTTCTACCAGCGAGCACAAAAAGAAGGCAAACCCGATGTCATGATTCTGGCCAAAGAAGCCACGCGTGTGGTCATGG
AAAATGAAACGGCCAATGTAGTGGACTTGATTCAGGGACGTCGCTACGAAATTTATCCAGGAAAGGCAAAATATTCACAGGCCGA
ATTCCAGCGCTATCGTTTACGTCTAGAAAATGATAAGTCGGCAACCTTTGAAACTGACAAAGTTGAGGCATTGCCAAGTTCAAAAC
TCTGGAACAAATGGAACGATCCAGTCATTGCCAGTGAAATGGGCTGGCGTGTATTTGGCCCTTTTACCATTGTCATTGCCTTGATGA
TGGCTGTTGCACTGTGTGAGGTAAGTCCGCGTCAGGGACGTTATTATCGACTTATTCCTGCGATTTTTATCTTTGCGAGTTTAATTGT
ATTGTTAATCGCAATTCGTACACGTATTAGTCGTGATGAACTAGGTGTATGGGCTTATCCAGCAGCCTTGGCTGTGTATGGTATTGC
TGCGGCGTTATTCTCACGCAAGCAAAAGCTGGCGCCTAAAATTAAGAAACAGATCAAACGAGTGAGAGCATA*ATGTTAGCACGTC*

GAATCGTCGCAAAGCATGTGACCAAAACCACGGCGCTAGCAATGCTAGGAACCACGATTGTTTTGGTGATTTTGCAGGTTTTATTT
ACCTATTTAGGTGAGCTGTCCAATCTTAAAGCAGACTATAGCGCCTGGCAGGCATTTTTATATGTTTTATGGGGAGCACCGCGCTAT
CTCTATGAAATTCTGCCTATCTCGGCGTTGATTGGTGCAATTTTAGGATTGGGTACACTGGCATCCAACAGTGAATTGATTGTGATG
CGCTCGGTGGGAATCAGTTTATGGCGGATTGTCGGTTGGGTCATTCGTTCGGCATTAGTACTGGTTTTACTCTCGTTTGCATTGAGT
GAGTGGGTCGTGCCGTATACCAATGAGCGAGCAAATAGCGTGAAGAGCCATCAAAGTGTTGCGGCACTGGGCGAGGTACGAGGT
TACTGGTCACGTGAAGGACAGCGCTTTATTTATGTGGATTATGCCAACTCACAAGGTCAGCTTAAACGGATTCAGGTGGTCGATTTT
GATGACAACTATCGTTTAAAGTCGGTAACCAATGCCGAGCAAGGACAGTTTGTCAAAGATGGTCAATGGTTGTTAAATCATTCGCA
GCAGATGGCGATTCAGGGACAAGGCGATGCTGTATTGGCAAATGCAGCTAAACAGCCATTTTCATTGGCATTGCAGCCTAAGTACG
TGCATATGGTGACGATTGATCCAGAAGATTTATCCTTTAGCCAACTGGTCAGTTTTATGAACTATATGCGTGAATACAGCCAAGTGC
CGAAAACCTATCAGTTGGCATTCTGGAAGAAAGTGGCTTCACCTTTTGCATTGATTACGTTGGTATTGGTTGCCTGTTCTTTTATTTT
TGGGCCACTACGCCAGCAATCGATGGGTTTTCGCTTGGTGATCGCGCTGTTTATTGGACTAGGTTTTTACTATTTACAAGATTTTCTT
GGATATGCAAGTTTGGTTTATAACCCATCACCCGCATGGTTTGTACTTGGGCCAATTGTACTCATGTTTGTTGCGGGGAGTTACTTG
TTATATCGGGCAAGATAAggtaccctcgagtctggtaaagaaaccgctgctgcgaaatttgaacgccagcacatggactcgtctactagcgcagcttaattaacctag
gctgctgccaccgctgagcaataactagcataaccccttggggcctctaaacgggtcttgaggggttttttgctgaaacctcaggcatttgagaagcacacggtcacactgcttc
cggtagtcaataaaccggtaaaccagcaatagacataagcggctatttaacgaccctgccctgaaccgacgaccgggtcatcgtggccggatcttgcggcccctcggcttgaa
cgaattgttagacattatttgccgactaccttggtgatctcgcctttcacgtagtggacaaattcttccaactgatctgcgcgcgaggccaagcgatcttcttcttgtccaagataa
gcctgtctagcttcaagtatgacgggctgatactgggccggcaggcgctccattgcccagtcggcagcgacatccttcggcgcgattttgccggttactgcgctgtaccaaatgc
gggacaacgtaagcactacatttcgctcatcgccagcccagtcgggcggcgagttccatagcgttaaggtttcatttagcgcctcaaatagatcctgttcaggaaccggatcaa
agagttcctccgccgctggacctaccaaggcaacgctatgttctcttgcttttgtcagcaagatagccagatcaatgtcgatcgtggctggctcgaagatacctgcaagaatgtc
attgcgctgccattctccaaattgcagttcgcgcttagctggataacgccacggaatgatgtcgtcgtgcacaacaatggtgacttctacagcgcggagaatctcgctctctcca
ggggaagccgaagtttccaaaaggtcgttgatcaaagctcgccgcgttgtttcatcaagccttacggtcaccgtaaccagcaaatcaatatcactgtgtggcttcaggccgcca
tccactgcggagccgtacaaatgtacggccagcaacgtcggttcgagatggcgctcgatgacgccaactacctctgatagttgagtcgatacttcggcgatcaccgcttccctc
atactcttcctttttcaatattattgaagcatttatcagggttattgtctcatgagcggatacatatttgaatgtatttagaaaaataaacaaatagctagctcactcggtcgctac
gctccgggcgtgagactgcggcgggcgctgcggacacatacaaagttacccacagattccgtggataagcaggggactaacatgtgaggcaaaacagcagggccgcgccg
gtggcgtttttccataggctccgccctcctgccagagttcacataaacagacgcttttccggtgcatctgtgggagccgtgaggctcaaccatgaatctgacagtacgggcgaa
acccgacaggacttaaagatccccaccgtttccggcgggtcgctccctcttgcgctctcctgttccgaccctgccgtttaccggatacctgttccgcctttctcccttacgggaagt
gtggcgctttctcatagctcacacactggtatctcggctcggtgtaggtcgttcgctccaagctgggctgtaagcaagaactccccgttcagcccgactgctgcgccttatccggt
aactgttcacttgagtccaacccggaaaagcacggtaaaacgccactggcagcagccattggtaactgggagttcgcagaggatttgtttagctaaacacgcggttgctcttg
aagtgtgcgccaaagtccggctacactggaaggacagatttggttgctgtgctctgcgaaagccagttaccacggttaagcagttccccaactgacttaaccttcgatcaaacc
acctccccaggtggttttttcgtttacagggcaaaagattacgcgcagaaaaaaaggatctcaagaagatcctttgatcttttctactgaaccgctctagatttcagtgcaattta
tctcttcaaatgtagcacctgaagtcagccccatacgatataagttgtaattctcatgttagtcatgccccgcgcccaccggaaggagctgactgggttgaaggctctcaagggc
atcggtcgagatcccggtgcctaatgagtgagctaacttacattaattgcgttgcgctcactgcccgctttccagtcgggaaacctgtcgtgccagctgcattaatgaatcggcc
aacgcgcggggagaggcggtttgcgtattgggcgccagggtggtttttcttttcaccagtgagacgggcaacagctgattgcccttcaccgcctggccctgagagagttgcagc
aagcggtccacgctggtttgccccagcaggcgaaaatcctgtttgatggtggttaacggcgggatataacatgagctgtcttcggtatcgtcgtatcccactaccgagatgtccg
caccaacgcgcagcccggactcggtaatggcgcgcattgcgcccagcgccatctgatcgttggcaaccagcatcgcagtgggaacgatgccctcattcagcatttgcatggttt
gttgaaaaccggacatggcactccagtcgccttcccgttccgctatcggctgaatttgattgcgagtgagatatttatgccagccagccagacgcagacgcgccgagacagaa
cttaatgggcccgctaacagcgcgatttgctggtgacccaatgcgaccagatgctccacgcccagtcgcgtaccgtcttcatgggagaaaataatactgttgatgggtgtctgg
tcagagacatcaagaaataacgccggaacattagtgcaggcagcttccacagcaatggcatcctggtcatccagcggatagttaatgatcagcccactgacgcgttgcgcga
gaagattgtgcaccgccgctttacaggcttcgacgccgcttcgttctaccatcgacaccaccacgctggcacccagttgatcggcgcgagatttaatcgccgcgacaatttgcg
acggcgcgtgcagggccagactggaggtggcaacgccaatcggcaacgactgtttgcccgccagttgttgtgccacgcggttgggaatgtaattcagctccgccatcgccgctt
ccactttttcccgcgttttcgcagaaacgtggctggcctggttcaccacgcgggaaacggtctgataagagacac
