# Supplementary material for: A new antibiotic traps lipopolysaccharide in its intermembrane transporter
Source: Nature. 2024 Jan 3;625(7995):572–7. doi: 10.1038/s41586-023-06799-7 (PMC10794137; doi:10.1038/s41586-023-06799-7)
Supplement: Supplementary file 2 — Reporting Summary [file 41586_2023_6799_MOESM2_ESM.pdf]

nature portfolio

Corresponding author(s): Daniel Kahne, Andrew C Kruse

Last updated by author(s): Oct 8, 2023

# Reporting Summary

Nature Portfolio wishes to improve the reproducibility of the work that we publish. This form provides structure for consistency and transparency in reporting. For further information on Nature Portfolio policies, see our Editorial Policies and the Editorial Policy Checklist.

## Statistics

For all statistical analyses, confirm that the following items are present in the figure legend, table legend, main text, or Methods section.

| n/a | Confirmed | |
|---|---|---|
| ☐ | ☒ | The exact sample size (*n*) for each experimental group/condition, given as a discrete number and unit of measurement |
| ☐ | ☒ | A statement on whether measurements were taken from distinct samples or whether the same sample was measured repeatedly |
| ☒ | ☐ | The statistical test(s) used AND whether they are one- or two-sided<br>*Only common tests should be described solely by name; describe more complex techniques in the Methods section.* |
| ☒ | ☐ | A description of all covariates tested |
| ☒ | ☐ | A description of any assumptions or corrections, such as tests of normality and adjustment for multiple comparisons |
| ☐ | ☒ | A full description of the statistical parameters including central tendency (e.g. means) or other basic estimates (e.g. regression coefficient) AND variation (e.g. standard deviation) or associated estimates of uncertainty (e.g. confidence intervals) |
| ☒ | ☐ | For null hypothesis testing, the test statistic (e.g. *F*, *t*, *r*) with confidence intervals, effect sizes, degrees of freedom and *P* value noted<br>*Give P values as exact values whenever suitable.* |
| ☒ | ☐ | For Bayesian analysis, information on the choice of priors and Markov chain Monte Carlo settings |
| ☒ | ☐ | For hierarchical and complex designs, identification of the appropriate level for tests and full reporting of outcomes |
| ☒ | ☐ | Estimates of effect sizes (e.g. Cohen's *d*, Pearson's *r*), indicating how they were calculated |

*Our web collection on statistics for biologists contains articles on many of the points above.*

## Software and code

Policy information about availability of computer code

| | |
|---|---|
| Data collection | The software used in cryoEM data collection (SerialEM v3.8.5) was accessed through the Harvard Medical School CryoEM core facility. |
| Data analysis | Structural biology applications used in this project were compiled and configured by SBGrid.<br>Cryo-EM data processing: cryoSPARC (v3.2-3.3), Relion (v3.l.4-v4.0), cryoDRGN (vl.1.0), DeepEMhancer (v20220530_cu10)<br>Model building: Coot (v0.9.4), PHENIX(vl.19.2), SwissModel (accessed via expasy server), Chimera (vl.13.1), CCP4i (v7.l.013), ISOLDE (vl.3), ChimeraX (vl.3), DAQ (v20220816_7ee5f50), Grade2 (v1.4.1, accessed via globalphasing server), eLBOW (v1.19.2-4158), molprobity (v4.02-528)<br>Biochemical experiments: GraphPad Prism (version 8.2.0) |

For manuscripts utilizing custom algorithms or software that are central to the research but not yet described in published literature, software must be made available to editors and reviewers. We strongly encourage code deposition in a community repository (e.g. GitHub). See the Nature Portfolio guidelines for submitting code & software for further information.

## Data

Policy information about availability of data

All manuscripts must include a data availability statement. This statement should provide the following information, where applicable:

- Accession codes, unique identifiers, or web links for publicly available datasets
- A description of any restrictions on data availability
- For clinical datasets or third party data, please ensure that the statement adheres to our policy

The atomic coordinates of abLptB2FG-ecLPS-1, abLptB2FG-ecLPS, abLptB2FG-ecLPS-2, abLptB2FG-ecLPS-3, abLptB2FGC, abLptB2FG-abLPS, and abLptB2FG-abLPS-1 are deposited at the Protein Data Bank with accession codes 8RFL, 8RFM, 8RFN, 8RFO, 8FRP, 8UFG and 8UFH, respectively. Cryo-EM density maps of abLptB2FG-LPS-1, abLptB2FG-LPS, abLptB2FG-ecLPS-2, abLptB2FG-ecLPS-3, abLptB2FGC, abLptB2FG-abLPS, and abLptB2FG-abLPS-1 are deposited at the Electron Microscopy Data Bank at accession codes EMD-29400, EMD-29401, EMD-29402, EMD-29403, EMD-29404, EMD-42206 and EMD-42207, respectively.

## Research involving human participants, their data, or biological material

Policy information about studies with human participants or human data. See also policy information about sex, gender (identity/presentation), and sexual orientation and race, ethnicity and racism.

| | |
|---|---|
| Reporting on sex and gender | N.A. |
| Reporting on race, ethnicity, or other socially relevant groupings | N.A. |
| Population characteristics | N.A. |
| Recruitment | N.A. |
| Ethics oversight | N.A. |

Note that full information on the approval of the study protocol must also be provided in the manuscript.

# Field-specific reporting

Please select the one below that is the best fit for your research. If you are not sure, read the appropriate sections before making your selection.

☒ Life sciences ☐ Behavioural & social sciences ☐ Ecological, evolutionary & environmental sciences

For a reference copy of the document with all sections, see nature.com/documents/nr-reporting-summary-flat.pdf

# Life sciences study design

All studies must disclose on these points even when the disclosure is negative.

| | |
|---|---|
| Sample size | Where relevant, biochemical experiments were performed in triplicate. For cryo-EM experiments, data were collected until a structure of sufficient quality was obtained. Addition of more data would not improve the resolution obtained, justifying why this sample size is sufficient. The only statistical methods used for biochemical experimentswas the calculation of standard deviations |
| Data exclusions | Through standard cryo-EM procedures, we discarded "junk" particles or classes of particles that did not yield a useful 3D reconstruction. This is accepted practice in the cryo-EM field. |
| Replication | None of the results reported could not be replicated. To ensure experimental findings could be reproduced, experiments were replicated as described in the figure caption and Methods. In summary, the blots and gels shown are representative of at least 3 independent replicates that produced similar results. The biochemical, binding, and MIC experiments were run in triplicate. |
| Randomization | Samples were not randomized - this is not relevant to the genetic, biochemical, and structural experiments performed in this work. These specific types of experiments do not necessitate randomization because they are not influenced by covariates during the sample allocation process common in other research methods. |
| Blinding | Investigators were not blinded to any data - this is not relevant to the types of experiments perform/ data generated in this work. Subjective analysis of results was not needed, so blinding was not necessary. The biochemical/biophysical data was visualized by either chemiluminescence, scintillation counting, or absorbence and quantified using standard software, which did not require subjective analysis. Structural data was also analyzed using standard software packages and did not require subjective judgment (with the only arguable exception being the choice of which particle classes were discarded as "junk", which is the accepted practice in the cryo-EM field and required to achieve high-resolution structures). |

# Reporting for specific materials, systems and methods

We require information from authors about some types of materials, experimental systems and methods used in many studies. Here, indicate whether each material, system or method listed is relevant to your study. If you are not sure if a list item applies to your research, read the appropriate section before selecting a response.

## Materials & experimental systems

| n/a | Involved in the study |
|---|---|
| ☐ | ☒ Antibodies |
| ☒ | ☐ Eukaryotic cell lines |
| ☒ | ☐ Palaeontology and archaeology |
| ☒ | ☐ Animals and other organisms |
| ☒ | ☐ Clinical data |
| ☒ | ☐ Dual use research of concern |
| ☒ | ☐ Plants |

## Methods

| n/a | Involved in the study |
|---|---|
| ☒ | ☐ ChIP-seq |
| ☒ | ☐ Flow cytometry |
| ☒ | ☐ MRI-based neuroimaging |

## Antibodies

Antibodies used

Secondary antibodies used were: sheep-anti-mouse HRP conjugate (GE AMersham, LNA931V/AH, lot #14251045, 1:10,000 dilution). Commercially available primary antibodies used were : mouse anti-His HRP conjugate (Biolegend, 652504, 1:10,000 dilution), and anti-LPS core mouse monoclonal antibody (Hycult Biotechnology, HM6011, clone WN1 222-5, lot# 18419M0715-A, 1:5,000 dilution).

Validation

Antibodies were exclusively used for western blotting. Validation of the anti-His antibody can be found on the Biolegend website (https://www.biolegend.com/fr-lu/products/hrp-anti-his-tag-antibody-9873) Certificates of analysis for the anti-mouse antibodies made by GE-Amersham can be found by lot number at https://www.gelifesciences.com/en/us/support/quality/certificates. Certificate of analysis for the anti-LPS antibody can be found at https://www.hycultbiotech.com/downloads/dl/file/id/1685/product/814/hm6011.pdf.

## Plants

Seed stocks

*Report on the source of all seed stocks or other plant material used. If applicable, state the seed stock centre and catalogue number. If plant specimens were collected from the field, describe the collection location, date and sampling procedures.*

Novel plant genotypes

*Describe the methods by which all novel plant genotypes were produced. This includes those generated by transgenic approaches, gene editing, chemical/radiation-based mutagenesis and hybridization. For transgenic lines, describe the transformation method, the number of independent lines analyzed and the generation upon which experiments were performed. For gene-edited lines, describe the editor used, the endogenous sequence targeted for editing, the targeting guide RNA sequence (if applicable) and how the editor was applied.*

Authentication

*Describe any authentication procedures for each seed stock used or novel genotype generated. Describe any experiments used to assess the effect of a mutation and, where applicable, how potential secondary effects (e.g. second site T-DNA insertions, mosiacism, off-target gene editing) were examined.*
